# PD-L1 blockade in combination with carboplatin as immune induction in metastatic lobular breast cancer: the GELATO trial

**DOI:** 10.1038/s43018-023-00542-x

**Published:** 2023-04-10

**Authors:** Leonie Voorwerk, Olga I. Isaeva, Hugo M. Horlings, Sara Balduzzi, Maksim Chelushkin, Noor A. M. Bakker, Elisa Champanhet, Hannah Garner, Karolina Sikorska, Claudette E. Loo, Inge Kemper, Ingrid A. M. Mandjes, Michiel de Maaker, Jasper J. L. van Geel, Jorianne Boers, Maaike de Boer, Roberto Salgado, Marloes G. J. van Dongen, Gabe S. Sonke, Karin E. de Visser, Ton N. Schumacher, Christian U. Blank, Lodewyk F. A. Wessels, Agnes Jager, Vivianne C. G. Tjan-Heijnen, Carolien P. Schröder, Sabine C. Linn, Marleen Kok

**Affiliations:** 1 Division of Tumor Biology and Immunology, The Netherlands Cancer Institute, Amsterdam, the Netherlands; 2 Department of Pathology, The Netherlands Cancer Institute, Amsterdam, the Netherlands; 3 Department of Biometrics, The Netherlands Cancer Institute, Amsterdam, the Netherlands; 4 Division of Molecular Carcinogenesis, The Netherlands Cancer Institute, Amsterdam, the Netherlands; 5 Oncode Institute, Utrecht, the Netherlands; 6 Department of Radiology, The Netherlands Cancer Institute, Amsterdam, the Netherlands; 7 Department of Medical Oncology, The Netherlands Cancer Institute, Amsterdam, the Netherlands; 8 Division of Molecular Pathology, The Netherlands Cancer Institute, Amsterdam, the Netherlands; 9 Department of Medical Oncology, University Medical Center Groningen, Groningen, the Netherlands; 10 Department of Medical Oncology, GROW, Maastricht University Medical Center, Maastricht, the Netherlands; 11 Department of Pathology, GZA–ZNA hospitals, Antwerp, Belgium; 12 Division of Research, Peter MacCallum Cancer Centre, Melbourne, Victoria Australia; 13 Department of Immunology, Leiden University Medical Center, Leiden, the Netherlands; 14 Division of Molecular Oncology and Immunology, The Netherlands Cancer Institute, Amsterdam, the Netherlands; 15 Department of Hematology, Leiden University Medical Center, Leiden, the Netherlands; 16 Department of Medical Oncology, Erasmus Medical Center, Rotterdam, the Netherlands; 17 Department of Pathology, University Medical Center Utrecht, Utrecht, the Netherlands

**Keywords:** Breast cancer, Immunotherapy, Tumour immunology, Cancer

## Abstract

Invasive lobular breast cancer (ILC) is the second most common histological breast cancer subtype, but ILC-specific trials are lacking. Translational research revealed an immune-related ILC subset, and in mouse ILC models, synergy between immune checkpoint blockade and platinum was observed. In the phase II GELATO trial (NCT03147040), patients with metastatic ILC were treated with weekly carboplatin (area under the curve 1.5 mg ml^–1^ min^–1^) as immune induction for 12 weeks and atezolizumab (PD-L1 blockade; triweekly) from the third week until progression. Four of 23 evaluable patients had a partial response (17%), and 2 had stable disease, resulting in a clinical benefit rate of 26%. From these six patients, four had triple-negative ILC (TN-ILC). We observed higher CD8^+^ T cell infiltration, immune checkpoint expression and exhausted T cells after treatment. With this GELATO trial, we show that ILC-specific clinical trials are feasible and demonstrate promising antitumor activity of atezolizumab with carboplatin, particularly for TN-ILC, and provide insights for the design of highly needed ILC-specific trials.

## Abstract (web summary)

Voorwerk et al. report the clinical and translational results from a phase II trial evaluating the combination of carboplatin with anti-PD-L1 in patients with invasive lobular breast cancer, who have been underrepresented in clinical trials so far.

## Main

Invasive lobular breast cancer (ILC) is the second most common histological breast cancer subtype, comprising approximately 10–15% of cases^1–3^. The non-cohesive and single file or targetoid pattern observed on routine histology is characteristic for the morphological diagnosis of ILC, and loss or aberrant expression of E-cadherin supports the diagnosis of ILC^4^. Approximately 80–90% of primary ILCs express estrogen receptor (ER), have a luminal A phenotype and can be considered classic ILC^5,6^. Approximately 5% of ILCs are triple negative (TN) and frequently exhibit a luminal phenotype, implying that this subtype has a different biology than the majority of TN breast cancer (TNBC) that is dominated by basal-like tumors^1,7,8^.

Patients with ER^+^ metastatic ILC have preferred metastatic spread to the gastrointestinal tract and bone^2,6^ and a worse overall survival than patients with ER^+^ metastatic breast cancer of no special type (NST)^1^, highlighting the need for new treatment modalities specifically for ILC. CDK4/CDK6 inhibitors combined with endocrine treatment are an effective treatment option for patients with metastatic ER^+^ ILC^9^, but no other highly effective treatment options have been defined once patients become resistant to endocrine treatment. Although ILCs are a different disease entity than NST, so far, patients with ILC have been underrepresented in clinical trials for breast cancer^10^, and reports of clinical trials specifically for ILC are lacking.

Several groups have shown that, based on transcriptomic profiling, a subgroup of ILCs can be characterized as immune related, with high levels of immune-related genes, expression of immune checkpoints and lymphocytic infiltration^5,11,12^. This suggests that a subset of ILCs might benefit from immune checkpoint blockade (ICB). While ICB in combination with chemotherapy has become standard of care in PD-L1^+^ metastatic TNBC^13^, in patients with ER^+^ breast cancer, only a small subgroup of patients benefits from ICB. Objective response rates (ORRs) to ICB monotherapy in metastatic ER^+^ breast cancer (including all histological subtypes) range from 3 to 12% (refs. ^14,15^) to 27 to 41% in combination with eribulin^16,17^. Notably, in the KEYNOTE-028 trial for patients with metastatic PD-L1^+^ER^+^ breast cancer, two of three responders were patients with ILC^15^. Rational treatment combinations are needed to improve responses to ICB in ER^+^ breast cancer and in ILC specifically.

Previous data indicate synergy between platinum compounds and ICB in genetically engineered mouse models for ILC^18^. Of note, while these models strongly resemble human ILC, the field traditionally lacks models for endocrine-sensitive ILC^19,20^. Additionally, immune-related ILCs, characterized by the expression of immune-related genes, were responsive to DNA-damaging agents, such as platinum, in vitro^11^. Mechanistically, platinum agents have been shown to trigger the cyclic GMP–AMP synthase–stimulator of interferon (IFN) genes (cGAS–STING) pathway by increasing the amounts of cytosolic DNA^21^ and to increase major histocompatibility complex class I (MHC class I) expression^22^. Based on these data, we hypothesize that the combination of platinum-based chemotherapy and ICB could be effective in patients with ILC.

Here, we report the clinical and translational results of stage I of the GELATO trial, in which patients with metastatic ILC were treated with anti-PD-L1 until disease progression, combined with low-dose carboplatin as immune induction. To dissect the immunomodulatory effects of carboplatin alone and in combination with anti-PD-L1, we profiled immune cells in the circulation and in the tumor microenvironment of longitudinal biopsies of metastatic lesions. Besides PD-L1 expression, stromal tumor-infiltrating lymphocyte (sTIL) and CD8^+^ T cell levels, deconvolution algorithms and specific immune-related gene signatures were used to dissect the effect on the various T cell populations and other elements of the cancer-immunity cycle. In addition, we studied paired primary tumors and metastatic lesions to unravel differences in the immune landscape during ILC disease progression. Finally, we studied whether carboplatin is able to modulate PD-L1 expression patterns across different metastatic lesions using molecular imaging (^89^Zr-atezolizumab-positron emission tomography (^89^Zr-atezolizumab-PET^23^)). The GELATO trial is the earliest reported clinical trial specifically conducted in patients with ILC, and our results provide insights into the biology of metastatic ILC.

## Results

### Inclusion and patient demographics

**Fig. 1:**
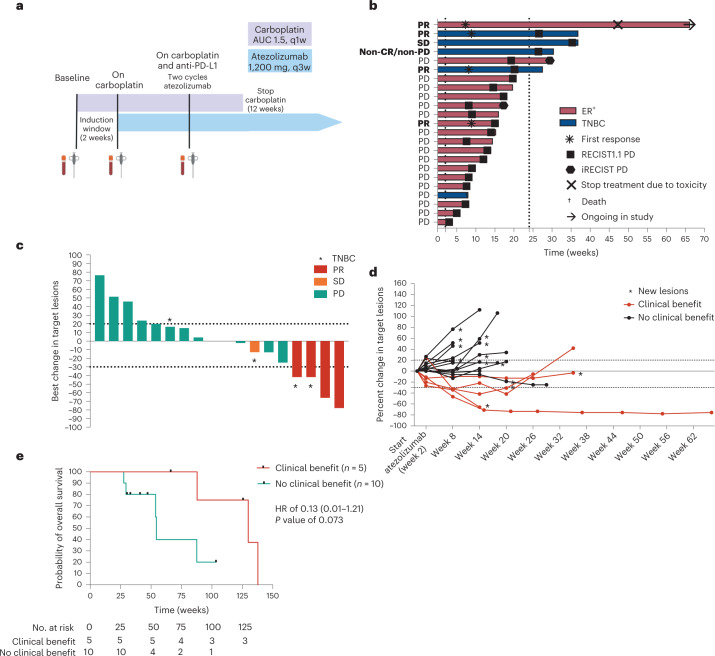
Design of the GELATO trial and efficacy data. **a**, GELATO trial setup. Patients were treated with 12 cycles of low-dose carboplatin. Atezolizumab (anti-PD-L1) was added from the third cycle onward until disease progression or toxicity. Biopsies and blood were taken at baseline before the start of anti-PD-L1 treatment and during carboplatin + anti-PD-L1 treatment. Figure created with BioRender.com; q1w, weekly; q3w, triweekly. **b**, Swimmer’s plot of all included patients; *n* = 23 patients. Each bar reflects one patient and is annotated with events indicated by the legend and clinical response according to RECISTv1.1. The dotted lines indicate the start of anti-PD-L1 at 2 weeks and the 24-week landmark of the primary endpoint. Patients with clinical benefit are depicted in bold. **c**, Waterfall plot of patients with measurable disease; *n* = 18 patients. **d**, Change in target lesions of patients with measurable disease. The *n* is as in **c**. **e**, Kaplan–Meier curve of overall survival with a 24-week landmark in patients with clinical benefit versus no clinical benefit; *n* = 15 patients. The bottom table lists numbers at risk at indicated time points. A 24-week landmark was used, causing eight patients to be removed from the analysis (one patient with clinical benefit and seven patients with PD). The HR was calculated with Cox regression analysis on the patients alive at 24 weeks, including the 95% CI and *P* value.

**Extended Data Fig. 1:**
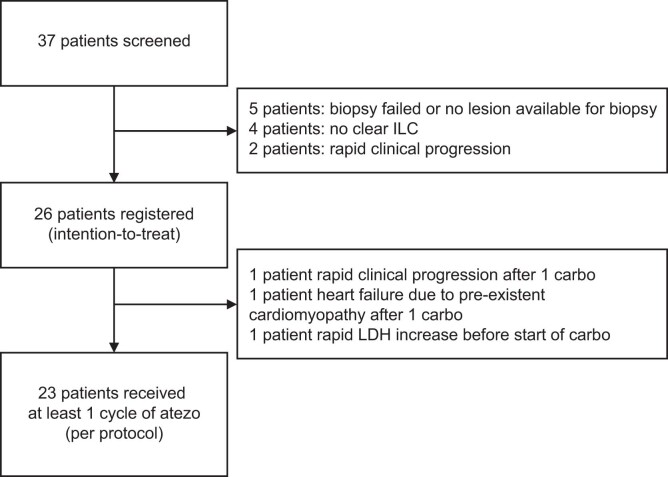
Flow chart of patient inclusion in the GELATO-trial.

In the GELATO trial, patients with metastatic ILC (based on morphology and a negative or aberrant E-cadherin staining) were treated with weekly carboplatin (area under the curve (AUC) 1.5 mg ml^–1^ min^–1^) for the first 12 weeks and atezolizumab (anti-PD-L1) every 3 weeks starting from the third cycle of carboplatin onward (Fig. 1a). The purpose of this short-term platinum-based regimen was to exploit the immunological effects of carboplatin and potentially synergize with PD-L1 blockade and not to induce direct cytotoxic effects. The low and weekly dosing was chosen to minimize the risk of hematological toxicity in this heavily pretreated population^24,25^. Following a Simon’s two-stage design, 22 patients had to be accrued in the first stage of the trial. Based on a null hypothesis of 10% of patients being progression free at 24 weeks and an alternative hypothesis of 25%, 3 of 22 patients had to be progression free at 24 weeks to allow continuation of accrual in the second stage of the trial. Between November 2017 and January 2021, 26 female patients with metastatic ILC were registered in the trial, of which 23 started anti-PD-L1 treatment (Extended Data Fig. 1), with the last 2 patients being registered simultaneously. Eighteen patients had ER^+^HER2^–^ metastatic disease, whereas five patients had TN disease (Table 1). Four of five patients with TN-ILC had ER^+^ primary ILC. Six patients had non-classical ILC based on morphological assessment of a metastatic lesion biopsy. Seventy-eight percent (*n* = 18) of patients had visceral metastases, with 52% (*n* = 12) of patients having liver metastases and 48% (*n* = 11) having three or more metastatic sites, all higher as compared to other studies^1,6^ and inherent to our eligibility criteria for biopsy site availability. Seventy-eight percent (*n* = 18) of patients received prior chemotherapy, with 52% (*n* = 12) of patients receiving prior palliative chemotherapy. Ninety-four percent (*n* = 17) of patients with ER^+^ disease received prior CDK4/CDK6 inhibitors, and 40% (*n* = 2) of patients with TN-ILC received prior platinum. Patients received a median of nine cycles of weekly carboplatin and five cycles of anti-PD-L1.

**Table 1:** Baseline characteristics of evaluable patients in the per protocol population

*n* = 23 evaluable patients		No. (%)
**Age at inclusion, years**	Median (range)	60 (45–69)
**WHO performance status**	WHO 0 WHO 1	12 (52) 11 (48)
**Histological subtype (assessed on metastatic lesion)**^a^	ER^+^HER2^–^ TNBC HER2^+^	18 (78) 5 (22) 0 (0)
**ILC subtype (assessed on metastatic lesion)**	Classic Pleiomorphic^b^ Alveolar	17 (74) 4 (17) 2 (9)
**Germline** ***BRCA1*/*BRCA2*** **mutations**	g*BRCA1* mutation No mutation Unknown	1 (4) 4 (17) 18 (78)
**Visceral metastasis**		18 (78)
**Liver metastasis**		12 (52)
**No. of metastatic sites**	1–2 metastatic sites ≥3 metastatic sites	12 (52) 11 (48)
**LDH**	LDH ≤ ULN LDH ≤ 2× ULN	15 (65) 8 (35)
**Previous chemotherapy exposure**	Chemotherapy naive (Neo)adjuvant Palliative	5 (22) 15 (65) 12 (52)
**Previous platinum treatment**	ER^+^ TNBC	0 (0) 2 (40% of TNBC)
**Previous exposure to CDK4/CDK6 inhibitors**	ER^+^ TNBC	17 (94% of ER^+^) 0 (0)
**Disease-free interval (DFI)**	De novo M1 DFI ≤ 5 years DFI > 5 years	5 (22) 12 (52) 6 (26)
**No. of cycles carboplatin**	Median (range)	9 (3–12)
**No. of cycles atezolizumab (anti-PD-L1)**	Median (range)	5 (1–16)

WHO, World Health Organization; ULN, upper limit of normal.

^a^ER^+^ ≥ 10% expression, TNBC defined as having ER and PR < 10% and HER2^–^. Four of five patients with a TN metastasis had a primary ER^+^ tumor.

^b^Two patients had TNBC.

### Efficacy

Four patients of the 23 evaluable patients (per protocol population) had a partial response (PR), leading to an ORR of 17% (95% confidence interval (95% CI) of 5–39%), with two responses being short-lived. The median duration of response was 14.9 weeks. Two additional patients had stable disease (SD) or non-complete response (non-CR)/non-progressive disease (non-PD) for at least 24 weeks, resulting in a clinical benefit rate of 26% (95% CI of 10–48%; Table 2 and Fig. 1b–d). Remarkably, four of these six patients with clinical benefit had TN-ILC (Fig. 1b). Four of the first 22 patients were free of progression at 24 weeks, meeting the primary endpoint of the first stage of the trial for which at least three responders were needed. However, as responses were generally short-lived and observed mainly in patients with TN-ILC, the trial stopped accrual after the first stage was completed. One patient has an ongoing PR even after PD-L1 blockade was stopped due to toxicity (Fig. 1b). With a median follow-up of 23.8 months, we observed a median overall survival of 54.4 weeks. Patients with clinical benefit had numerical favorable survival compared to patients with no clinical benefit, either upon analyzing patients alive after 24 weeks (Fig. 1e; hazard ratio (HR) of 0.13, *P* = 0.07) or using a time-dependent Cox model (HR of 0.26, *P* = 0.108).

**Table 2:** Efficacy analysis of evaluable patients in the per protocol population

*n* = 23 evaluable patients	
**Best overall response (RECIST1.1), no. (%)**	
CR	0 (0)
PR	4 (17)^b^
SD or non-CR/non-PD > 24 weeks^a^	2 (9)
PD	17 (74)
**ORR (CR** **+** **PR)**^b^	17% (95% CI of 5–39%)
**Clinical benefit rate (CR** **+** **PR** **+** **SD** **>** **24 weeks)**	26% (95% CI of 10–48%)
**Median duration of response**	14.9 weeks (95% CI of 6.1 weeks; not reached)
**Median progression-free survival according to RECIST1.1 (22 events)**	13 weeks (95% CI of 8.1–19.7 weeks)
**Median progression-free survival according to iRECIST (22 events)**	14 weeks (95% CI of 9.0–20.14 weeks)
**Median overall survival (16 events)**	54.4 weeks (95% CI of 23.6 weeks; not reached)

^a^One patient had SD of 24 weeks according to iRECIST.

^b^One PR was unconfirmed.

### Toxicity

Carboplatin and anti-PD-L1 were generally well tolerated, with 26% and 48% of patients, respectively, not experiencing any treatment-related adverse events (Supplementary Table 1). The most commonly observed adverse event induced by carboplatin was neutropenia, which occurred in 48% of patients (Supplementary Table 2). Anti-PD-L1 caused an increase in aspartate aminotransferase in 17% of patients, with only one patient having a grade 4 increase requiring corticosteroid treatment (Supplementary Table 3). Other relevant immune-related events were hypophysitis and colitis, occurring in two patients and one patient, respectively (Supplementary Table 3). No other endocrinopathies, such as thyroid dysfunction, were reported. One patient experienced immune-related myalgia and an immune-related sarcoid-like reaction of the mediastinal lymph nodes, cytologically confirmed granulomatous inflammation and subsequent hoarseness. This patient stopped anti-PD-L1, was treated with steroids and had an ongoing response at the time of data cutoff.

### Exploratory associations with clinical benefit

**Fig. 2:**
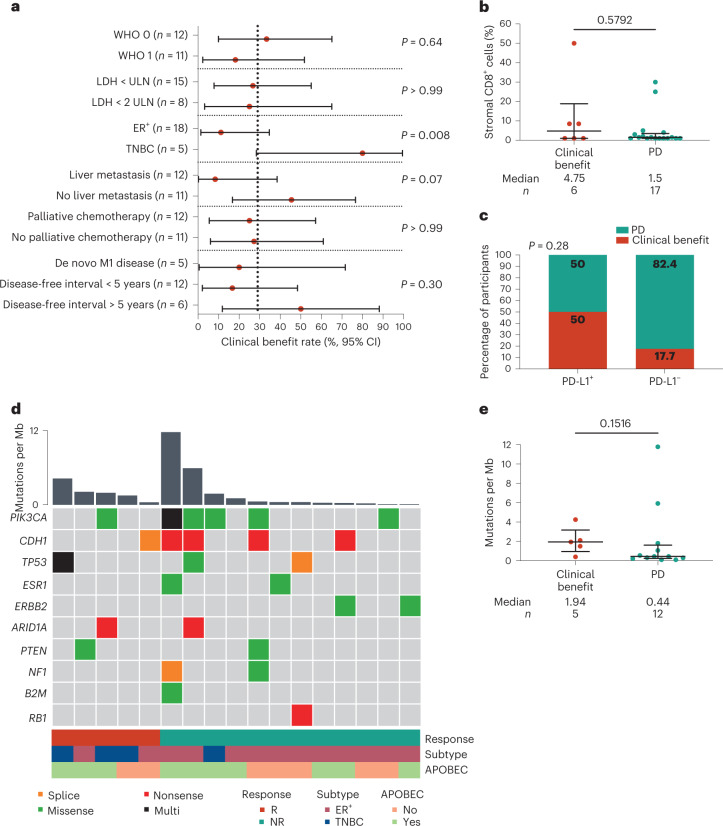
Association of baseline clinical features and characteristics of the tumor microenvironment with clinical benefit. **a**, Clinical benefit rate with 95% CI (shown as error bars) per indicated subgroup; *n* = 23 patients. Data were analyzed by two-sided Fisher’s exact test (two groups) or chi-squared test (multiple groups). **b**, Percentage of CD8^+^ cells in the stromal area of a metastatic lesion (IHC). Data are shown as median with interquartile range, and data were analyzed by two-sided Mann–Whitney *U*-test. The *n* is as in **a**. **c**, Percentage of patients with clinical benefit and PD-L1 expression (clone SP142). A cutoff of 1% expression on immune cells for PD-L1 positivity was used. Numbers in the graph indicate percentages. Data were analyzed by two-sided Fisher’s exact test. The *n* is as in **a**. **d**, Oncoplot of TMB (mutations per megabase (Mb)) and selected genes frequently altered in metastatic ILC^31–33^ assessed in biopsies of metastatic lesions. Data were available for 17 patients. Each column represents one patient and is annotated by response, subtype and enrichment of the APOBEC mutational signature; R, response; NR, no response. **e**, TMB of metastatic lesions in relation to response. The *n* is as in **d**. Data are presented as median with interquartile range. The statistics are as in **b**. In **b**–**e**, baseline metastatic lesions correspond to metastases presented in Fig. 3 and Extended Data Figs. 3 and 4.

**Extended Data Fig. 2:**
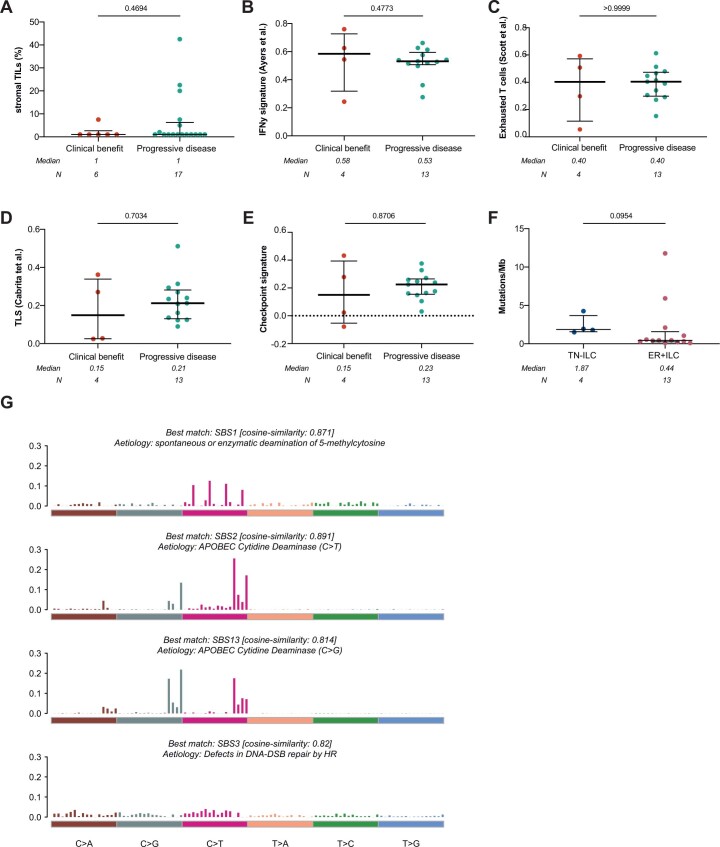
Additional baseline tumor microenvironment features associated with clinical outcome. (**A**) Percentage of stromal tumor-infiltrating lymphocytes (sTILs). *N* = 23 samples. (**B**) Gene expression of an IFNy signature^27^. *N* = 17 samples. (**C**) Gene expression of an exhausted T-cell signature^28^. *N* as in (**B**). (**D**) Gene expression of a tertiary lymphoid structure (TLS) signature^29^. *N* as in (**B**). (**E**) Gene expression of an immune checkpoint signature^30^. *N* as in (**B**). (**F**) Tumor mutational burden (TMB, mutations per MB) in ER + vs triple-negative ILC. *N* as in (**B**). (**G**) Mutational signatures enriched in metastatic lesions. *N* as in (**B**). (**A**–**F**) Median with interquartile range, statistics by two-sided Mann-Whitney-U test. Baseline metastatic lesions correspond to metastases presented in Fig. 3 and Extended Data Figs. 3 and 4.

Patients with TN-ILC had a significantly higher clinical benefit rate than patients with ER^+^ ILC (*P* = 0.008; Fig. 2a). We observed a non-significant higher clinical benefit rate in patients without liver metastases (*P* = 0.07), in line with previous findings that liver metastases might have detrimental effects on immunotherapy efficacy^26^. Looking into immune features of the metastatic lesions, we observed low baseline sTILs (median of 1%) and stromal CD8^+^ T cell levels (median of 1.5%) and no association between sTILs or CD8^+^ T cells and clinical benefit (Fig. 2b and Extended Data Fig. 2a). A higher clinical benefit rate was observed in patients with PD-L1^+^ tumors (≥1% expression on immune cells, SP142; Fig. 2c), but this was not statistically significant. Using RNA sequencing, we assessed previously established gene signatures of response to ICB. An IFNγ signature^27^, exhausted T cell signature^28^, tertiary lymphoid structure (TLS) signature^29^ and a signature capturing immune checkpoint molecules^30^ were all not significantly associated with clinical outcome (Extended Data Fig. 2b–e). Of note, the patient with an ongoing durable response at data cutoff had high levels of stromal CD8^+^ cells (50%) and relatively high expression of immune-related genes, suggesting that, although rare in ILC, patients with high immune infiltration can benefit from ICB. We observed several genomic alterations in metastatic lesions with a well-described role in metastatic ILC^31–33^, with *PIK3CA* being the most frequently mutated gene (Fig. 2d). There was a non-significantly higher total tumor mutational burden (TMB) in responders (*P* = 0.15; Fig. 2e) and in patients with TN-ILC (*P* = 0.10; Extended Data Fig. 2f). Additionally, 41% of the lesions demonstrated an APOBEC enrichment profile (Fig. 2d)^31^, and APOBEC and cytosine deamination comprised the most prominent mutational signatures enriched in the data (Extended Data Fig. 2g).

### Tumor-immune evolution of primary tumors to metastasis

**Fig. 3:**
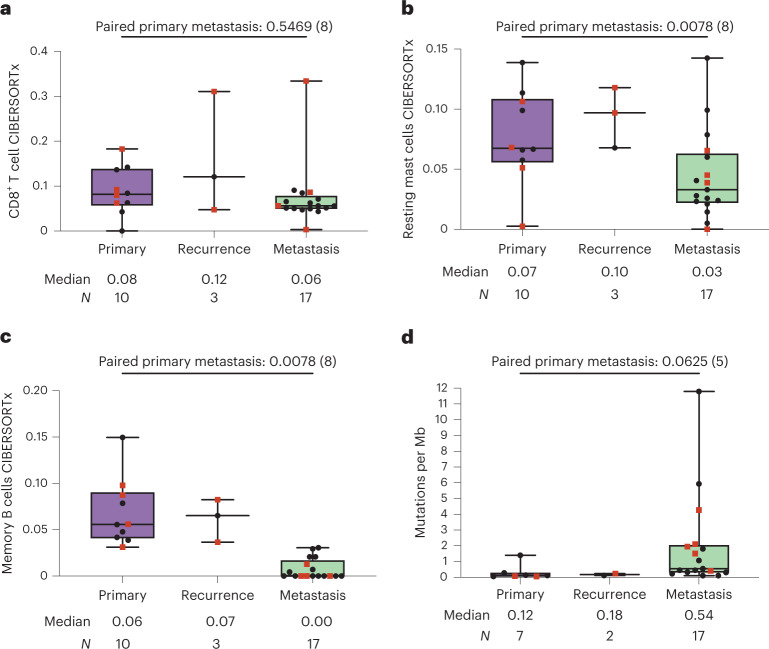
Tumor-immune evolution in paired primary tumors, local recurrences and metastasis. **a**, Gene set expression score of CD8^+^ T cells according to CIBERSORTx in paired primary tumors, recurrences and metastasis; *N* = 30 samples. **b**, Gene set expression score of resting mast cells according to CIBERSORTx in paired primary tumors, recurrences and metastasis. The *N* is as in **a**. **c**, Gene set expression score of memory B cells according to CIBERSORTx in paired primary tumors, recurrences and metastasis. The *N* is as in **a**. **d**, TMB in paired primary tumors, recurrences and metastasis; *N* = 26 samples. In **a**–**d**, box plots display a minimum (Q0), a maximum (Q4), a median (Q2) and the interquartile range. Data were analyzed by two-sided Wilcoxon signed-rank test on paired primary tumors and metastases. The numbers of patients in each analysis are listed between brackets behind the *P* value. Red squares indicate patients with clinical benefit, and black dots indicate patients with no clinical benefit. Metastatic lesions correspond with baseline samples presented in Figs. 2 and 4 and Extended Data Figs. 2, 4 and 6.

**Extended Data Fig. 3:**
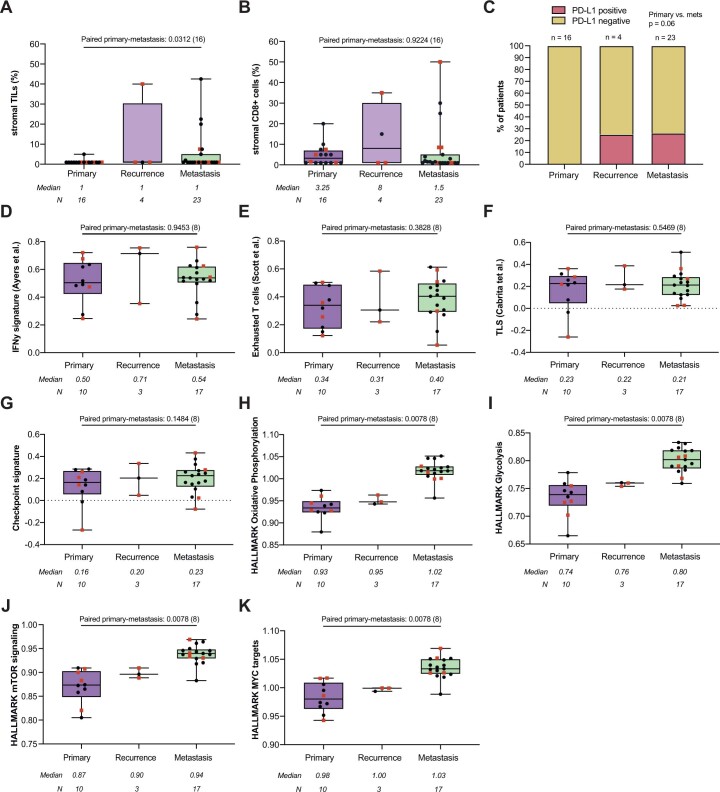
Evolution of sTILs, stromal CD8+ cells, PD-L1 expression and immune-related gene sets from paired primary tumors, local recurrences and metastasis. (**A**) Percentage of stromal tumor-infiltrating lymphocytes (sTILs) in paired primary tumors, recurrences, and metastases. *N* = 43 samples. (**B**) Percentage of CD8+ T cells in the stromal area (immunohistochemistry). *N* as in (**A**). (**C**) Percentage of patients with clinical benefit and PD-L1 expression (clone SP142) in metastatic lesions. A cut-off of 1% expression on immune cells was used to determine PD-L1 positivity. Statistics by Fisher’s exact test (primary versus metastasis) for proportions. *N* as in (**A**). (**D**) Gene expression of an IFNy signature^27^. *N* = 30 samples. (**E**) Gene expression of an exhausted T-cell signature^28^. *N* as in (**D**). (**F**) Gene expression of a tertiary lymphoid structure (TLS) signature^29^. *N* as in (**D**). (**G**) Gene expression of an immune checkpoint signature^30^. *N* as in (**D**). (**H**) Gene set enrichment score of the HALLMARK Oxidative Phosphorylation gene set. *N* as in (**D**). (**I**) Gene set enrichment score of the HALLMARK Glycolysis gene set. *N* as in (**D**). (**J**) Gene set enrichment score of HALLMARK mTOR signaling gene set. *N* as in (**D**). (**K**) Gene set enrichment score of HALLMARK MYC targets gene set. *N* as in (**D**). (**A**, **B**), (**D**–**K**) Boxplots display a minimum (Q0), a maximum (Q4), a median (Q2) and the interquartile range. Statistics with two-sided Wilcoxon-signed-rank on paired primary tumors and metastasis, the number of patients in each analysis are listed between brackets behind the p-value. Red squares indicate patients with clinical benefit, black dots patients with no clinical benefit. Metastatic lesions correspond with baseline samples presented in Fig. 2, Fig. 4, Extended Data Figs. 2, 4 and 6.

**Extended Data Fig. 4:**
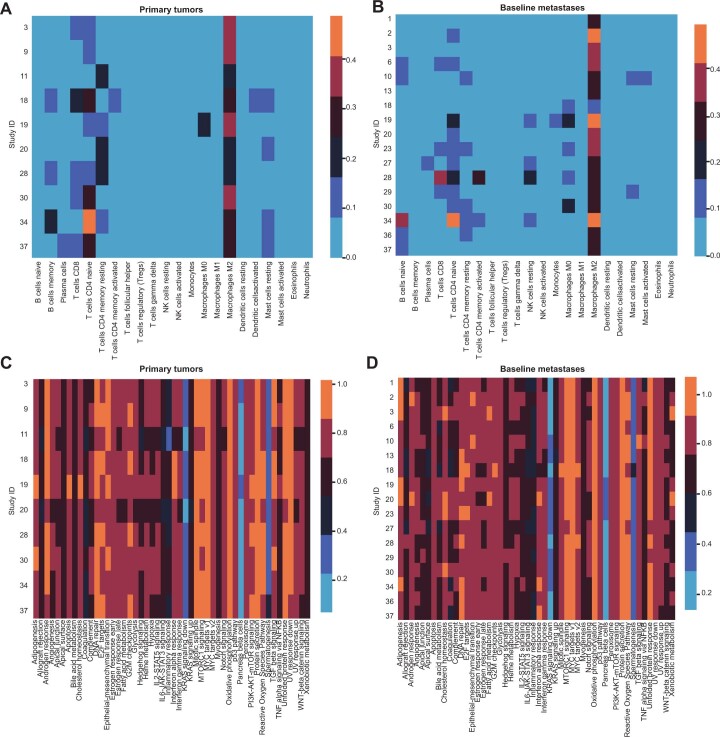
Unbiased analysis of treatment-related changes in gene expression. (**A**) Heatmap of CIBERSORTx immune cell deconvolution^34^ across all primary tumor samples (FFPE). Rows correspond to one sample and are annotated with patient ID. *N* = 10 samples. (**B**) Heatmap of CIBERSORTx immune cell deconvolution^34^ across all baseline metastases (FF). Rows correspond to one sample and are annotated with patient ID. *N* = 17 samples. (**C**) Heatmap of gene-set enrichment analysis of Hallmark gene sets^35^ across all across all primary tumor samples (FFPE). Rows correspond to one sample and are annotated with patient ID. *N* as in (**A**). (**D**) Heatmap of gene-set enrichment analysis of Hallmark gene sets^35^ across all across all baseline metastases (FF). Rows correspond to one sample and are annotated with patient ID. *N* as in (**B**).

To study the evolution of the immune landscape between matched primary lesions and metastases in ILC, we collected archival primary tumors and local recurrences (characteristics of this patient subset are in Supplementary Table 4). We observed slightly higher sTIL levels (*P* = 0.03) in metastases than in primary tumors, while this was not accompanied by a significant increase in PD-L1 and CD8^+^ T cells (Extended Data Fig. 3a–c). Using CIBERSORTx immune cell deconvolution^34^ on gene expression data, we observed little immune infiltration across tumors and across time points, with M2 macrophages being the most abundant cell type (Extended Data Fig. 4a,b), and confirmed that CD8^+^ T cell levels did not differ between paired primary and metastatic lesions (Fig. 3a). Resting mast cells and memory B cells were the only immune cell populations that were significantly lower in metastases (Fig. 3b,c). Furthermore, we applied the four previously assessed immune-related signatures and found no significant changes in expression of IFNγ-related genes, exhausted T cells, TLS or immune checkpoints (Extended Data Fig. 3d–g). Looking at differences in genomic profiles, we found non-significantly higher TMB in metastases, as previously described^31,32^ (Fig. 3d). To assess other biological differences between paired primary tumors and metastases, we performed a gene set enrichment analysis of the hallmark gene sets^35^ (Extended Data Fig. 4c,d). In metastases, we observed enrichment of glycolysis and oxidative phosphorylation gene sets (Extended Data Fig. 3h,i), indicative of increased cellular respiration, and enrichment of MYC targets and mTOR signaling (Extended Data Fig. 3j,k), suggestive of acquired signaling pathway alterations. Altogether, we observed subtle differences between primary and metastatic lesions, but the immune landscape remained largely unaffected.

### Treatment-mediated changes in circulating immune cells

**Fig. 4:**
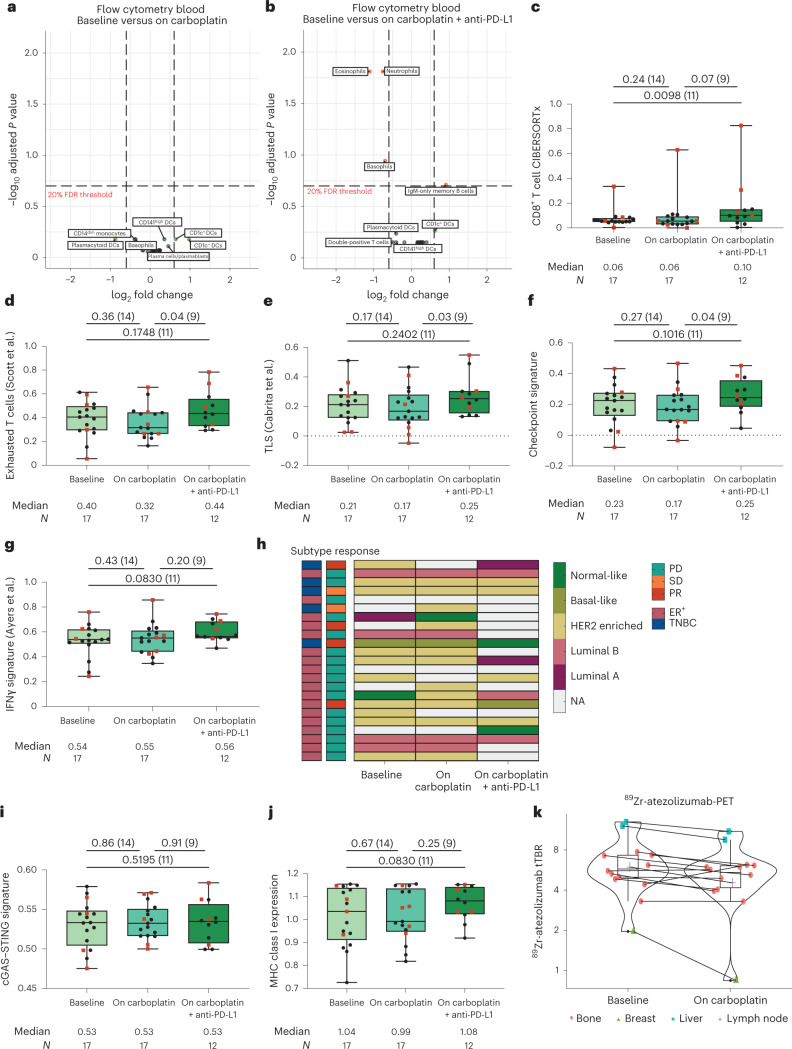
Effects of carboplatin and anti-PD-L1 on circulating immune cells and the tumor microenvironment. **a**, Volcano plot of the log_2_ (fold change) (horizontal axis) after two cycles of carboplatin to baseline in circulating immune cells, assessed by flow cytometry, and the adjusted *P* value (vertical axis). The dotted horizontal line indicates the 20% false discovery rate (FDR) threshold, and dotted vertical lines indicate a log_2_ (fold change) of 0.75. Sample pair dynamics were assessed analogously to the paired two-sided *t*-test. Multiple testing correction was performed by using the Benjamini–Hochberg procedure. For all tested populations, see Supplementary Table 5; *n* = 22 patients. **b**, Volcano plot of log_2_ (fold change) after carboplatin and anti-PD-L1 to baseline in circulating immune cells assessed by flow cytometry. Statistics are as in **a**; *n* = 18 patients. **c**, Gene set expression score of CD8^+^ T cells according to CIBERSORTx in serial metastatic biopsies taken at baseline after two cycles of carboplatin and after two cycles of anti-PD-L1 plus carboplatin; *N* = 46 samples. **d**, Gene expression of an exhausted T cell signature^28^ in serial biopsies of metastatic lesions. The *N* is as in **c**. **e**, Gene expression of a TLS signature^29^ in serial biopsies of metastatic lesions. The *N* is as in **c**. **f**, Gene expression of an immune checkpoint signature^30^ in serial biopsies of metastatic lesions. The *N* is as in **c**. **g**, Gene expression of an IFNγ signature^27^ in serial biopsies of metastatic lesions. The *N* is as in **c**. **h**, PAM50 molecular subtype assessed in serial biopsies of metastatic lesions. Each row is one patient, with the response annotated according to RECISTv1.1 and the subtype assessed on a metastatic lesion; *n* = 23 patients. NA, not applicable **i**, Gene expression of a cGAS–STING signature^42^ in serial biopsies of metastatic lesions. The *N* is as in **c**. **j**, Gene expression score of MHC class I-related genes (*HLA-A*, *HLA-B* and *HLA-C)*. The *N* is as in **c**. **k**, TBR of ^89^Zr-atezolizumab-PET at baseline and after two cycles of carboplatin in 13 lesions of one patient. In **c**–**g** and **i**–**k**, box plots display a minimum (Q0), a maximum (Q4), a median (Q2) and the interquartile range. Data were analyzed by two-sided Wilcoxon signed-rank tests on paired samples. The numbers of patients in each analysis are listed between brackets behind the *P* value. Red squares indicate patients with clinical benefit, and black dots indicate patients with no clinical benefit. Baseline metastatic lesions correspond to metastases presented in Fig. 3 and Extended Data Figs. 3 and 4.

**Extended Data Fig. 5:**
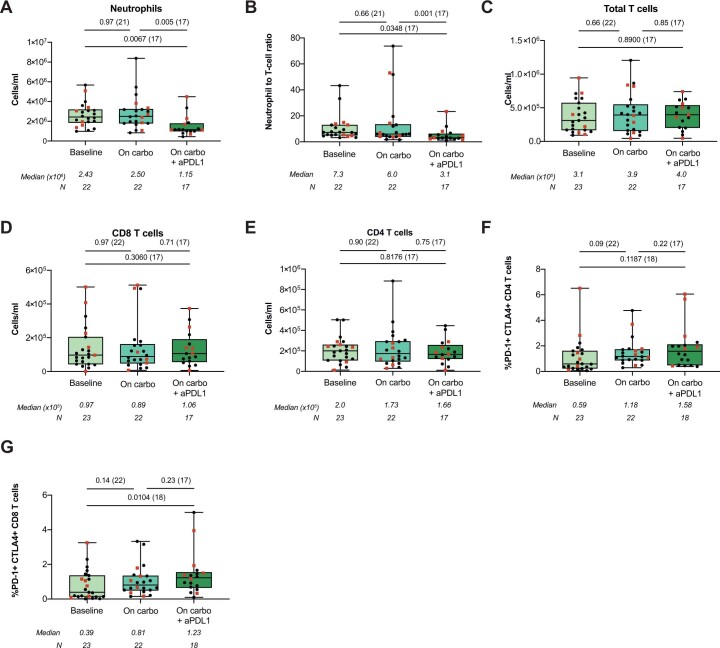
Flow cytometry-based assessment of circulating immune cell populations in paired blood samples at baseline, on carboplatin and during carboplatin/anti-PD-L1. (**A**) Absolute circulating neutrophil counts by flow cytometry. *N* = 61 samples. (**B**) Neutrophil-to-lymphocyte ratio (total T cell count). *N* as in (**A**). (**C**) Absolute circulating total T-cell counts. *N* = 62 samples. (**D**) Absolute circulating CD8+ T-cell counts. *N* as in (**C**). (**E**) Absolute circulating CD4+ T-cell counts. *N* as in (**C**). (**F**) Percentage of circulating PD-1+CTLA-4+ CD4+ T cells. *N* as in (**C**). (**G**) Percentage of circulating PD-1+CTLA-4+ CD8+ T cells. *N* as in (**C**). (**A**–**G**). Boxplots display a minimum (Q0), a maximum (Q4), a median (Q2) and the interquartile range. Statistics with two-sided Wilcoxon-signed-rank on paired samples, the number of patients in each analysis are listed between brackets behind the p-value. Red squares indicate patients with clinical benefit, black dots patients with no clinical benefit.

Several circulating immune cell populations can be affected by ICB, resulting in increased exhausted T cells and eosinophils or a decreased neutrophil-to-lymphocyte ratio^36–38^. To investigate this in ILC, we characterized absolute counts of immune cell populations in fresh blood by flow cytometry at baseline, during carboplatin treatment and during carboplatin + anti-PD-L1 treatment (Supplementary Table 5). After two cycles of carboplatin, no major changes were observed in circulating immune cells (Fig. 4a), but after treatment with carboplatin and anti-PD-L1, we observed a significant decrease in neutrophils, basophils, eosinophils and the neutrophil-to-lymphocyte ratio, probably related to the cumulative carboplatin effect (Fig. 4b and Extended Data Fig. 5a,b). Circulating total T cell and CD4^+^ and CD8^+^ T cell levels remained unaffected (Extended Data Fig. 5c–e), but we observed a significant increase in circulating PD-1^+^CTLA4^+^CD8^+^ T cells upon treatment with carboplatin and anti-PD-L1 (Extended Data Fig. 5f,g). This suggests systemic reinvigoration of a dysfunctional or exhausted T cell population that is frequently used as a proxy for the presence of a tumor-reactive T cell compartment^39^.

### Treatment-induced changes in the tumor microenvironment

**Extended Data Fig. 6:**
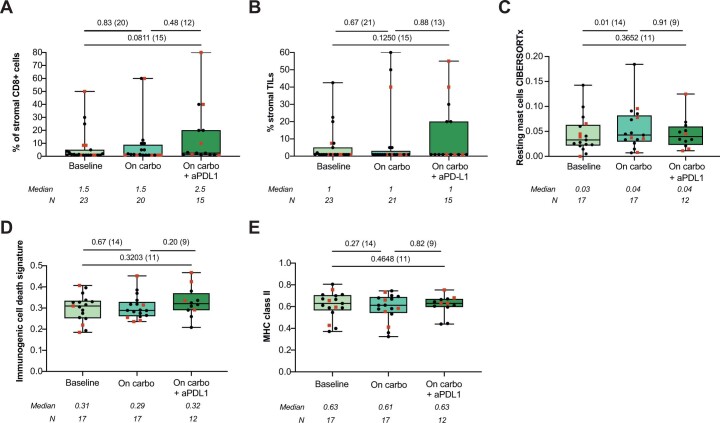
Changes in sTILs, stromal CD8+ cells and immune-related gene sets in serial biopsies of a metastatic lesion. (**A**) Percentage of CD8+ T cells (immunohistochemistry) in the stromal area in serial biopsies of metastatic lesions measured at baseline, after two cycles of carboplatin and after two cycles of anti-PD-L1 plus carboplatin. *N* = 58 samples. (**B**) Percentage of stromal tumor-infiltrating lymphocytes (sTILs) in serial biopsies of metastatic lesions. *N* = 59 samples. (**C**) Gene set expression score of resting mast cells according to CIBERSORTx in serial biopsies of metastatic lesions. *N* = 46 samples. (**D**) Gene expression of an immunogenic cell death signature^45^ in serial biopsies of metastatic lesions. *N* as in (**C**). (**E**) Gene expression score of MHC class II related genes (*HLA-DRA, HLA-DRB1, HLA-DOB, HLA-DPB2, HLA-DMA, HLA-DOA, HLA-DPA1, HLA-DPB1, HLA-DMB, HLA-DQB1, HLA-DQA1, HLA-DRB5, HLA-DQA2, HLA-DQB2, HLA-DRB6)*. *N* as in (**C**). (**A**–**E**) Boxplots display a minimum (Q0), a maximum (Q4), a median (Q2) and the interquartile range. Statistics with two-sided Wilcoxon-signed-rank on paired samples, the number of patients in each analysis are listed between brackets behind the p-value. Red squares indicate patients with clinical benefit, black dots patients with no clinical benefit. Baseline metastatic lesions correspond to metastases presented in Fig. 3 and Extended Data Figs. 3 and 4.

Next, we assessed treatment-induced changes by carboplatin and anti-PD-L1 within the tumor microenvironment of ILC metastases. Using CIBERSORTx immune cell deconvolution^34^, we observed increased CD8^+^ T cells during anti-PD-L1 treatment, most notably in the patient with a durable response (Fig. 4c), and the same pattern was seen when analyzing CD8 by immunohistochemistry (IHC; Extended Data Fig. 6a). sTIL levels remained largely unaffected (Extended Data Fig. 6b). Interestingly, while mast cells decreased during ILC disease progression (Fig. 3b), resting mast cells increased during carboplatin treatment (Extended Data Fig. 6c). Next, we assessed immune-related gene signatures and observed a significant increase after carboplatin and anti-PD-L1 in exhausted T cells^28^, TLSs^29^ and immune checkpoint expression^30^ and a trend toward a higher IFNγ signature score but only when compared to the on-carboplatin time point (before the start of anti-PD-L1), indicating a subtle decrease of these signatures after carboplatin treatment alone (Fig. 4d–g). Next, we investigated changes in PAM50 molecular subtype during treatment in the metastatic setting. We observed that the majority of tumors (59%; 10 of 17) were classified as HER2 enriched at baseline (Fig. 4h), while patients had no HER2 overexpression or amplification. Notably, we observed that the PAM50 subtype changed in 6 of 16 patients during treatment with carboplatin with or without anti-PD-L1, of which 3 were responders. High proportions of HER2-enriched metastases have been observed before in breast cancer, possibly due to disease progression in a more aggressive phenotype^40,41^. Finally, we tested our preclinical hypotheses on immunogenic effects of carboplatin and, surprisingly, did not see alterations in gene signatures for cGAS–STING^42^, immunogenic cell death^43^, MHC class I or MHC class II (Fig. 4i,j and Extended Data Fig. 6d,e). In conclusion, induction with two cycles of carboplatin did not lead to major changes in the TME, but the combination of carboplatin and anti-PD-L1 was able to induce immune infiltration by CD8^+^ T cells and increase expression of immune-related genes.

### PD-L1 uptake after carboplatin by ^89^Zr-atezolizumab-PET

**Extended Data Fig. 7:**
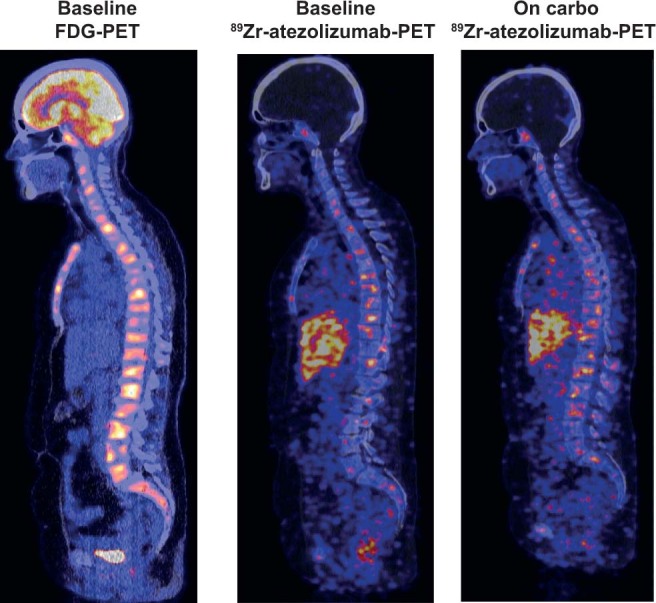
Exploratory analysis of the use ^89^Zr-atezolizumab-PET to evaluate PD-L1 distribution in ILC patients. Representative images of one patient imaged with FDG-PET and ^89^Zr-atezolizumab-PET. Left panel represents lateral view of baseline FDG-PET, the middle panel represents the lateral view of baseline ^89^Zr-atezolizumab-PET and the right panel ^89^Zr-atezolizumab-PET after two cycles of carboplatin.

To investigate the effect of carboplatin on the tumor microenvironment in a non-invasive fashion, which could be particularly attractive for ILC where biopsies can be challenging to obtain, we explored the use of ^89^Zr-atezolizumab-PET. Repeated ^89^Zr-atezolizumab-PET could be performed in one patient who had 2 measurable lesions on computed tomography (CT) scan (breast and liver) and 12 other lesions on fluorodeoxyglucose-PET (FDG-PET) at baseline (Extended Data Fig. 7). Heterogeneous ^89^Zr-atezolizumab uptake between the lesions was observed at baseline and after two cycles of carboplatin. Contrary to the hypothesis of PD-L1 induction by carboplatin, but in line with lack of clinical treatment benefit in this patient, the median tumor-to-blood ratio (TBR) decreased after induction treatment (*P* = 0.01; Fig. 4k), particularly in the index breast lesion. Meanwhile the maximal standardized uptake value (*SUV*_max_) of this lesion remained low (2.13 and 1.2, respectively), in line with its negative PD-L1 IHC (0% in immune cells) at baseline and after carboplatin treatment. In conclusion, repeated ^89^Zr-atezolizumab-PET showed heterogeneity in the dynamics of tracer uptake in tumor lesions and background during carboplatin treatment.

## Discussion

To our knowledge, the GELATO trial is the earliest reported clinical trial conducted specifically in patients with ILC based on a hypothesis founded on preclinical and translational data. While carboplatin alone neither led to significant changes in immune cell composition nor in an increase in cGAS–STING signaling or MHC class I expression, the addition of anti-PD-L1 caused an increase in CD8^+^ T cell infiltration and higher expression of immune-related gene signatures. Four of the first 22 patients were progression free at 24 weeks in the first stage of the trial, warranting expansion of the trial according to the Simon’s two-stage design. However, responses were mainly observed in patients with TN-ILC, and responses were not durable. This suggests that most responses could have been mainly induced by carboplatin and to a lesser extent by anti-PD-L1, as carboplatin monotherapy is effective in approximately 30% of patients with metastatic TNBC^24^. Because ICB plus chemotherapy is now standard of care for patients with PD-L1^+^ (≥10% combined positive score, 22C3) metastatic TNBC^13^, regardless of histological subtype, the study team decided, despite meeting the success criteria for stage I, not to proceed to the next stage of the GELATO trial. The lack of responses to anti-PD-L1 in ER^+^ ILC could be partially explained by not preselecting patients based on a preexisting antitumor immune phenotype. Important in this context is that in prior studies, the vast majority of immune-related ILCs were ER^+^. We illustrate this by one patient with ER^+^ ILC with a clear durable response of over 1 year, with a tumor microenvironment characterized by high sTIL and CD8^+^ T cell levels and positive PD-L1 expression at baseline. This indicates that, although rare, patients with ILC with an immunogenic phenotype might benefit from ICB.

Recent research has suggested that TN-ILCs have different biological characteristics than TN-NST and ER^+^ ILC, with increased androgen receptor (AR) signaling and a higher frequency of *HER2* mutations^8,44^. Although approximately 2% of patients with primary ILC and 12–15% of patients with metastatic ILC harbor a *HER2* mutation and 90% of primary ILCs are considered AR^+^^45^, among TN-ILC, 20% of the tumors harbor a *HER2* mutation, and 74–94% of tumors express AR^8,44^. In GELATO, four of five patients with TN-ILC had ER^+^ primary tumors, and all patients with TN-ILC had positive AR IHC expression (≥10% of tumor cells). Recently, it has been shown that AR inhibition and ICB synergize in vivo by reduced suppression of *Ifng* via AR signaling in CD8^+^ T cells^46^. Also, estrogen signaling has been negatively associated with response to ICB and chemotherapy in metastatic TNBC^47^ and metastatic ER^+^ breast cancer^48^. Recently, it has been shown that estrogen might polarize tumor-associated macrophages toward an immunosuppressive state in melanoma models^49^. Polarized tumor-associated macrophages have been associated with residual disease after chemotherapy in ER^+^ breast cancer^50^ and with poor survival in individuals with ILC^51^. In our CIBERSORTx analysis, we indeed found M2 macrophages as the most abundant cell type across samples (Extended Data Fig. 4a,b). Therefore, targeting AR or macrophages might help to overcome ICB resistance in ILC.

Only one patient with TN-ILC was classified as basal like by PAM50 in the metastatic setting, and four of five patients with TN-ILC had ER^+^ primary tumors. This implies that although ER expression was lost during disease progression, TN-ILCs do not exhibit a clear basal-like phenotype. A basal-like phenotype has been associated with response to ICB and chemotherapy in early-stage high-risk ER^+^ breast cancer^52^ and a basal-like immune-activated phenotype in metastatic TNBC^47^.

Interestingly, most metastases were classified as HER2 enriched. This might be an artifact of PAM50 assessment on metastatic lesions and/or fresh-frozen (FF) material. However, a particularly high proportion of HER2-enriched tumors was also observed in paired lesions of the AURORA program for metastatic breast cancer and another retrospective series^40,41^. The high level of HER2-enriched lesions might be due to the more aggressive features of metastatic disease and endocrine treatment-refractory disease potentially losing its luminal features after disease progression^40^. Furthermore, in the recent BioPER trial, after treatment with a CDK4/CDK6 inhibitor, 37.5% of the samples showed a HER2-enriched subtype^53^, suggesting that HER2-enriched tumors are prominent in patients that are heavily pretreated and/or that have been exposed to CDK4/CDK6 inhibition, as was the case for 94% of ER^+^ GELATO patients. Interestingly, in GELATO, 90% of primary tumors were classified as either luminal A or luminal B (Supplementary Table 4), suggestive of an acquired HER2-enriched phenotype later in the disease course.

During treatment with carboplatin with or without anti-PD-L1, we also observed PAM50 subtype switching in 6 of 16 patients, of which 5 switched toward a luminal or normal-like phenotype. Because we studied serial biopsies of the same lesions, this suggests that treatment modified the tumor-intrinsic characteristics of these lesions toward a less proliferative phenotype. Most notably, the baseline metastatic lesion of the patient with a durable response was characterized as HER2 enriched but switched to a basal-like phenotype during anti-PD-L1 treatment, suggesting increased susceptibility to ICB.

On the immune cell level, we observed higher levels of CD8^+^ T cells in the stroma as compared to sTILs. As CD8^+^ T cells comprise the most prevalent immune cell type captured by the sTIL readout^54^, this is surprising. Because both sTIL and CD8^+^ T cell counts were scored by the same independent expert pathologists, it is unlikely that interrater variability played a major role here. Alternatively, we hypothesize that based on their morphology, sTILs can be easily misunderstood for tumor cells in ILC, and sTILs might therefore not be the appropriate readout for antitumor immunity in ILC. Research comparing sTILs in ILC with other subtypes might have underestimated sTIL scores in ILC, and incorporating a CD8 staining may improve immune assessment in patients with ILC.

Our trial is limited by a small sample size and lack of a control arm. Of note, the inclusion of a relatively small number of patients (*n* = 23) in high-volume breast cancer centers took approximately 3.5 years. In view of the priority for translational research, patients with bone-only disease or only small lesions in, for example, the peritoneum could not participate due to the lack of an available biopsy site, which might have slowed down inclusion. Because serial biopsies were mandatory in the trial, we included a relatively high proportion of patients with ILC with visceral metastasis (18 of 23), higher than the general ILC population^1^, making our cohort not fully representative of the general ILC population. Additionally, the included patients comprise a heterogenous group characterized by different pretreatment regimens, biopsy locations and hormone receptor status. Due to the small number of patients, our translational analyses should be considered exploratory. However, given the strong preclinical rationale behind the GELATO trial, we would like to highlight the importance of the validation of preclinical findings, which was the main reason to execute the GELATO trial.

Although ILCs comprise a separate disease entity within the breast cancer subtypes, so far, reports of clinical trials specific for patients with ILC are lacking^55^, and patients with metastatic ILC are often underrepresented because of a lack of measurable disease^10^. In our experience, several patients stopped treatment early due to rapid clinical progression (Extended Data Fig. 1). These aspects of the disease complicate the inclusion of patients with ILC in clinical trials. Moreover, to our knowledge, only two reports of recent randomized trials for new treatment options in ER^+^ metastatic breast cancer have presented prespecified subgroup analysis in patients with ILC. Benefit from the addition of CDK4/CDK6 inhibition to endocrine treatment was demonstrated in patients with metastatic ER^+^ ILC^9^. More recently, a high clinical benefit rate was observed for neratinib and fulvestrant in *HER2*-mutated ILC in the phase II MutHER trial^56^. Targetable features of ILC are, for example, high expression of the ER/luminal A phenotype, synthetic-lethal deficiency of ROS1/E-cadherin^57^, high TMB^31,32^, high T cell infiltration^5,11,12^ and downstream activation of the PI3K–AKT–mTOR pathway via activating *PIK3CA* mutations^5,31^, activating *HER2* mutations^31,33^ or activation via the insulin-like growth factor 1 receptor^58^. Some of these concepts are currently under investigation in ILC-specific clinical trials, such as inducing synthetic lethality with ROS1 inhibitors in E-cadherin^–^ cells in both early-stage and metastatic ILC (NCT04551495 and NCT03620643), exploiting sensitivity to neoadjuvant endocrine treatment in early-stage ILC (NCT02206984 and NCT01953588) or in combination with CDK4/CDK6 inhibition (NCT02764541) and targeting activating *HER2* mutations in metastatic ILC with neratinib and fulvestrant in a basket of the SUMMIT trial (NCT01953926). Additionally, based on preclinical data and our work presented here, targeting the PI3K–AKT–mTOR pathway^59^ or macrophages^60^ in combination with ICB and/or chemotherapy might be promising treatment strategies for patients with ILC. Besides trials specific for ILC, subgroup analyses of patients with ILC in randomized clinical trials are of vital importance to inform treatment decisions for patients with ILC and thereby improve outcome for this difficult-to-treat breast cancer subtype.

In conclusion, we report on a clinical trial specific for metastatic ILC representing a difficult-to-treat breast cancer subtype, and we demonstrate that the combination of carboplatin and anti-PD-L1 induces clinical and immunological responses in a subset of patients with ILC. Most of the responses were observed in patients with TN-ILC, highlighting that patients with TNBC should be considered for ICB regardless of histological subtype. Our work provides hypotheses and paves the way for highly needed ILC-specific clinical trials.

## Methods

### Study design

The GELATO trial was conducted in accordance with Good Clinical Practice guidelines and the Declaration of Helsinki. All patients provided written informed consent. The trial protocol, informed consent form and amendments were approved by the medical ethical committee of the Netherlands Cancer Institute (NKI). GELATO is a phase II, single-arm, multicenter clinical trial conducted at four centers in the Netherlands (NCT03147040) to evaluate the efficacy of carboplatin and atezolizumab (anti-PD-L1) in patients with metastatic ILC. Lobular histology needed to be confirmed on a biopsy of a metastatic lesion with negative or aberrant E-cadherin IHC staining. Eligible patients were treated with 12 cycles of weekly carboplatin (AUC of 1.5 mg ml^–1^ min^–1^) and atezolizumab (anti-PD-L1; 1,200 mg flat dose) every 3 weeks starting from the third cycle of carboplatin onward (Fig. 1a). The purpose of this short-term, low-dose platinum regimen was to exploit the immunological effects of carboplatin instead of establishing a direct cytotoxic effect and avoid potential prolonged bone marrow suppression. As responses to immunotherapy in the metastatic breast cancer setting are predominantly observed within 12 weeks^15^, the duration of carboplatin induction treatment was limited to 12 weeks (see trial protocol in Supplementary Note).

Anti-PD-L1 was continued until disease progression according to RECISTv1.1 (ref. ^61^), clinical progression or unacceptable toxicity. Before the start of carboplatin, after two cycles of carboplatin (2 weeks from baseline) and after two cycles of anti-PD-L1 (plus six weekly administrations of carboplatin, 8 weeks from baseline), blood was drawn, and sequential biopsies from a metastatic lesion were taken. The first six patients were included in a 3 + 3 phase Ib safety run-in part with the same treatment schedule and were included in the total number of participants. This investigator-initiated trial was sponsored by the NKI, and atezolizumab was provided by Roche. Patients were not financially compensated for their involvement in the study.

### Eligibility criteria for the GELATO trial

Eligible patients had metastatic or incurable locally advanced ILC. Non-female patients were also eligible for the trial. Sex of the patients was determined based on ID check upon hospital administration. The median age of the patients in the study was 60 years, ranging from 45 to 69 years. Patients were not preselected based on PD-L1 expression. Patients had to have a metastatic lesion or recurrence available for sequential biopsies (bone lesions were not allowed) and had to have evaluable disease according to RECISTv1.1 (ref. ^61^). In cases of ER^+^ disease, patients had to have progression after endocrine treatment in the advanced setting and had to have received anti-estrogen treatment and an aromatase inhibitor in the early stage or for the advanced setting. A maximum of two lines of palliative chemotherapy was allowed. Patients had to have a WHO performance status of 0 or 1 and normal bone marrow, kidney and liver functions, with a lactate dehydrogenase (LDH) below 500 U liter^–1^ (two times the upper limit of normal). Exclusion criteria were bone-only disease, symptomatic brain metastasis (stable and treated brain metastases were allowed), leptomeningeal disease localization, previous treatment with immune checkpoint inhibitors and/or a history of autoimmune disorders requiring immunosuppressive treatment. At the start of the trial, patients were eligible regardless of their receptor status. Because we aimed for a representative population for ILC with 10–20% ER^–^ patients^1,2^, inclusion of patients with TN-ILC (ER and PR expression of <10% and HER2^–^) was stopped after reaching 20% of total patients.

### Trial procedures

Clinically stable patients with disease progression according to RECISTv1.1 were permitted to continue anti-PD-L1 until confirmation of progression on a subsequent CT scan according to iRECIST guidelines^62^. Response evaluation was performed by a CT scan of the neck (if applicable), thorax and abdomen (including pelvis) at baseline (4 weeks before start) before the start of anti-PD-L1 and every 6 weeks during treatment (every 9 weeks after 24 weeks). RECISTv1.1 measurements were done by experienced breast cancer radiologists and, in case of inconsistencies, were revised by one dedicated radiologist. Carboplatin treatment was withheld in cases of hematological toxicity, such as anemia or neutropenia. Dose modification of atezolizumab was not allowed, but treatment interruptions were allowed in cases of toxicity or suspicion thereof. Adverse events were monitored every 3 weeks (weekly during carboplatin treatment) by laboratory assessments, vital signs and physical examinations. Grading of adverse events was done per National Cancer Institute’s Common Terminology Criteria for Adverse Events v4.03. Supportive treatment with antiemetics, bisphosphonates and palliative radiation (only if the response could still be evaluated) was allowed. Archival formalin-fixed paraffin-embedded (FFPE) blocks of primary tumors (biopsies in cases of neoadjuvant chemotherapy or resection material) were collected via PALGA (the nationwide network and registry of histopathology and cytopathology in the Netherlands)^63^. The study protocol is included in Supplementary Note.

### Trial objectives and endpoints

The primary endpoint of the trial was progression-free survival rate at 6 months (24 weeks), assessed from date of registration to date of progression according to RECISTv1.1 or death from any cause. Secondary endpoints were progression-free survival rate at 6 months in patients with immune-related ILC, progression-free survival rate at 12 months, progression-free survival according to iRECIST, overall survival, ORR and safety. The clinical benefit rate comprised CR, PR and SD for at least 24 weeks. Progression-free survival was calculated from date of registration to date of progression according to RECIST1.1 or date of death, whichever occurred earlier. Overall survival was calculated from date of registration to date of death or last date of follow-up. Patients were censored in cases of no event at the last assessment before the data cutoff of 1 October 2021. Duration of response was calculated from the first date of an objective response to date of progression according to RECIST1.1. Translational endpoints were the assessment of the immunogenic effects of carboplatin on the tumor microenvironment and in the circulation using IHC, next-generation sequencing, flow cytometry, the additive effect of anti-PD-L1 on these changes and exploration of predictive biomarkers.

### Statistics and reproducibility

A Simon’s two-stage^64^ design was used to determine the sample size. The median progression-free survival of palliative chemotherapy regimens in patients with endocrine treatment-refractory breast cancer typically lies within 2 to 4 months (Supplementary Note). If 25% of patients were free of progression at 6 months (24 weeks) in the GELATO trial, this would warrant further investigation of the treatment regimen. The null hypothesis that the true proportion of patients progression free at 6 months is 10% or lower was tested against a one-sided alternative of at least 25%. In the first stage of the trial, 22 patients had to be accrued. If two or fewer patients were progression free at 6 months, the study would be stopped, otherwise 18 additional patients could be included. This design yields a type I error rate of 0.04 and power of 0.80 when the true proportion of patients progression free at 6 months is 25%. The last 2 patients were registered in the same week and were therefore both included in the trial, leading to a total inclusion of 23 patients. Primary endpoint analysis for Simon’s two-stage was therefore performed separately for the first included 22 patients. Secondary and translational endpoint analyses were performed in the per protocol population (*n* = 23 ppatients who received at least one dose of anti-PD-L1; Extended Data Fig. 1). The data cutoff for follow-up was 1 October 2021. No data were excluded from the analyses. Data collection and analysis were not performed blind to the conditions of the experiments. The investigators were not blinded to the outcome assessment. Because the study included one experimental group, randomization and allocation procedures were not applicable. Further information on research design is available in the Nature Research Reporting Summary linked to this article.

### Flow cytometry fresh blood

**Extended Data Fig. 8:**
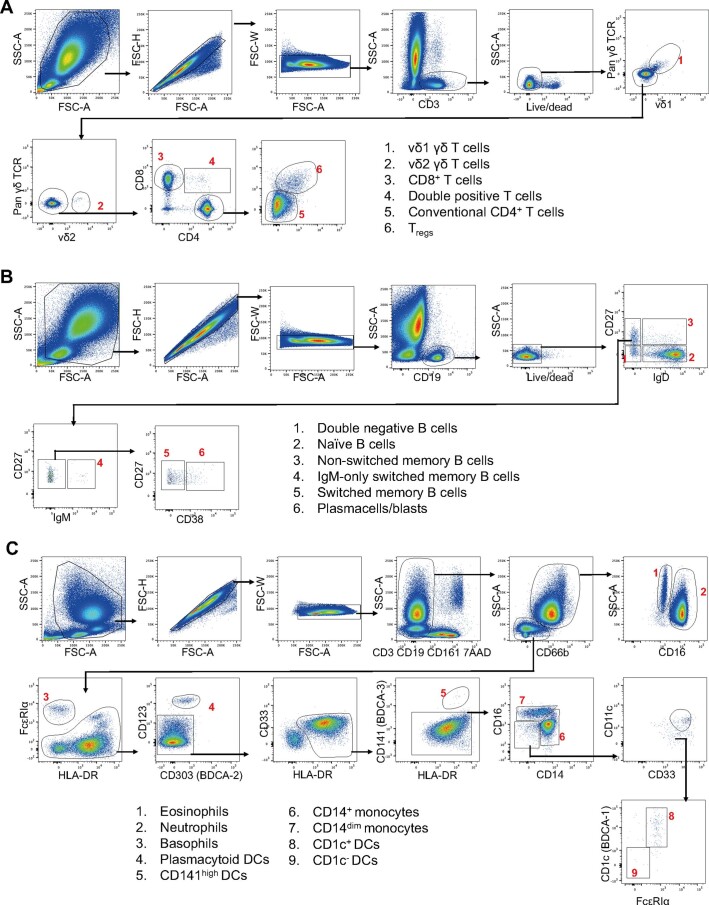
Gating strategies for flow cytometry analysis of peripheral blood immune populations. (**A**) T cell panel gating strategy identifying vδ1 γδ T cells (CD3+, vδ1+, pan γδ TCR+), vδ2 γδ T cells (CD3+, vδ2+), double positive T cells (CD3+, vδ1-, pan γδ TCR-, vδ2-, CD8+, CD4+), CD8 T cells (CD3+, vδ1-, pan γδ TCR-, vδ2-, CD8+, CD4-), conventional CD4 T cells (CD3+, vδ1-, pan γδ TCR-, vδ2-, CD8-, CD4+, FoxP3-) and Tregs (CD3+, vδ1-, pan γδ TCR-, vδ2-, CD8-, CD4+, FoxP3+, CD25high). (**B**) Gating strategy to identify B cell subsets identifying double negative B cells (CD19+, CD27-, IgD-), naïve B cells (CD19+, CD27-, IgD+), non-switched memory B cells (CD19+, CD27+, IgD+), IgM-only memory B cells (CD19+, CD27+, IgD-, IgM+), switched memory B cells (CD19+, CD27+, IgD-, IgM-, CD38-/+), and plasmacells/blasts (CD19+, CD27+, IgD-, IgM-, CD38high). (**C**) Myeloid panel gating strategy identifying eosinophils (lineage-, high side scatter, CD66b+ CD16−), neutrophils (lineage-, high side scatter, CD66b+ CD16+), basophils (lineage-, FcεRIα+, HLA-DR-), plasmacytoid DCs (lineage-, HLA-DR+, CD303+, CD123+), CD141high DCs (lineage-, HLA-DR+, CD33+, CD141+), CD14+ monocytes (lineage-, HLA-DR+, CD33+, CD14+, CD16-/+), CD14dim monocytes (lineage-, HLA-DR+, CD33+, CD14dim, CD16+), CD1c+ DCs (lineage-, HLA-DR+, CD33+, CD14-, CD16-, CD1c+, FcεRIα+) and CD1c- DCs (lineage-, HLA-DR+, CD33+, CD14-, CD16-, CD1c-, FcεRIα-).

Peripheral blood was collected in a K_2_EDTA vacutainer (BD) and processed within 24 h. Three panels spanning T cell, B cell and myeloid cell biology were used (see Supplementary Table 5 for all assessed immune cell populations, Supplementary Table 6 for antibodies and Extended Data Fig. 8 for the gating strategy), as described before^18^. Red blood cells were lysed (lysis buffer: distilled water, NH_4_Cl, NaHCCO_3_ and EDTA), and cells were resuspended in PBS containing 0.5% bovine serum albumin and 2 mM EDTA. For surface antigen staining, cells were incubated with human FcR blocking reagent (1:100; Miltenyi) for 15 min at 4 °C and then incubated with fluorochrome-conjugated antibodies for 30 min at 4 °C in the dark. For intracellular antigen staining, cells were fixed with 1× fixation/permeabilization solution (Foxp3/transcription factor staining buffer set, eBioscience) for 30 min at 4 °C and stained with fluorochrome-conjugated antibodies in 1× permeabilization buffer (eBioscience) for 30 min at room temperature. Viability was assessed by staining with either 7AAD staining solution (1:20; eBioscience) or a Zombie Red fixable viability kit (1:800; BioLegend). Data acquisition was performed on a BD LSRII flow cytometer using Diva software (BD Biosciences), and data analysis was performed using FlowJo software version 10.6.2. To obtain absolute white blood cell counts per milliliter of human blood, the total cell count after lysis was obtained using the NucleoCounter NC-200 (Chemometec) automated cell counter. To assess dynamics in each cell population with cell count per milliliter, linear modeling was performed using the limma R package v3.46.0 (https://kasperdanielhansen.github.io/genbioconductor/html/limma.html). Predicting log_2_-transformed cell counts per milliliter for the same patient at different time points was done by calculating log_2_ (cell counts per ml) ~ time point + patient ID. The modeling was performed independently for paired samples of baseline versus after induction (Fig. 4a) and baseline versus carboplatin + anti-PD-L1 (Fig. 4b). Sample pair dynamics were assessed analogously to the paired *t*-test. For visualization purposes, Benjamini–Hochberg corrected *P* values were plotted against the corresponding log_2_ (fold change) values, the log_2_ (fold change) from baseline to before atezolizumab treatment (log_2_ (before atezolizumab) – log_2_ (baseline)) and the log_2_ (fold change) from baseline to on atezolizumab (log_2_ (on atezolizumab) – log_2_ (baseline); Fig. 4a,b). The plots were made with the EnhancedVolcano R package v1.12.0 (https://github.com/kevinblighe/EnhancedVolcano).

### sTILs and IHC

FFPE tumor blocks of archived primary tumors and newly collected biopsies of metastatic lesions were used for sTIL assessment and CD8 and PD-L1 (SP142) IHC staining. IHC of FFPE tumor samples was performed on a BenchMark Ultra autostainer (Ventana Medical Systems). Briefly, paraffin sections were cut at 3 μm, heated at 75 °C for 28 min and deparaffinized in the instrument with EZ prep solution (Ventana Medical Systems). Heat-induced antigen retrieval was performed using Cell Conditioning 1 (Ventana Medical Systems) for 32 min at 95 °C (CD8) or 48 min at 95 °C (PD-L1). CD8 was detected using clone C8/144B (1:200 dilution, 32 min at 37 °C; Agilent/DAKO) and PD-L1 using clone SP142 (Ready-to-Use dispenser, 16 min at 37 °C; Roche/Ventana). Bound antibodies were detected using the OptiView DAB detection kit (Ventana Medical Systems). Slides were counterstained with hematoxylin and bluing reagent (Ventana Medical Systems). A PANNORAMIC 1000 scanner from 3DHISTECH was used to scan the slides at a magnification of ×40. Scans of all stainings were uploaded on SlideScore (www.slidescore.com). sTILs were assessed on a hematoxylin and eosin slide according to international standard from the International Immuno-Oncology Biomarker Working Group (www.tilsinbreastcancer.org)^65^. CD8 was assessed as percentage of positive cells in the tumor-associated stromal area, and PD-L1 was assessed as percentage of positive immune cells in the tumor and stromal area. Two expert pathologists (H.M.H. and R.S.) independently evaluated the stainings digitally, and the average of the scores was taken.

### DNA and RNA sequencing

DNA and RNA material was isolated from FFPE sections of primary tumors or FF tissue sections of biopsies of metastatic lesions containing at least 30% tumor cells. DNA and RNA isolation was done simultaneously using the Qiagen AllPrep DNA/RNA FF kit for FF tissue and the Qiagen AllPrep DNA/RNA FFPE kit for FFPE blocks, according to manufacturer’s instructions. Germline DNA was isolated from peripheral blood using the QIAsymphony DSP DNA midi kit. The total amount of DNA was quantified on a Nanodrop 2000 (Thermo Fisher). The amount of double-stranded DNA (dsDNA) in the genomic DNA samples was quantified using an Invitrogen Qubit dsDNA high-sensitivity assay kit. A maximum of 2,000 ng of dsDNA was fragmented by Covaris shearing. Samples were purified using 2× Agencourt AMPure XP PCR purification beads according to the manufacturer’s instructions. The sheared DNA samples were quantified and qualified on a BioAnalyzer system using the Agilent Technologies DNA7500 assay kit. With an input of a maximum of 1 μg of sheared DNA, library preparation for Illumina sequencing was performed using a KAPA HTP prep kit for FF DNA (KAPA Biosystems, KK8234) or a KAPA Hyper prep kit (KAPA Biosystems, KK8504) for FFPE DNA. Libraries were amplified with four (FF) or six (FFPE) PCR cycles and cleaned with 1× AMPure XP beads. Concentrations were measured with DNA7500 chips on a BioAnalyzer system. Six pools of six to seven samples were created using 500 ng of each indexed sample of FF DNA. Two pools of six to seven samples were created using 65 ng of each indexed sample of FFPE DNA. Two microliters of IDT TS-mix universal blockers and 5 μl of Invitrogen human Cot-1 DNA were added to each pool. Each pool was dried with a concentrator (Eppendorf). To each dried pool, 8.5 μl of hybridization buffer, 3.4 μl of hybridization component A (SeqCap hybridization and wash kit, Roche) and 1.1 μl of nuclease-free water were added to rehydrate the pool. Each pool was incubated at room temperature for 10 min, followed by an incubation at 96 °C for 10 min. Samples were hybridized with the IDT xGen exome research panel v1.0. The pool was captured and washed following the IDT protocol and amplified using 10 PCR cycles. The amplified pool was purified using AMPure XP beads (Beckman Coulter). The purified pools were quantified on an Agilent Bioanalyzer 7500 system, and one sequence pool was made by equimolar pooling. The sequence pool was diluted to a final concentration of 10 nM and subjected to sequencing on an Illumina NovaSeq 6000 machine with an SP2 300 cycle kit for a paired-end, 150-base pair run for FF samples and with an SP 200 cycle kit for a paired-end, 100-base pair run for FFPE samples, according to manufacturer’s instructions.

Quality and quantity of the total RNA were assessed using the 2100 Bioanalyzer and a Nano chip (Agilent). The percentage of RNA fragments with >200-nucleotide fragment distribution values (DV200) were determined using the region analysis method according to the manufacturer’s instructions (Illumina, technical-note-470-2014-001). Strand-specific libraries were generated using the TruSeq RNA exome library prep kit (Illumina) according to the manufacturer’s instructions (Illumina, 1000000039582v01). Briefly, total RNA was fragmented (only for FF material), random primed and reverse transcribed using SuperScript II reverse transcriptase (Invitrogen, 18064-014) with the addition of actinomycin D. Second-strand synthesis was performed using polymerase I and RNaseH with the replacement of dTTP for dUTP. The generated cDNA fragments were 3′-end adenylated and ligated to Illumina paired-end sequencing adapters and subsequently amplified by 15 cycles of PCR. The libraries were validated on a 2100 Bioanalyzer using a 7500 chip (Agilent) followed by a 1–4 plex library pooling containing up to 200 ng of each sample. The pooled libraries were enriched for target regions using the probe Coding Exome Oligos set (CEX, 45MB) according to the manufacturer’s instructions (Illumina, 1000000039582v01). Briefly, cDNA libraries and biotin-labeled capture probes were combined and hybridized using a denaturation step of 95 °C for 10 min and an incubation step from 94 °C to 58 °C with a ramp of 18 cycles, a 1-min incubation and 2 °C per cycle. The hybridized target regions were captured using streptavidin magnetic beads and subjected to two stringency washes, an elution step and a second round of enrichment followed by a cleanup using AMPure XP beads (Beckman, A63881) and PCR amplification of 10 cycles. The target enriched pools were analyzed on a 2100 Bioanalyzer using a 7500 chip (Agilent), diluted and subsequently pooled equimolar into a multiplex sequencing pool. The libraries were sequenced with 54 paired-end reads on a NovaSeq 6000 using an SP reagent kit v1.5 (100 cycles; Illumina).

### DNA-sequencing data analysis

DNA sequencing data were aligned to the GRCh38 reference genome with bwa aligner 0.7.17 (ref. ^66^) using the bwa-mem algorithm. Samtools fixmate 1.13 (https://github.com/samtools/) was used to correct mate information, and duplicate reads were marked with Picard MarkDuplicates. Next, base quality scores were recalibrated with GATK BaseRecalibrator^67^, and Mutect2 2.2 (ref. ^68^) was used to perform variant calling. The data that passed all Mutect2 filters were subsequently filtered with fings 1.7.1 (ref. ^69^), and vcf2maf 1.6.21 (https://github.com/mskcc/vcf2maf) was used to run VEP annotation of the variants and to produce a maf file. Variants with a variant allele frequency of >0.2 were included in the final analysis. TMB was calculated with the maftools 2.10.5 (ref. ^70^) tmb function. Variant allele frequency plots, mutational signature plots and oncoplots were created with maftools 2.10.5. Data were analyzed with Python 3.7.6 and R 4.1.1. Pandas 1.3.3 (https://pandas.pydata.org/) was used for data handling.

### RNA-sequencing data analysis

RNA-sequencing data were aligned to GRCh38 with STAR 2.7.1a, with the twopassMode option set to ‘Basic’^71^. Gene counts were obtained with the STAR quantMode option set to ‘GeneCounts’. Data quality was assessed with FastQC 0.11.5 (ref. ^72^), FastQ Screen 0.14.0 (ref. ^73^), the Picard CollectRnaSeqMetrics tool (https://broadinstitute.github.io/picard/) and RSeQC read_distribution.py and read_duplication.py tools 4.0.0 (ref. ^74^) and were found to be suitable for the downstream analysis. Data were subsequently normalized to transcripts per million. For cell deconvolution, CIBERSORTx was run in absolute mode with LM22 Source GEP, performing the batch correction^75^. Differential expression analysis was performed with DESeq2 1.34.0 (ref. ^76^). PAM50 classification was performed with the genefu R package 2.26.0 (ref. ^77^). The Gseapy 0.9.18 ssgsea tool^78^ with the sample_norm_method option set to ‘rank’ was used for gene set signature scoring. Data were analyzed with Python 3.7.6 and R 4.1.1. Pandas 1.3.38 (https://pandas.pydata.org/) and NumPy 1.18.1 (https://numpy.org/) were used for data handling. Seaborn 0.10.0 (https://seaborn.pydata.org/), Matplotlib 3.1.3 (https://matplotlib.org/) and statannotations 0.4.3 (https://github.com/trevismd/statannotations) were used for plotting.

### ^89^Zr-atezolizumab-PET/CT imaging

Based on previous work showing superior correlation of ^89^Zr-atezolizumab uptake on PET/CT with clinical response to atezolizumab compared to IHC- or RNA-sequencing-based predictive biomarkers^23^, an imaging biomarker side study was performed in the University Medical Center Groningen (NCT04222426). At baseline and after two cycles of carboplatin, a whole body ^89^Zr-atezolizumab-PET/CT was performed on a Biograph mcT 40 or 64-slice PET/CT (Siemens/CTI), as previously described^23^. Tumor lesions were identified on standard baseline FDG-PET/CT, with a minimum width of 10 mm. ^89^Zr-atezolizumab uptake was quantified in all lesions, with a maximum of 10 lesions per organ. Quantification of ^89^Zr-atezolizumab and FDG uptake was performed using the Accurate tool^79^ and Syngo.via imaging software VB20/30 (Siemens), respectively. A spherical volume of interest was drawn closely around all metastases. *SUV*_max_ values and background mean *SUV* (*SUV*_mean_) were calculated. TBRs were calculated by dividing the *SUV*_max_ by the thoracic aorta *SUV*_mean_. Change in tumor uptake between ^89^Zr-atezolizumab-PET at baseline and after two cycles of carboplatin was assessed as percent TBR change. In addition, we calculated the median and range of the ^89^Zr-atezolizumab uptake (TBR) and natural log-transformed ^89^Zr-atezolizumab uptake to obtain approximate normal distributions, yielding estimates of geometric means following back transformation of the results.

### Statistical analysis

Median time to event for progression-free survival, overall survival and duration of response was calculated with the Kaplan–Meier method. The impact of clinical benefit on overall survival was assessed with a 24-week landmark analysis in which only patients alive after 24 weeks were considered. Additionally, a time-dependent Cox analysis was performed, with clinical benefit (on the date of first PR or at 24 weeks in cases of SD) as a time-dependent variable. Frequencies, such as response rate and clinical benefit rate, were estimated with corresponding two-sided 95% CIs (Clopper–Pearson), and comparisons between frequencies were performed using a Fisher’s exact test. Two-sided non-parametric tests were used for translational analyses, a Mann–Whitney *U*-test for two independent groups and a Wilcoxon signed-rank test for paired data. The data met the assumptions of the statistical tests used. Data were analyzed with GraphPad Prism v9.0, IBM SPSS statistics 24, SAS v9.4, Python 3.7.6 and R 4.1.1. Reported *P* values are two sided and unadjusted unless stated otherwise.

### Reporting summary

Further information on research design is available in the Nature Portfolio Reporting Summary linked to this article.

## Supplementary information

Supplementary Information Study protocol.

Reporting Summary

Supplementary Table Supplementary Tables 1–6.

## Data Availability

DNA- and RNA-sequencing data are stored in the European Genome–Phenome Archive under the accession code EGAS00001006902. Sequencing data and source data supporting the findings of this study will be made available from the corresponding author upon reasonable request for academic use and within the limitations of the provided informed consent. Data requests will be reviewed by the corresponding author and Institutional Review Board of the NKI, and, after approval, applying researchers have to sign a data transfer agreement with the NKI.
