# Supplementary material for: PD-L1 blockade in combination with carboplatin as immune induction in metastatic lobular breast cancer: the GELATO trial
Source: Nat Cancer. 2023 Apr 10;4(4):535–49. doi: 10.1038/s43018-023-00542-x (PMC10132987; doi:10.1038/s43018-023-00542-x)
Supplement: Supplementary file 1 — Study protocol. [file 43018_2023_542_MOESM1_ESM.pdf]

nature cancer

Article

https://doi.org/10.1038/s43018-023-00542-x

PD-L1 blockade in combination with carboplatin as immune induction in metastatic lobular breast cancer: the GELATO trial

In the format provided by the authors and unedited

<u>AssessinG E</u>fficacy of carboplatin and <u>AT</u>ez<u>O</u>lizumab in metastatic <u>L</u>obular breast cancer: GELATO-trial

NKI study number: **M17GEL**
Eudract number: **2017-001428-23**
NL Number: **NL 61567.031.17**

Protocol committee: M Kok, C Blank, SC Linn

Co-investigators:

Genomics:	R Bernards (Agendia BV), LFA Wessels
Immunology:	T Schumacher, K de Visser
Quality control:	IAM Mandjes
Nurse practitioner:	I Kemper
Medical Oncology:	GS Sonke, M van Dongen
Pathology:	HM Horlings, K van de Vijver
Biometrics:	H van Tinteren
Microbiome:	M Nieuwdorp (AMC), N. de Clercq (AMC)

Local Principal Investigators:

UMCG:	CP Schröder
MUMC:	VCG Tjan-Heijnen
Erasmus MC:	A Jager, J Martens

Principal Investigator and study coordinator:
M Kok, MD PhD
Netherlands Cancer Institute, Division Medical Oncology, Plesmanlaan 121, 1066 CX, Amsterdam, The Netherlands, m.kok@nki.nl

Version 1.4 December 19th, 2017
Version 1.5 March 20th, 2019

PROTOCOL SIGNATURE SHEET

Name	Signature	Date
**Head of Department:**<br>***Dr. J. Stouthard, medical oncologist***		
**Coordinating Principal Investigator:**<br>***Dr. M. Kok, medical oncologist***		
**Participating institute:**<br><br>**Investigator:**		

Table of Contents

1 Synopsis 5

2 Background and rationale 10

3 Objectives and endpoints 12

4 Trial design 14

4.1 Trial design 14

4.2 Dose limiting toxicity 15

5 Patient selection criteria 17

6 Therapeutic regimens, dose adjustments and management of toxicity 19

6.1 Atezolizumab 19

6.1.1 Atezolizumab – Adverse Events 20

6.1.2 Atezolizumab – Immune-mediated Adverse Events 20

6.1.3 Atezolizumab – Safety in combination with chemotherapy 20

6.1.4 Atezolizumab – Administration 21

6.1.5 Atezolizumab - Duration of Therapy 21

6.2. Carboplatin 21

6.2.1 Carboplatin – Administration 21

6.3 Dose modification for toxicity 22

6.3.1 Atezolizumab dose modification and management of specific adverse events 22

6.3.2 Carboplatin dose modification 32

6.4 Co-medication 33

6.5 Pregnancy and breastfeeding 34

7 Clinical evaluation, laboratory tests, follow-up 35

7.1 Before treatment 35

7.1.1 Baseline screening 35

7.1.2 Before first carboplatin (induction day 1) 35

7.2 During treatment 35

7.3 At progression 37

7.4 Ongoing response or stable disease at 12 months (18 cycles) 37

7.5 Summary table 38

8 Response evaluation criteria 40

8.3.1 Measurability of tumor lesions at baseline 41

8.3.2 Tumor response evaluation 43

9 Statistical considerations 46

10 Translational research 48

11 Safety reporting 51

12 Administrative aspects and monitoring 55

13 Ethical considerations 57

Appendix A: NCI CTCAE 4.03 58

Appendix B: WHO Performance Status scale **59**
References **60**

1 Synopsis

**Rationale**	Immunotherapies, using blockade of the immune-inhibitory receptor PD-1 (or its ligand PD-L1), have created a revolution in the treatment of tumors such as melanoma, lung cancer, and bladder cancer [1-3]. In breast cancer patients, there is limited experience with T-cell checkpoint inhibitors to date. However, recently preliminary phase I data has shown significant responses in breast cancer patients [4-7]. Objective response rates range from 5 – 20%. Thus, although effective in a subset of patients, the majority of these breast cancer patients does not have benefit of single agent immunotherapy. The current challenges include i) development of predictive biomarkers for the selection of individual breast cancer patients who will benefit from immunotherapy and ii) to test combination immunotherapy strategies that integrate PD-L1-blockade with other immune-modulatory therapies to increase efficacy.<br><br>**Invasive lobular carcinoma (ILC)** is the second most frequent histological breast cancer subtype, accounting for around 10% of all breast cancers. The majority (around 95%[8]) of ILCs is estrogen receptor (ER) positive. Recently, in our institute, a large scale genomic analysis of ILC identified an **immune related (IR) subtype** within ILCs characterized by mRNA up-regulation of CD4, CD8, PD-L1, PD1 and CTLA4 and greater sensitivity to DNA-damaging agents. [9] In line with this, researchers from The Cancer Genome Atlas (TCGA) have shown that immune-related genes (interleukins, chemokines, MHC complex, IDO1 and IFNy) are highly expressed in a subset of ILC tumors. [10] Both IR and non-IR subtypes show similar clinical outcomes. [9] This molecular characterization may help select ILC patients for immunotherapy using atezolizumab in the future.<br><br>Since **classical anticancer agents can stimulate immune effector cells** [11, 12], we hypothesize that induction treatment with chemotherapy induces an anticancer immune response resulting in synergistic activity with anti-PD-L1. In this trial, we will use carboplatin as immune response induction treatment. Carboplatin not only stimulates class I HLA expression but also inhibits signal transducer and activator of transcription 6 (STAT6)-regulated expression of programmed death ligand 2 (PD-L2), thus limiting immunosuppression by both DCs and tumor cells. [13] And work of the Melief group showed that low dose platinum and vaccination act synergistically in tumor eradication. [14] In addition, preliminary work in a mouse model for ILC [15] performed by the de Visser lab at our institute suggests that the **activity of T-cell check point inhibitors is enhanced using platinum agents** (work in progress). Moreover, the fact that cell lines of the IR subtype show greater sensitivity to DNA-damaging agents [9] provides additional evidence to combine PD-L1-blockade with carboplatin in this GELATO-trial.
**Methodology**	This is a single arm multicenter non-randomized phase II trial testing the efficacy of the combination of carboplatin plus atezolizumab in metastatic ILC
**Primary objective**	Efficacy of atezolizumab together with carboplatin in metastatic ILC
**Primary endpoint**	Proportion of patients who remain free of progression (time from start carboplatin to tumor progression or death from any cause) at 6 months. Progression as defined by RECIST 1.1 [16] will be used.
**Secondary objectives**	1) Proportion of patients free of progression at 6 months in the IR-profile subgroup vs the non-IR-subgroup as defined by gene expression profiling (retrospectively defined as previously described [9])<br>2) Safety<br>3) Efficacy of atezolizumab plus carboplatin using objective response rate<br>4) Efficacy of atezolizumab plus carboplatin using proportion of patients free of

	progression at 12 months<br>5) Efficacy of atezolizumab plus carboplatin using overall survival<br>6) Explore the role of potential biomarkers to predict response to atezolizumab plus carboplatin
**Secondary endpoints**	1) Proportion of patients free of progression (RECIST 1.1[16]) at 6 months in the IR-profile subgroup vs the non-IR-subgroup as defined by gene expression profiling (retrospectively defined as previously described [9])<br>2) Progression as defined by iRECIST [17]<br>3) Percentage of patients with toxicity (according to CTCAE v4.0.3, Appendix A) and immune-related toxicity defined as the Adverse Events of Special Interest (AESI's) for atezolizumab<br>4) Objective response rate (RECIST 1.1[16])<br>5) Proportion of patients who remain free of progression at 12 months. Progression as defined by RECIST 1.1 [16] will be used.<br>6) Overall survival
**Translational studies**	• Analyze the increase in immunogenicity after immune response induction treatment using carboplatin using pre-treatment biopsies/blood samples and biopsies/blood samples taken after 2 cycles of carboplatin before the start of atezolizumab. Potential markers to assess immunogenicity: TILs, PD-L1, CD8, FOXP3, CD68 (IHC), changes in gene expression with an emphasize on IFNγ-related gene signatures [18, 19], serum cytokine levels, proportion of effector T cells and myeloid subpopulations in peripheral blood [20] (in collaboration with Schumacher and the Visser labs)<br>• Comprehensive analyses of responding and non-responding cases using potential predictive markers such as but not limited to TILs, PD-L1 (according to the algorithm of Roche diagnostics), CD8, CD68, number of neoantigens, IFNγ-related gene signatures [18, 21] and serum LDH<br>• Biobanking of tumor tissues (frozen and FFPE), serum, PMBCs, platelets, circulating tumor DNA (ctDNA), cell-free DNA (cfDNA) and stool samples. All collected at baseline, after induction/before atezolizumab cyle 1 and before cycle 3, 5 and 7 of atezolizumab for the purpose of retrospective analyses of candidate biomarkers or experiments to gain insight into response to immunotherapy in ILCs.
**Study design**	This Dutch multicenter investigator-initiated study is a single-arm phase II trial. After a biopsy from a metastatic lesion, patients can enter the trial and will start with the immune response induction treatment using carboplatin in a weekly schedule. After two weeks, a second biopsy will be taken and atezolizumab will be co-administered in a 3-week-schedule. A Simon's two-stage (minimax) design will be used (see 'Statistics' section below for sample size calculation of the two stages).[22] Although the evaluation of potential predictive gene profiles such as the IR-profile is part of this trial (see secondary and translational endpoints) patients will not be selected for the trial based on a certain gene profile.

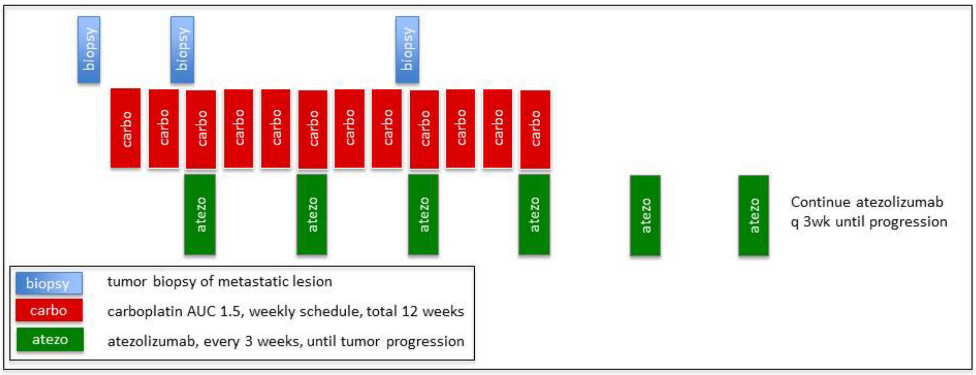

**Treatment**	Carboplatin AUC 1.5 [23], intravenous administration, weekly schedule, maximum 12 administrations (no steroids necessary). After two administrations of carboplatin, atezolizumab (1200 mg flat dose) will be given in a 3-weekly schedule until tumor progression. After 12 months of atezolizumab treatment discontinuation is allowed in case of ongoing response or stable disease. At signs of progression after discontinuation of the treatment, atezolizumab can be re-started. Carboplatin will be given for 12 weeks. If carboplatin has to be discontinued due to toxicity, atezolizumab can be continued as monotherapy.
**Special safety assessments**	Until now, atezolizumab has not been administered with carboplatin AUC 1.5. Though in advanced NSCLC, no DLTs were observed in patients treated with nivolumab (anti-PD1) plus carboplatin/paclitaxel [24]. The safety and efficacy of atezolizumab in combination with carboplatin AUC6 (and nab-paclitaxel) is currently being investigated (NCT01633970) in metastatic breast cancer patients. Currently, at the NKI in a phase I study (N16LOG) the safety of carboplatin (AUC5)/cyclophosphamide/atezolizumab is assessed. Pending these results, special safety assessments will be performed on the first 6 (3+3) patients entering this GELATO trial (phase Ib, run in). Based on the well-known toxicity profiles of both carboplatin and atezolizumab, we don't expect significant toxicity using the combination treatment.<br>In order to monitor unexpected toxicity related to the use of atezolizumab in combination with carboplatin AUC1.5, dose-limiting toxicities (DLTs) will be scored during the first 5 weeks of treatment (DLT window). DLT is defined as grade 4 or 5 toxicity according to CTCAE 4.03, or grade 3 persisting for 6 weeks (see section 4 'Trial design' in protocol). In case in the first 3 patients 1 DLT is observed, another 3 patients will be included. In case in this group a second DLT is seen (2/6), the investigators will report this to Roche as well as to the Institutional Review Board (IRB). Together with Roche and the IRB the protocol committee will discuss whether patients can still be entering the trial or whether the trial needs to be re-designed with regard to dose or inclusion criteria.
**Number of patients**	In the run-in phase Ib part, six patients will be included (3+3). In the first stage 22 (including the first n=6 from the run-in phase Ib part) patients will be included. If 3 or more patients are free of progression at 6 months (see statistics section below), an additional 18 subjects will be entered. Total= 22+18=40. For evaluation of efficacy according to the Simon's two-stage design we will use all patients who received at least 1 cycle of atezolizumab (per protocol population).
**Randomization**	Not applicable
**Statistics**	For metastatic ILC patients who are refractory to endocrine treatment, no first or second line 'standard' therapies have been defined. Frequently used anticancer agents are capecitabine, or a taxane. The median PFS reached with these therapies typically lies between 2-4 months, but limited data are available. [25, 26] For this study, it is determined that a regimen that is likely to yield a proportion of patients that is free of progression at 6

	months of more than 25% should be further explored in a randomized study.<br>Simon's two-stage (minimax) design will be used.[22] The null hypothesis that the true proportion of patients that is free of progression at 6 months is <= 10% will be tested against a one-sided alternative of at least 25%. In the first stage, 22 patients will be accrued (all must have received at least one cycle of atezolizumab). If there are 2 or fewer patients free of progression at 6 months in these 22 patients, the study will be stopped. Otherwise, 18 additional patients will be accrued for a total of 40. The null hypothesis will be rejected if 8 or more patients out of those 40 are free of progression at 24 weeks. This design yields a type I error rate 0.04 and power of 80% when the true proportion of patients free of progression at 24 weeks is 25%.
**Patient selection criteria**	♦ Signed and written informed consent<br>♦ Age 18 year or older<br>♦ Metastatic or incurable locally advanced lobular breast cancer with **confirmation of the lobular histology and E-cadherin loss or aberrant staining (IHC) on a biopsy of a metastatic lesion.** For patients with a tumor with a mixed-type IDC/ILC or IDC with lobular features a loss or aberrant expression of E-cadherin (IHC) is required [27]<br>♦ Estrogen receptor expression of at least 10% on a metastatic lesion (independent of progesterone receptor expression and HER2 expression)<br>♦ **Metastatic lesion accessible** for histological biopsies (Mandatory biopsies: pre-induction treatment with carboplatin, before start atezolizumab, after 2 cycles of atezolizumab. Optional: upon development of acquired resistance). Biopsies from bone lesions are not permitted. Interval between **last chemotherapy and pre-induction biopsy has to be at least 14 days** (not for endocrine treatment in view of the relatively long half-life). The pre-induction treatment biopsy has to contain **sufficient tumor content (≥100 tumor cells)**; subjects with samples that have insufficient tumor content will require re-biopsy prior to start carboplatin.<br>♦ Evidence of **progression of disease** (either radiological or clinical)<br>♦ Disease progression had to occur after previous (not necessarily the most recent) endocrine therapy in the advanced setting. Patients with an ER-positive tumor must have received **an anti-estrogen (tamoxifen and/or fulvestrant) and at least one aromatase inhibitor** for early breast cancer or metastatic disease<br>♦ **A maximum of two lines of palliative chemotherapy** for metastatic or incurable locally advanced breast cancer is allowed. HER2-targeting therapy is allowed for the HER2-positive cases. Carboplatin pretreatment is allowed, as long as no progression was observed and the last dose was administered 6 months before starting study treatment.<br>♦ WHO performance status of 0 or 1<br>♦ Absolute lymphocyte count ≥ 0.5 x 10^9/L, ANC ≥ 1.5 x 10^9/L, platelets ≥150 x 10^9/L, Hemoglobin ≥ 6.0 mmol/L<br>♦ Bilirubin < 1.5 x upper limit of the normal range (ULN), except subjects with Gilbert Syndrome; alkaline phosphatase < 2.5 x ULN (< 5 x ULN in case of liver metastases, and < 7 x ULN in case of bone metastases); transaminases (ASAT/ALAT) < 3 x ULN (and < 5 x ULN in case of liver metastases), **LDH < 2xULN**.<br>♦ Serum creatinine ≤ 1.5 ULN or calculated (Cockcroft-Gault) or measured creatinine clearance > 40 mL/min<br>♦ Evaluable disease or measurable according to RECIST 1.1<br>♦ Subjects with brain metastases are eligible if they have been treated, are not

	symptomatic and there is no magnetic resonance imaging (MRI) evidence of progression for at least 28 days prior to registration in the study. There must also be no requirement for immunosuppressive doses of systemic corticosteroids (> 10 mg/day prednisone equivalents) for at least 2 weeks prior to study drug administration<br>♦ No known leptomeningeal disease localization<br>♦ No history of having received other anticancer therapies within 2 weeks of start of the study drug<br>♦ No history of immunodeficiency, autoimmune disease, conditions requiring immunosuppression (>10 mg/day prednisone equivalents) or chronic infections. Subjects with vitiligo, diabetes mellitus type I, psoriasis not requiring systemic treatment or resolved childhood asthma/atopy would be an exception to this rule. Subjects that require intermittent use of bronchodilators, inhaled steroids, or local steroid injections would not be excluded from the study. Subjects with hypothyroidism stable on hormone replacement, Sjøgren's syndrome or conditions not expected to recur in the absence of an external trigger will not be excluded from the study. Adrenal replacement doses >10 mg/day prednisone or equivalents are permitted in the absence of active autoimmune disease<br>♦ No prior treatment with immune checkpoint blockade such as but not limited to anti-PD(L)1, anti-PD-L2, anti-CTLA-4, anti-GITR or CD137/OX40-agonist<br>♦ No live vaccine within 2 weeks prior to start of study, at any time during the study, or within 5 months following the last dose of atezolizumab. Inactivated vaccines are allowed<br>♦ No active other cancer<br>♦ No active hepatitis B (defined as having a positive hepatitis B surface antigen [HbsAg] test at screening) or active hepatitis C. Patients with past hepatitis B virus (HBV) infection or resolved HBV infection (defined as having a negative HBsAg test and a positive antibody to hepatitis B core antigen [anti-HBc] antibody test) are eligible. Patients positive for hepatitis C virus (HCV) antibody are eligible only if polymerase chain reaction (PCR) is negative for HCV RNActive tuberculosis<br>♦ No history of uncontrolled serious medical or psychiatric illness<br>♦ No known hypersensitivity to Chinese hamster ovary cell products or other recombinant human antibodies<br>♦ Absence of any psychological, familial, sociological or geographical condition potentially hampering compliance with the study protocol and follow-up schedule<br>♦ No current pregnancy or breastfeeding. Women of childbearing potential (WOCBP) must use adequate contraceptive protection

2 Background and rationale

ILC

Invasive lobular breast cancer (ILC) is the most frequent special histological subtype of breast cancer (5-15%) after invasive breast carcinoma of no special type (NST) previously known as invasive ductal breast cancer (IDC).[27] ILC is typically characterized by non-cohesive cells individually dispersed or arranged in a single-file linear pattern in fibrous stroma. ILCs mostly express the estrogen receptor (ER), lack HER2 and lost the cell adhesion molecule E-cadherin. Several special ILC subtype have been described on the basis of architecture (solid, alveolar), cytonuclear characteristics (pleomorphic, tubulolobular) and a mixed non-classical is composed of cases showing an admixture of the classic type with one or more of these variant patterns. [28] The classic ILC and mixed variants contribute to the majority of ILCs comprising up to 75% of all cases. In addition, both invasive carcinoma NST and lobular features of differentiation are present in about 5% of invasive breast cancers.

ILC has a distinctive biology and clinical behavior compared with IDC. For example, the prognostic impact of histology (ILC vs IDC) appears to vary with time. This was shown in a pooled analysis of over 9000 patients with extended follow-up that reported a 16 percent lower risk of recurrence for ILC compared with IDC during the first six years of follow-up; however, ILC conferred a 54 percent higher risk of relapse after six years. [29] For metastatic ILC patients who are refractory to endocrine treatment, no first or second line 'standard' therapies have been defined. Frequently used anticancer agents are capecitabine, or a taxane. The median progression-free survival (PFS) reached with these therapies typically lies between 2-4 months only, but limited data are available. [25, 26] In conclusion, there is a clear clinical need for new treatment options for patients with metastatic ILC.

Immune checkpoint inhibitors in breast cancer

No data are available regarding response to checkpoint blockade in ILCs. However, preliminary data of early immunotherapy trials in ER-positive breast cancer show a modest response rate to anti-PD(L)1 monotherapy: 2.8% (n=72, including 6 ILCs) with anti-PD-L1 monotherapy in ER-positive breast cancer not selected for PD-L1 positivity. [6] The response rate increases to 12% (n=25, number of ILCs unknown) in the PD-L1 positive ER-positive breast cancers [7] with a clinical benefit rate (defined as objective response or stable disease for over 24 weeks) of 20%. Although the sample size is small and the patients were heavily pretreated, the response rate in ER-positive breast cancer seems lower compared to other breast cancer subtypes such as triple negative breast cancer (TNBC) with response rates between 10-20%. [6, 30-32] This illustrates the need for the discovery of predictive biomarkers that can aid in selecting breast cancer patients for immunotherapy. Moreover, the challenge lies ahead of us to combine anti-PD(L)1 with novel immune-modulatory drugs or conventional anti-cancer treatments that are able to increase the immunogenicity of breast tumors.

Gene expression: immune-related ILC subtype

Besides its tendency to relapse later in the disease course, ILCs are more likely to metastasize to the bone, peritoneum, gastrointestinal tract and ovaries suggesting a different underlying biology.[33] Recently, three independent groups presented a comprehensive molecular characterization of ILCs. [8-10] Investigators from our institute performed a large-scale genomic analysis of ILCs and identified an immune related (IR) subtype within ILCs characterized by mRNA up-regulation of CD4, CD8, PD-L1, PD1 and CTLA4 and greater sensitivity to DNA-damaging agents. [9] Tumor with this immune-related gene signature show significantly

more lymphocytic infiltration. In line with this, researchers from The Cancer Genome Atlas (TCGA) have shown that immune-related genes (interleukins, chemokines, MHC complex, IDO1 and IFNγ) are highly expressed in a subset of ILC tumors. [10] This molecular characterization may help select ILC patients for immunotherapy in the future.

Exploiting the immune-modulatory effect of chemotherapy

Since classical anticancer agents can stimulate immune effector cells [11, 12], we hypothesize that induction treatment with chemotherapy induces an anticancer immune response resulting in synergistic activity with anti-PD-L1. Another benefit of adding chemotherapy to immunotherapy could be that the cytostatic effect of chemotherapy provides relatively rapid tumor control in the window of several weeks before the response to anti-PD(L)1 occurs. The first data on chemotherapy plus anti-PD-L1 show that the response rates are indeed higher when anti-PD-L1 is administered in combination with chemotherapy. [30]
In this trial, we will use carboplatin as immune response induction treatment. Carboplatin not only stimulates class I HLA expression but also inhibit signal transducer and activator of transcription 6 (STAT6)-regulated expression of programmed death ligand 2 (PD-L2), thus limiting immunosuppression by both dendritic cells (DCs) and tumor cells. [13] And work of the Melief group showed that low dose platinum and vaccination act synergistically in tumor eradication. [14] In addition, preliminary work in a mouse model for ILC [15] performed by the de Visser lab at our institute suggests that the activity of T-cell check point inhibitors is enhanced using platinum agents (work in progress). Moreover, the fact that cell lines of the IR subtype show greater sensitivity to DNA-damaging agents [9] provides additional evidence to combine PD-1-blockade with carboplatin in this GELATO-trial.

Rationale

Eventually all patients with metastatic ILC will become resistant to endocrine treatment and no targeted therapies are currently available for this subgroup. Recently, two independent research consortia have identified a subgroup of ILC that harbor upregulation of immune-related genes including IFNγ, type I IFNs, MHC and chemokines as well as PD-1, PD-L1 and CTLA-4. This so called immune-related (IR) subtype of ILC shows a greater sensitivity to DNA-damaging agents in vitro and in a mouse model for ILC a synergy between checkpoint-blockade and platinum is observed. Therefore, we hypothesize that anti-PD-L1 in combination with platinum could be effective in ILC. More specific, we will explore whether this effect is more pronounced in the subgroup of ILC with an IR signature.

3 Objectives and endpoints

3.1 Primary Objective

To assess the efficacy of atezolizumab in combination with carboplatin in metastatic ILC

3.2 Secondary Objective

- To evaluate the outcome after atezolizumab together with carboplatin in the IR-profile subgroup vs the non-IR-subgroup as defined by gene expression profiling (retrospectively defined as previously described [9])
- To evaluate the safety of atezolizumab together with carboplatin
- To evaluate the efficacy of atezolizumab plus carboplatin using objective response rate
- To evaluate the efficacy of atezolizumab plus carboplatin using proportion of patients free of progression at 12 months
- To evaluate the efficacy of atezolizumab plus carboplatin using overall survival
- To explore the role of potential biomarkers to predict response to atezolizumab plus carboplatin

3.3 Translational studies

- Analyze the increase in immunogenicity after immune response induction treatment using carboplatin using pre-treatment biopsies/blood samples and biopsies/blood samples taken after 2 cycles of carboplatin before the start of atezolizumab. Potential markers to assess immunogenicity: TILs, PD-L1, CD8, FOXP3, CD68 (IHC), changes in gene expression with an emphasize on IFNγ-related gene signatures [18, 19], serum cytokine levels, proportion of effector T cells and myeloid subpopulations in peripheral blood [20] (in collaboration with Schumacher and the Visser labs)
- Comprehensive analyses of responding and non-responding cases using potential predictive markers such as but not limited to TILs, PD-L1 (according to the algorithm of Roche diagnostics), CD8, CD68, number of neoantigens, IFNγ-related gene signatures and serum LDH
- Biobanking of tumor tissues (frozen and FFPE), serum, plasma, whole blood and stool samples. All collected at baseline, after induction/before start atezolizumab (cycle 1), before third, fifth and seventh cycle of atezolizumab (cycle 3, 5 and 7) for the purpose of retrospective analyses of candidate biomarkers or experiments to gain insight into response to immunotherapy in ILCs.

3.4 Endpoints

The primary endpoint is:
Proportion of patients who remain free of progression at 6 months. Progression as defined by RECIST 1.1 [34] will be used.

The secondary endpoints are:

- Proportion of patients free of progression (RECIST 1.1[34]) at 6 months in the IR-profile subgroup vs the non-IR-subgroup as defined by gene expression profiling(retrospectively defined as previously described [9])
- Proportion of patients who remain free of progression at 12 months. Progression as defined by RECIST 1.1 [34] will be used.
- Progression as defined by iRECIST [17]
- Overall survival

- Percentage of patients with toxicity (according to CTCAE v4.03, Appendix A) and immune-related toxicity defined as the Adverse Events of Special Interest (AESI's) for atezolizumab (see section 6 and Investigator's Brochure for atezolizumab)
- Objective response rate (RECIST 1.1[34])

  The exploratory endpoints are listed in Section 10 Translational Research

4 Trial design

4.1 Trial design

This Dutch multicenter investigator-initiated study is a single-arm phase II trial including a run-in phase I part. The design is presented in Figure 1. After a biopsy from a metastatic lesion, patients can enter the trial and will start with the immune response induction treatment using carboplatin in a weekly schedule. After two weeks a second biopsy will be taken and atezolizumab will be co-administered in a 3-week-schedule.

A Simon's two-stage (minimax) design will be used (see 'Statistics' section for sample size calculation of the two stages).[22] After 22 patients (stage I, including the first 6 patients in the DLT window, see section below) the proportion of patients free of progression will be determined. If there are 2 or fewer patients free of progression at 6 months in these 22 patients, the study will be stopped. For evaluation of efficacy according to the Simon's two-stage design we will use all patients who received at least 1 cycle of atezolizumab (per protocol population).

Although the evaluation of potential predictive gene profiles such as the IR-profile is part of this trial (see secondary and translational endpoints) patients will not be selected for the trial based on a certain gene profile.

Figure 1

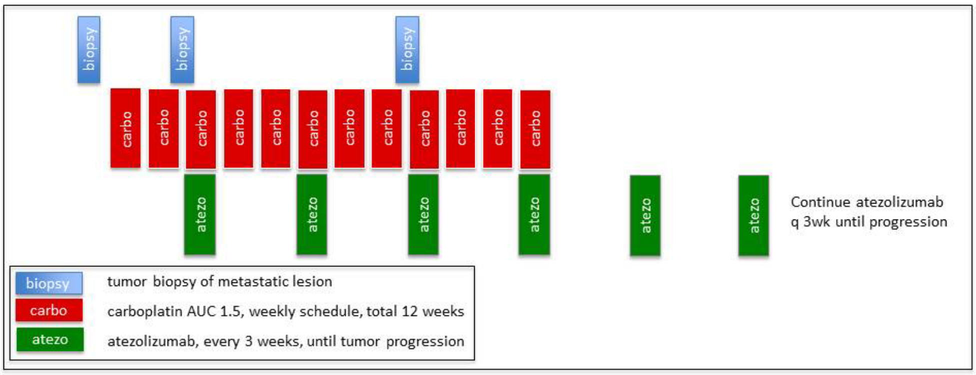

Treatment
Carboplatin AUC 1.5 [17], intravenous administration, weekly schedule, maximum 12 administrations (no steroids necessary). After two administrations of carboplatin, atezolizumab (1200 mg flat dose) will be given in a 3-weekly schedule until tumor progression. Carboplatin will be administered before the atezolizumab infusion.
Carboplatin will be given for 12 weeks. If carboplatin has to be discontinued due to toxicity, atezolizumab can be continued as monotherapy.
Atezolizumab will be given until tumor progression or intolerable toxicity. After 12 months of atezolizumab treatment discontinuation is allowed in case of ongoing response or stable disease. At signs of progression after discontinuation of the treatment, atezolizumab can be re-started.

Number of patients

The run-in phase Ib part (see section below) will included 6(3+3) patients. These 6 patients will also be part of stage I of the trial (n=22). In case 3 or more patients are free of progression at 6 months, an additional 18 patients will enter the trial. A total of 40 patients will participate (n=40).

4.2 Dose limiting toxicity

Until now, atezolizumab has not been administered with carboplatin AUC1.5. Though in advanced NSCLC, no DLTs were observed in patients treated with nivolumab (anti-PD1) plus carboplatin/paclitaxel [24]. The safety and efficacy of atezolizumab in combination with carboplatin AUC6 (and nab-paclitaxel) is currently being investigated (NCT01633970) in metastatic breast cancer patients. Currently, at the NKI in a phase I study (N16LOG) the safety of carboplatin (AUC5)/cyclophosphamide/atezolizumab is assessed. Pending these results, special safety assessments will be performed on the first 6 (3+3) patients entering this GELATO trial (phase I, run in). Based on the well-known toxicity profiles of both carboplatin and atezolizumab, we don't expect significant toxicity using the combination treatment.

In order to monitor unexpected toxicity related to the use of atezolizumab in combination with carboplatin AUC1.5, dose-limiting toxicities (DLTs) will be scored during the first 5 weeks of treatment (DLT window). In case in the first 3 patients 1 DLT is observed, another 3 patients will be included. In case in this group a second DLT is seen (2/6), the investigators will report this to Roche as well as to the Institutional Review Board (IRB). Together with Roche and the IRB the protocol committee will discuss whether patients can still be entering the trial or whether the trial needs to be re-designed with regard to dose or inclusion criteria.

The following adverse events are considered DLTs:

DLT is defined as grade 4 or 5 toxicity according to CTCAE 4.03, or grade 3 persisting for 6 weeks and these toxicities cannot be attributed to progression of disease

Hematological toxicities defined as:

- Grade 4 neutropenia (ANC <0.5 x 109/l) lasting ≥ 14 days
- Grade ≥ 3 febrile neutropenia
- Grade 4 thrombocytopenia lasting ≥ 48 hours
- Grade ≥3 thrombocytopenia associated with clinical significant bleeding episodes

Non-hematological toxicity ≥ 3 including:

- Grade 3 hyperbilirubinemia lasting for > 48 hours or any Grade 4 hyperbilirubinemia
- Grade ≥ 3 AST/ALT elevations with hyperbilirubinemia of ≥ grade 2
- Grade 4 AST/ALT elevations
- For patients with Grade 2 AST/ALT, and/or alkaline phosphatase abnormality at baseline, an increase to ≥ 10 x the upper limit of normal (ULN) that does not resolve to Grade ≤ 2 within 48 hours (if symptomatic) or that does not resolve to Grade ≤ 1 within 3 weeks of onset (if asymptomatic) will be considered a DLT after AST/ALT elevation due to disease progression has been ruled out
- Grade ≥ 3 pneumonitis
- Grade ≥ 3 colitis

Failure to recover from any toxicity that results in a delay of 2 scheduled administrations of atezolizumab is considered a DLT. The following are not considered DLTs:

- Grade ≥ 3 nausea, vomiting, or diarrhea that resolves to Grade ≤ 1 with or without treatment prior to the next infusion

- Grade ≥ 3 immune-related adverse event that resolves to Grade ≤ 1 with immunosuppressant therapy within 3 weeks of its onset

- Grade ≥ 3 fatigue that resolves to Grade ≤ 2 within 1 week

- Grade ≥ 3 arthralgia that can be adequately managed with supportive care or that resolves to Grade ≤ 2 within 1 week

- Grade ≥ 3 hypophosphatemia reversible to < Grade 2 within 1 week

- Grade ≥ 3 laboratory abnormality that is asymptomatic and deemed by the investigator not to be clinically significant

- Grade ≥ 3 autoimmune thyroiditis or other endocrine abnormality that can be managed by endocrine therapy or hormonal replacement

- Grade ≥ 3 tumor flare defined as local pain, irritation, or rash localized at sites of known or suspected tumor

- Alopecia (any grade)

- Infusion related reactions (IRRs): IRRs are not considered to be DLTs because based on experience with monoclonal antibodies; IRRs are not dose-related events. Precautions will be taken if IRRs Grade ≥ 2 occurs. If described precautions are not sufficient, other options will be discussed between the sponsor and the investigator.

5 Patient selection criteria

- Signed and written informed consent
- Age 18 year or older
- Metastatic or incurable locally advanced lobular breast cancer with **confirmation of the lobular histology and E-cadherin loss or aberrant staining (IHC) on a biopsy of a metastatic lesion.** For patients with a tumor with a mixed-type IDC/ILC or IDC with lobular features a loss or aberrant expression of E-cadherin (IHC) is required [27]
- Estrogen receptor expression of at least 10% on a metastatic lesion (independent of progesterone receptor expression and HER2 expression)
- **Metastatic lesion accessible** for histological biopsies (Mandatory biopsies: pre-induction treatment with carboplatin, before start atezolizumab, after 2 cycles of atezolizumab. Optional: upon development of acquired resistance). Biopsies from bone lesions are not permitted. Interval between **last chemotherapy and pre-induction biopsy has to be at least 14 days** (not for endocrine treatment in view of the relatively long half-life). The pre-induction treatment biopsy has to contain **sufficient tumor content (≥100 tumor cells**); subjects with samples that have insufficient tumor content will require re-biopsy prior to start carboplatin.
- Evidence of **progression of disease** (either radiological or clinical)
- Disease progression had to occur after previous endocrine (not necessarily the most recent) therapy in the advanced setting. Patients with an ER-positive tumor must have received **an anti-estrogen (tamoxifen and/or fulvestrant) and at least one aromatase inhibitor** for early breast cancer or metastatic disease.
- **A maximum of two lines of palliative chemotherapy** for metastatic or incurable locally advanced breast cancer is allowed. HER2-targeting therapy is allowed for the HER2-positive cases. Carboplatin pretreatment is allowed, as long as no progression was observed and the last dose was administered 6 months before starting study treatment.
- WHO performance status of 0 or 1
- Absolute lymphocyte count ≥ 0.5 x 10^9/L, ANC ≥ 1.5 x 10^9/L, platelets ≥150 x 10^9/L, Hemoglobin ≥ 6.0 mmol/L
- Bilirubin < 1.5 x upper limit of the normal range (ULN), except subjects with Gilbert Syndrome; alkaline phosphatase < 2.5 x ULN (< 5 x ULN in case of liver metastases, and < 7 x ULN in case of bone metastases); transaminases (ASAT/ALAT) < 3 x ULN (and < 5 x ULN in case of liver metastases), **LDH < 2xULN**.
- Serum creatinine ≤ 1.5 ULN or calculated (Cockcroft-Gault) or measured creatinine clearance > 40 mL/min
- Evaluable disease or measurable according to RECIST 1.1
- Subjects with brain metastases are eligible if they have been treated, are not symptomatic and there is no magnetic resonance imaging (MRI) evidence of progression for at least 28 days prior to first dose of study drug administration. There must also be no requirement for immunosuppressive doses of systemic corticosteroids (> 10 mg/day prednisone equivalents) for at least 2 weeks prior to study drug administration
- No known leptomeningeal disease localization
- No history of having received other anticancer therapies within 2 weeks of start of the study drug
- No history of immunodeficiency, autoimmune disease, conditions requiring immunosuppression (>10 mg/day prednisone or equivalent) or chronic infections. Subjects with vitiligo, diabetes mellitus type I, psoriasis not requiring systemic treatment or resolved childhood asthma/atopy would be an exception to this rule. Subjects that require intermittent use of bronchodilators, inhaled steroids, or local steroid injections would not be excluded from the study. Subjects with hypothyroidism stable on hormone replacement, Sjøgren's syndrome or conditions not expected to recur in the absence of an external trigger will not be excluded from the study. Adrenal replacement doses >10 mg/day prednisone or equivalent are permitted in the absence of active autoimmune disease
- No prior treatment with immune checkpoint blockade such as but not limited to anti-PD(L)1, anti-PD-L2, anti-CTLA-4, anti-GITR or CD137/OX40-agonist

- No live vaccine within 2 weeks prior to start of study, at any time during the study, or within 5 months following the last dose of atezolizumab. Inactivated vaccines are allowed
- No active other cancer
- No active hepatitis B (defined as having a positive hepatitis B surface antigen [HbsAg] test at screening) or active hepatitis C. Patients with past hepatitis B virus (HBV) infection or resolved HBV infection (defined as having a negative HBsAg test and a positive antibody to hepatitis B core antigen [anti-HBc] antibody test) are eligible. Patients positive for hepatitis C virus (HCV) antibody are eligible only if polymerase chain reaction (PCR) is negative for HCV RNActive tuberculosis
- No history of uncontrolled serious medical or psychiatric illness
- No known hypersensitivity to Chinese hamster ovary cell products or other recombinant human antibodies
- Absence of any psychological, familial, sociological or geographical condition potentially hampering compliance with the study protocol and follow-up schedule
- No current pregnancy or breastfeeding. Women of childbearing potential (WOCBP*) must use adequate contraceptive protection

*) “Women of childbearing potential” is defined as any female who has experienced menarche and who has not undergone surgical sterilization (hysterectomy or bilateral oophorectomy) or who is not postmenopausal. Menopause is defined clinically as 12 months of amenorrhea in a woman over 45 in the absence of other biological or physiological causes.

6 Therapeutic regimens, dose adjustments and management of toxicity

6.1 Atezolizumab

Atezolizumab (MPDL3280A) is a humanized immunoglobulin (Ig) G1 monoclonal antibody consisting of two heavy chains (448 amino acids) and two light chains (214 amino acids) and is produced in Chinese hamster ovary cells. Atezolizumab was engineered to eliminate Fc-effector function via a single amino acid substitution (asparagine to alanine) at position 298 on the heavy chain, which results in a non-glycosylated antibody that has minimal binding to Fc receptors and prevents Fc effector function at expected concentrations in humans. Atezolizumab targets human programmed death-ligand 1 (PD-L1) and inhibits its interaction with its receptors, programmed death-1 (PD-1) and B7.1 (CD80, B7-1). Both of these interactions are reported to provide inhibitory signals to T cells.

Atezolizumab is currently being tested in multiple Phase I, II, and III studies, both as monotherapy and in combination with several anti-cancer therapies (see also Investigator's Brochure). Much of the safety and efficacy data summarized below are from Phase Ia Study PCD4989g, a multicenter, first in-human, open-label, dose-escalation trial evaluating the safety, tolerability, immunogenicity, pharmacokinetics, exploratory pharmacodynamics, and preliminary evidence of biologic activity of atezolizumab administered as a single agent by IV infusion q3w to patients with locally advanced or metastatic solid malignancies or hematologic malignancies. As of 10 May 2014, the clinical database contained preliminary safety data from 412 patients who have received any amount of atezolizumab at doses between 0.01 and 20 mg/kg across multiple tumor types. No dose-limiting toxicities (DLTs) have been observed at any dose level and no maximum tolerated dose (MTD) was established.

Anti-tumor activity including objective responses have been observed in patients with different tumor types, including NSCLC, RCC, melanoma, bladder cancer, CRC, head and neck cancer, gastric cancer, breast cancer, and sarcoma treated with atezolizumab monotherapy in Study PCD4989g. Among 386 evaluable patients enrolled prior to 1 July 2013, there were 47 patients with responses with a median duration of response of 75.7 weeks. The majority of these responses have been durable, with 72.3% (34 of 47 patients) of responses (34 of 47 patients) ongoing as of the clinical cut-off date.

Recently results from the RCC from cohort Study PCD4989g/ NCT01375842 were published[35]. Sixty-three of 70 patients were evaluable for response. The median PFS was 5.6 months (95% CI, 3.9 to 8.2 months), and the median OS was 28.9 months (95% CI, 20.0 months to not reached [NR]). The ORR was 15% (95% CI, 7% to 26%). Analyses of tumor-infiltrating immune cells (ICs) for PD-L1 expression on baseline tumor tissue have been performed for Study PCD4989g/ NCT01375842. Preliminary results from this study suggest that PD-L1 expression on ICs is likely to be associated with response to atezolizumab[36].

As of 2 September 2014, clinical activity analyses have been performed on 21 patients with PD-L1 positive (IC2/3) TNBC in Study PCD4989g who received atezolizumab treatment. Most women had not responded to other treatments and 85% received > 4 prior systemic regimens. Unconfirmed responses were recorded for 5 patients. Two of these patients experienced a complete response and 3 patients experienced a partial response. [31] Arm F from Phase Ib trial GP28328 (TNBC) investigated the combination of atezolizumab with nab-paclitaxel [37]. 24 patients were evaluable for response. The overall response rate (ORR) was 42%, with higher ORR in first line treated patients (67%) compared to third line or higher (29%).

6.1.1 Atezolizumab – Adverse Events

Of the 412 treated patients, 97.1% experienced an adverse event (AE) regardless of attribution to atezolizumab. The majority of these AEs were Grade 1 or 2 (NCI CTCAE v4.03). The most frequently observed AEs (occurring in ±10% of treated patients) included fatigue, nausea, decreased appetite, pyrexia, dyspnea, diarrhea, constipation, cough, headache, back pain, vomiting, anemia, arthralgia, rash, insomnia, asthenia, abdominal pain, chills, pruritus, generalized pain, and peripheral edema.

There were 51 patients (12.4%) who experienced Grade 3 AEs that were assessed as related to study drug by the investigators. The most frequently reported related Grade 3 AEs included fatigue (5 patients [1.2%]), increased AST or ALT (each reported in 4 patients [1.0%]), and asthenia, autoimmune hepatitis, and hypoxia (each reported in 3 patients [0.7%]).

6.1.2 Atezolizumab – Immune-mediated Adverse Events

Given the mechanism of action of atezolizumab, events associated with inflammation and/or immune-mediated AEs have been closely monitored during the atezolizumab clinical program. These include potential dermatologic, hepatic, endocrine, and respiratory events as well as events of hepatitis/elevated liver function tests (LFTs) and influenza like illness that are considered potential adverse drug reactions associated with atezolizumab.

The Adverse Events of Special Interest for atezolizumab are:

- Pneumonitis
- Hypoxemia
- Colitis
- Endocrinopathies: diabetes mellitus, pancreatitis, adrenal insufficiency, hypo- or hyperthyroidism
- Vasculitis
- Hepatitis
- Transaminitis: Grade ≥2, (AST or ALT >3x ULN and bilirubin >2x ULN) or AST/ALT >10x ULN
- Systemic Lupus Erythromatosus
- Guillain Barre Syndrome
- Skin reactions: vitiligo, pemphigus
- Events suggestive of hypersensitivity, cytokine release, influenza like illness, SIRS, or infusion reaction syndromes

6.1.3 Atezolizumab – Safety in combination with chemotherapy

Study GP28328 is a 5 arm Phase Ib trial of the safety and pharmacology of atezolizumab administered with bevacizumab and/or chemotherapy in patients with advanced solid tumors. Arms C, D, and E are evaluating MDPL3280A administered q3w in chemotherapy naïve non-small cell lung cancer (NSCLC) patients in combination with carboplatin and paclitaxel, carboplatin and pemetrexed, and carboplatin and nab-paclitaxel, respectively. As of 10 May 2014, preliminary safety data are available from 90 treated patients. Of these patients, 88 (98%) reported one or more AEs; the majority of the events were Grade 1 and 2 in severity. The five most commonly reported AEs were fatigue, nausea, diarrhea, decreased appetite, and pyrexia. No additive adverse effects have been observed to date when atezolizumab was administered in combination with either bevacizumab or chemotherapy.

6.1.4 Atezolizumab – Administration

The dose of atezolizumab is 1200 mg flat dose on day 1 of a 3-week cycle. Atezolizumab is administered i.v. before the carboplatin infusion. Atezolizumab will be delivered in 250 ml NaCl 0.9%. No premedication is administered. The initial cycle will be given in 60 minutes, and the subsequent infusions in 30 minutes. When atezolizumab is administered as monotherapy, a visit window of +/- 5 days is allowed.

6.1.5 Atezolizumab - Duration of Therapy

Treatment cycles will be repeated until progression (see definition Section 8) unless unacceptable toxicity is encountered. Patients may be treated beyond progression according to RECIST 1.1 (not according to the immune-related response criteria) under protocol-defined circumstances (see Section 8.1). At progressive disease, patients are considered off study and further systemic treatment is at the discretion of the treating physician. After 12 months of atezolizumab treatment discontinuation is allowed in case of ongoing response or stable disease. At signs of progression after discontinuation of the treatment, atezolizumab can be re-started.

6.2. Carboplatin

Carboplatin is a platinum derivative and a highly polar molecule that does not readily diffuse across lipid membranes [38, 39]. In the high chloride environment of plasma, it remains largely in its native form, but once inside the cell, in which the chloride concentration is much lower, it becomes aquated and forms adducts with DNA and reacts with nucleophilic sites in a variety of targets [38]. Compared with cisplatin, carboplatin is essentially devoid of nephrotoxicity, and is less toxic to the gastrointestinal tract and less neurotoxic; by contrast, myelosuppression, principally thrombocytopenia, is dose limiting for carboplatin.
Single-agent carboplatin is active in patients with previously untreated metastatic breast cancer, producing response rates of 20%-35%. [40]

6.2.1 Carboplatin – Administration

The dose of carboplatin is AUC 1.5 in a weekly schedule with a maximum of 12 cycles. This dosage has been used in the GeparSixto study in combination with liposomal doxorubicin and paclitaxel and resulted in less hematological toxicities compared to the AUC 2.0 dosage. [23] Although in most study protocols so far as well as in daily clinical practice carboplatin is used in a 3-week schedule with an AUC 4-6, we strongly feel that for the current study a weekly schedule with a dosage of AUC 1.5 will be more suitable for the following reasons: 1) while high-dose chemotherapy often depletes immune cell subsets, low-dose metronomic chemotherapy exerts a more subtle anti-angiogenic and immunomodulatory mode of action [41-43] 2) less hematological toxicities which makes it less likely that carboplatin (and atezolizumab in case of high grade or persisting toxicities) has to be withhold.
Carboplatin will be reconstituted and administered according to the standard practice in the institution. The carboplatin dose should be calculated according to Calvert's formula as follows: carboplatin dose= target AUC (GFR+25). When administered with atezolizumab, carboplatin will be administered first according to standard practice.
For the purpose of this protocol the GFR is considered equivalent to the creatinine clearance. The exact dose of carboplatin therefore depends on the GFR. The GFR can be calculated using a variety of different formulae and should be calculated as per local practice. Formulae such as the Cockcroft-Gault formula are inaccurate at the extremes of age and weight. The calculated GFR may be falsely high in obese young women and falsely low in thin elderly women. It is assumed that clinicians entering patients into this protocol will be aware of these issues and the clinical judgment of an experienced clinician should be applied to the calculation of the carboplatin dose.

We recommend capping the dose of carboplatin as follows: Using Calvert formula with capping of GFR at 125 mL/min: For a target AUC = 1.5, the maximum dose is 1.5x 150 =225 mg
A measured GFR, using a 24-hour urine collection, is recommended if the serum creatinine is less than or equal to 53 µmol/L, or the calculated GFR is <60ml/min. The lower of the two values of the creatinine clearance should be used to calculate dose.
If any change in GFR of more than 10% is observed, the treatment dosage should be modified accordingly.

6.3 Dose modification for toxicity

- Dose reduction of atezolizumab is not permitted
- When several toxicities with different grades of severity occur at the same time, the dose modifications should be according to the highest grade observed.
- If, in the opinion of the investigator, a toxicity is considered to be due solely to one component of the study treatment (i.e., atezolizumab or carboplatin) and the dose of that compartment is delayed or modified in accordance with the guidelines below, the other component may be administered if there is no contraindication. This dose might be administered after the planned last dose of the chemotherapy to complete the total number of 12 administrations of carboplatin, as determined by the investigator.
- When treatment is temporarily interrupted because of toxicity caused by atezolizumab or chemotherapy the treatment cycles will be restarted such that the atezolizumab and chemotherapy infusions remain synchronized.
- If it is anticipated that chemotherapy will be delayed by ≥ 2 weeks, then atezolizumab should be given without the chemotherapy, if there is no contraindication.

6.3.1 Atezolizumab dose modification and management of specific adverse events

There will be no dose reduction for atezolizumab in this study. Patients may temporarily suspend study treatment if they experience an adverse event that requires a dose to be held. If atezolizumab is held because of adverse events for > 42 days beyond the last dose, then the patient will be discontinued from atezolizumab treatment and will be followed for safety. If, in the judgment of the investigator, the patient is likely to derive clinical benefit from resuming atezolizumab after a hold > 42 days, study drug may be restarted with the approval of the PI.
If a patient must be tapered off steroids used to treat adverse events, atezolizumab may be held for > 42 days until steroids are discontinued or reduced to prednisone dose (or dose equivalent) ≤ 10mg/day. The acceptable length of interruption will depend on agreement between the investigator and the PI.

Dose interruptions for reason(s) other than adverse events, such as surgical procedures, may be allowed by PI approval. The acceptable length of interruption will depend on agreement between the investigator and the PI.
Toxicities associated or possibly associated with atezolizumab treatment should be managed according to standard medical practice. Additional tests, such as autoimmune serology or biopsies, may be used to determine the etiology. Discontinuation of atezolizumab may not have an immediate therapeutic effect and, in severe cases, immune-mediated toxicities may require acute management with topical corticosteroids, systemic corticosteroids, mycophenolate, TNFα inhibitors, or tacrolimus. Further administration of atezolizumab will depend on whether the potential benefits of therapy outweigh the potential risks. Atezolizumab should be permanently discontinued in patients with life-threatening, immune-mediated adverse events. The following sections provide guidance for handling specific adverse events.

6.3.1.1 Gastrointestinal toxicity

Immune-mediated colitis has been associated with the administration of atezolizumab. See table 1 for guidelines on how to manage gastrointestinal toxicity in patients treated with atezolizumab.

Table 1: Dose modification guidelines for atezolizumab-associated gastrointestinal toxicity

Diarrhea/Colitis	Management
**Grade 1**	• Continue therapy<br>• Symptomatic treatment, no steroids<br>• Endoscopy should be considered if symptoms progress and/or persist for > 7 days<br>• Investigate etiology: stool culture and testing for Clostridium diff, Salmonella/Shigella/Campylobacter/Yersinia (consider culture for worms/cysts/eggs)
**Grade 2**<br>(4–6 stools/day over baseline) < 5 days	• Hold atezolizumab and discontinue NSAIDs (or other medications that exacerbate colitis). Ensure adequate rehydration.<br>• Investigate etiology: stool culture and testing for Clostridium diff, Salmonella/Shigella/Campylobacter/Yersinia (consider culture for worms/cysts/eggs)<br>• Administer antidiarrheal agent (e.g., loperamide)<br>• Consult gastroenterologist to perform endoscopy. Endoscopy+biopsies indicated if symptoms persists for >1 week. Endoscopy preferably within 48 hours after start steroids. Investigate possibility of CMV colitis (via biopsies)<br>• In case of moderate/severe colitis at endoscopy; see management advice below
**Grade 2**<br>(4–6 stools/day over baseline) > 5 days or recurs	• Hold atezolizumab and discontinue NSAIDs (or other medications that exacerbate colitis) while etiology is being investigated<br>• Consult gastroenterologist to perform endoscopy. Endoscopy+biopsies indicated if symptoms persists for >1 week. Endoscopy preferably within 48 hours after start steroids. Investigate possibility of CMV colitis (via biopsies)<br>• Investigate etiology: stool culture and testing for Clostridium diff, Salmonella/Shigella/Campylobacter/Yersinia (consider culture for worms/cysts/eggs)<br>• Type of immune suppression depends on severity of inflammation seen during colonoscopy:<br>For <u>mild</u> colitis (no ulcerations): start budesonide 9mg tablet once daily<br>For <u>moderate-severe</u> colitis without ulcerations: start prednisone 1mg/kg/day orally<br>For <u>moderate-severe colitis with significant ulcerations</u> (deep ulceration or ulceration >1cm): start infliximab 5mg/kg iv in combination with prednisone 1mg/kg/day orally.
**Abdominal pain**<br>**Blood or mucus in stool**<br><br>OR<br><br>**Grade 3**<br>(≥ 7 stools/day over baseline) with peritoneal signs, ileus, or fever<br>**OR**<br>**Grade 4**	• Hold atezolizumab and discontinue NSAIDs (or other medications that exacerbate colitis)<br>• Rule out bowel perforation/sepsis, consider admission in hospital for rehydration<br>• Consult gastroenterologist to perform endoscopy. Endoscopy preferably within 48 hours after start steroids. Investigate possibility of CMV colitis (via biopsies)<br>• Investigate etiology: stool culture and testing for Clostridium diff, Salmonella/Shigella/Campylobacter/Yersinia (consider culture for worms/cysts/eggs)<br>• Type of immune suppression depends on severity of inflammation seen during colonoscopy:<br>For <u>moderate-severe</u> colitis without ulcerations: start prednisone 1mg/kg/day orally<br>For <u>moderate-severe colitis with significant ulcerations</u> (deep ulceration or ulceration >1cm): start infliximab 5mg/kg iv in combination with prednisone 1mg/kg/day orally.
**Follow-up after start therapy**	<u>After start budesonide</u>:<br>In case of improvement of symptoms: treat for 4 weeks, after this treat according to symptoms, stop budesonide if free of symptoms and with mild symptoms reduce dosage of budesonide (9mg every other day). In case symptoms persist for 1-2 weeks: repeat investigations. In case no signs of infection: perform endoscopy and start prednison 1mg/kg orally.<br><u>After start prednisone:</u><br>In case of improvement of symptoms: taper prednisone over a period of 4-6 weeks<br>**In case no improvement after 3 days: infliximab 5mg/kg iv and continue prednisone**<br><u>After start infliximab:</u><br>In case of improvement of symptoms: taper prednisone. After infliximab prednisone can be tapered more rapidly (<4 weeks). In case no improvement after 7 days: second administration of infliximab 10mg/kg iv. Consider a diet with only artificial food (via tube or nutritional drinks). Only water and tea without milk and sugar are allowed in this diet. In case no improvement after two cycles of infliximab: consider tacrolimus 0.1-0.2mg/kg/daily orally or 0.01-0.02 mg/kg/daily iv, under control of therapeutic levels every week.
**Other advice**	• Start antibiotics in case of fever (preference for antibiotics indicated for abdominal infections: metronidazole/ceftriaxone).<br>• Only restart atezolizumab after consultation of the PI and treating physician

IV = intravenous; NSAID = nonsteroidal anti-inflammatory drug

6.3.1.2 Hepatotoxicity

Immune-mediated hepatitis has been associated with the administration of atezolizumab.

While on this study, patients presenting with right upper-quadrant abdominal pain and/or unexplained nausea or vomiting should have liver function tests (LFTs) performed immediately and reviewed before administration of the next dose of study drug.

If LFTs increase, neoplastic, concurrent medications, viral hepatitis, and toxic etiologies should be considered and addressed as appropriate. Imaging of the liver, gall bladder, and biliary tree should be performed to rule out neoplastic or other causes for the increased LFTs. Anti-nuclear antibody, perinuclear anti-neutrophil cytoplasmic antibody, anti-liver kidney microsomal antibody, and anti-smooth muscle antibody tests should be performed if an autoimmune etiology is considered.

Patients with liver function test (LFT) abnormalities should be managed according to the guidelines in table 2.

Table 2: Guidelines for Managing Atezolizumab-Associated Hepatotoxicity

LFT Abnormalities	Management
**Grade 1**<br>ASAT/ALAT (> ULN to 3 × ULN) with total bilirubin > ULN to 1.5 × ULN	• Continue atezolizumab.<br>• Continue with the standard monitoring plan
**Grade 2**<br>ASAT/ALAT (> 3 to 5 × ULN with total bilirubin > 1.5 to 3 × ULN	• Continue atezolizumab.<br>• Monitor LFTs at least weekly until return to baseline values.<br>• If persists > 5-7 days: hold atezolizumab and start 60 mg prednisone or equivalent per day, when LFTs ≤ G1, taper steroids over ≥ 1 month, resume therapy when systemic steroid dose is ≤ 10 mg oral prednisone equivalent per day.<br>• Consider GI consult
**Grade 3**<br>ASAT/ALAT > 5 to 20 ULN<br>Total Bilirubin > 3 to 10 x ULN	• Hold atezolizumab.<br>• Consult hepatologist and consider liver biopsy to establish etiology of hepatic injury if necessary.<br>• Start IV steroids (60 mg prednisone or equivalent per day) for 24–48 hours followed by oral prednisone (or equivalent) taper over 2–4 weeks.<br>• If LFT results do not decrease within 48 hours after initiation of systemic steroids, addition of an alternative immunosuppressive agent (e.g., mycophenolate 1000 mg BID orally, taper after 2 weeks) to the corticosteroid regimen may be considered.<br>• Monitor LFTs every 48–72 hours until decreasing and then follow weekly.<br>• Restart atezolizumab after discussion with the PI if ASAT/ALAT ≤ 3 × ULN with bilirubin < 2 × ULN and steroid dose is ≤ 10 mg oral prednisone equivalent per day.<br>• Permanently discontinue atezolizumab for life-threatening immune-related hepatic events.
**Grade 4**<br>ASAT/ALAT ≥ 20 × ULN with bilirubin> 10× ULN	• Hold atezolizumab.<br>• Consult hepatologist and consider liver biopsy to establish etiology of hepatic injury if necessary.<br>• Start IV steroids (60 mg prednisone or equivalent per day) for 24–48 hours followed by oral prednisone (or equivalent) taper over 2–4 weeks.<br>• If LFT results do not decrease within 48 hours after initiation of systemic steroids, addition of an alternative immunosuppressive agent (e.g., mycophenolate 1000 mg BID orally, taper after 2 weeks) to the corticosteroid regimen may be considered.<br>• Monitor LFTs every 48–72 hours until decreasing and then follow weekly.<br>• Permanently discontinue atezolizumab for life-threatening immune-related hepatic events.

IV = intravenous; LFT = liver function test; TNF–α = tumor necrosis factor alpha; ULN = upper limit of normal.

6.3.1.3 Dermatologic toxicity

Treatment-emergent rash has been associated with atezolizumab. The majority of cases of rash were mild in severity and self-limited, with or without pruritus. A dermatologist should evaluate persistent and/or severe rash or pruritus. A tissue sample should be collected for biopsy unless contraindicated.
Dermatologic toxicity and rash should be managed according to the guidelines in Table 3.

Table 3. Guidelines for Managing Atezolizumab-Associated Dermatologic Toxicity

Dermatologic Toxicity/Rash (e.g., Maculopapular or Purpura)	Management
Grade 1, mild, < 10% BSA	• Continue atezolizumab.<br>• Administer symptomatic therapy with antihistamine PRN.<br>• Consider topical steroids and/or other symptomatic therapy (e.g., antihistamines).
Grade 2, moderate, 10%–30% BSA	• Continue atezolizumab.<br>• Consider dermatologist referral.<br>• Administer symptomatic therapy with antihistamine PRN.<br>• Administer topical steroids.<br>• Consider higher potency topical steroids if rash unresolved.
Grade 3, severe, > 30% BSA	• Hold atezolizumab.<br>• Consult dermatologist.<br>• Administer higher potency topical steroids<br>• Administer symptomatic therapy with antihistamine PRN.<br>• If no effect: Administer oral prednisone 10 mg or equivalent. If rash unresolved after 48–72 hours, administer oral prednisone 60 mg or equivalent.<br>• Restart atezolizumab if rash resolved and systemic dose is ≤ 10 mg oral prednisone or equivalent per day.
Grade 4	• Permanently discontinue atezolizumab for life-threatening immune-mediated dermatologic toxicity<br>• Contact PI's if atezolizumab is discontinued.

BSA = body surface area; PRN = as needed.

6.3.1.4 Endocrine toxicity

Thyroid disorders, hypophysitis and adrenal insufficiency have been associated with the administration of atezolizumab. Patients with unexplained symptoms such as fatigue, myalgias, impotence, mental status changes, or constipation should be investigated for the presence of thyroid, pituitary, or adrenal endocrinopathies, as well as for hyponatremia or hyperkalemia. An endocrinologist should be consulted if an endocrinopathy is suspected. TSH and free T4 levels should be obtained to determine whether thyroid abnormalities are present. TSH, prolactin, and a morning cortisol level will help to differentiate primary adrenal insufficiency from primary pituitary insufficiency. Endocrine toxicity should be managed according to the guidelines in Table 4.

Table 4: Guidelines for Managing Atezolizumab-Associated Endocrine Toxicity

**Severity**	**Management**
**Asymptomatic Hypothyroidism**	• Continue atezolizumab.<br>• Start thyroid replacement hormone.<br>• Monitor TSH weekly.
**Symptomatic Hypothyroidism**	• Hold atezolizumab.<br>• Start thyroid replacement hormone therapy<br>• Consider referral to an endocrinologist.<br>• Restart atezolizumab when symptoms are controlled by thyroid replacement and TSH levels are decreasing.
**Asymptomatic Hyperthyroidism**	• If serum TSH < 0.5 mU/l, repeat with free T4 and T3 to diagnose asymptomatic hyperthyroidism<br>• If confirmed, and in the absence of symptoms, continue atezolizumab and repeat tests in 4 weeks.<br>• If thyroid values remain stable and patient is asymptomatic, follow labs monthly<br>• Hold atezolizumab if TSH < 0.1 mU/l or if patient develops symptomatic hyperthyroidism<br>• Consider referral to an endocrinologist and treat according to symptomatic hyperthyroidism guidelines.
**Symptomatic Hyperthyroidism**	• Hold atezolizumab<br>• Start beta-blockers, eventually methimazole as needed<br>• Consider referral to an endocrinologist<br>• Restart atezolizumab when symptoms are controlled by therapy<br>• Permanently discontinue atezolizumab for life threatening immune related hyperthyroidism
**Symptomatic panhypopituitarism and any grade 3-4 events**	• Hold atezolizumab<br>• Consult endocrinologist<br>• Perform appropriate imaging<br>• Treat with an initial dose of methylprednisolone 1 to 2 mg/kg per day intravenously followed by oral prednisone 1 to 2 mg/kg per day<br>• Taper steroids when symptoms improve to G0 or G1. Taper over > 1 month.<br>• Restart atezolizumab when symptoms are controlled by therapy

TSH = thyroid-stimulating hormone; T4 = thyroxine

6.3.1.5 Pulmonary toxicity

Dysnea, cough, fatigue, hypoxia, and pulmonary infiltrates have been associated with the administration of atezolizumab and have primarily been observed in patients with underlying NSCLC.

Appropriate workup for pulmonary adverse events should include the following as appropriate, as well as ruling out alternative causes (e.g., lymphangitic carcinomatosis, infection, heart failure, or chronic obstructive pulmonary disease or pulmonary hypertension):

- Measurement of oxygen saturation (i.e., arterial blood gas)
- High-resolution CT scan of the chest
- Bronchoscopy with bronchoalveolar lavage and biopsy
- Pulmonary function tests (with diffusion capacity of the lung for carbon monoxide)

Pulmonary function testing and CT with a pulmonary embolism protocol may also be helpful in the diagnostic evaluation. For patients with clinical symptoms, treatment should include administration of corticosteroids and/or oxygen when indicated. Consultation with a pulmonologist is appropriate for a suspected lung immune-related adverse event, and a bronchoscopy with biopsy should be performed, unless contraindicated, prior to the administration of corticosteroids.

Patients will be assessed for pulmonary signs and symptoms throughout the study. Pulmonary toxicity should be managed according to the guidelines in Table 5.

Table 5: Guidelines for Managing Atezolizumab-Associated Pulmonary Toxicity

**Pulmonary Toxicity**	**Management**
**Grade 1**	• May continue atezolizumab with close monitoring<br>• Re-evaluate on serial imaging<br>• Consider consultation of pulmonologist
**Grade 2**	• Hold atezolizumab.<br>• Consult a pulmonologist; investigate for other etiologies and consider bronchoscopy/BAL.<br>• If bronchoscopy is consistent with immune-related etiology, start 60 mg prednisone equivalent per day<br>• When improves to G0 or G1, then taper steroids over ≥1 month<br>• Atezolizumab may be resumed if the event improves to G0 or 1 within 12 weeks and corticosteroids have been reduced to the equivalent of oral prednisone 10 mg daily or less.<br>• Treat as G3/4 if recurrent episode of pneumonitits.
**Grade 3 and 4**	• Permanently discontinue atezolizumab<br>• Consult a pulmonologist; investigate for other etiologies and bronchoscopy/BAL is recommended.<br>• Start 60 mg prednisone equivalent per day<br>• When improves to G0 or G1, then taper steroids over ≥1 month<br>• If not improving after 48 hrs or worsening: add additional immunosuppression (e.g.infliximab, iv immunoglobuline, or mycophenolate mofetil)<br>• Contact PI if atezolizumab is discontinued.

6.3.1.6 Pancreatic toxicity

Symptoms of abdominal pain associated with elevations of amylase and lipase suggestive of pancreatitis have been associated with the administration of other immunomodulatory agents. The differential diagnosis of acute abdominal pain should include pancreatitis. Appropriate workup should include an evaluation for obstruction, as well as serum amylase and lipase tests. Pancreatic toxicity should be managed according to the guidelines in Table 6.

Table 6. Guidelines for Managing Atezolizumab-Associated Pancreatic Toxicity

**Amylase/Lipase Abnormalities**	**Management**
**Grade 1**<br>Amylase/lipase (>ULN and ≤ 2 × ULN) and asymptomatic	• Continue atezolizumab.<br>• Monitor amylase/lipase levels prior to dosing.
**Grade 2**<br>Amylase/lipase (> 2 × ULN to ≤ 5 × ULN) and asymptomatic	• Continue atezolizumab.<br>• Monitor amylase/lipase weekly.
**Grade 3 and 4**<br>Amylase/lipase (> 5 × ULN) and asymptomatic	• Hold atezolizumab.<br>• Consult a gastroenterologist<br>• Monitor amylase/lipase every other day and only consider oral prednisone 60 mg daily or equivalent if symptoms start to occur,<br>• When lab abnormalities return to G0 or G1, then taper steroids over ≥ 1 month<br>• Atezolizumab may be resumed if the event resolves to G0 or G1 within 12 weeks and corticosteroids have been reduced to the equivalent of oral prednisone 10 mg daily or less<br>• Permanently discontinue atezolizumab for recurrent symptomatic grade 3 or 4 amylase/lipase
**Autoimmune pancreatitis** (abdominal pain and raised amylase/lipase levels)	• Hold atezolizumab.<br>• Consult a gastroenterologist.<br>• Administer IV steroids (prednisone equivalent of 60 mg/day) and convert to oral steroids when symptoms improve.<br>• When symptoms and lab abnormalities improves to G0 or G1, then taper steroids over ≥ 1 month<br>• Atezolizumab may be resumed if the event improves to G0 or G1 within 12 weeks and corticosteroids have been reduced to the equivalent of prednisone 10 mg po daily or less<br>• Permanently discontinue atezolizumab for life-threatening immune-related pancreatitis or recurrent pancreatitis.

IV = intravenous; ULN = upper limit of normal.

6.3.1.7 Potential eye toxicity

Patients in the study are encouraged to maintain eye hydration, generally through the use of moisturizing eye drops. An ophthalmologist should evaluate visual complaints. Uveitis or episcleritis may be treated with topical corticosteroid eye drops. Atezolizumab should be permanently discontinued for immune-mediated ocular disease that is unresponsive to local immunosuppressive therapy. Ocular toxicity should be managed according to the guidelines in Table 7.

Table 7. Guidelines for Managing Atezolizumab-Associated Eye Toxicity

**Symptomatic eye toxicity (autoimmune uveitis, iritis, or episcleritis**	**Management**
Grade 1-2	• Evaluation by an ophthalmologist is strongly recommended<br>• Treat with topical corticosteroid eye drops<br>• Discontinue atezolizumab if symptoms persist despite treatment with topical immunosuppressive therapy<br>• Topical immunosuppressive therapy<br>• Contact PI if atezolizumab is discontinued.
Grade 3-4	• Hold atezolizumab.<br>• Start 60 mg prednisone or equivalent per day<br>• Taper steroids over ≥ 1 month once symptoms improve to G0 or G1<br>• Contact PI if atezolizumab is discontinued.

6.3.1.8 Systemic Immune Activation

Systemic immune activation (SIA) is a rare condition characterized by an excessive immune response. Given the mechanism of action of atezolizumab, SIA is considered a potential risk. SIA should be included in the differential diagnosis for patients who develop a sepsis-like syndrome after administration of atezolizumab, and initial workup should include serum ferritin, complete blood count, LFTs, serum triglycerides, and a coagulation profile. In the event of suspected SIA, the PI's should be contacted for additional recommendations. Treatment with agents such as tocilizumab (anti-IL6), as well as corticosteroids, should be considered in the event of SIA.

6.3.1.9 Neurologic Disorders

Myasthenia gravis and Guillain-Barre syndrome have been observed with single agent atezolizumab. Patients may present with signs and symptoms of sensory and/or motor neuropathy. Diagnostic work-up is essential for an accurate characterization to differentiate between alternate etiologies. Neurotoxicity should be managed according to the guidelines in Table 8.

Table 8 Management Guidelines for Neurologic Disorders

Autoimmune Neuropathy	Management
**Grade 1**	• Continue atezolizumab, evaluate for alternative causes
**Grade 2**	• Hold atezolizumab.<br>• Evaluate for alternative causes<br>• Treatment should be as per institutional guidelines
**Grade 3-4**	• Permanently discontinue atezolizumab for life-threatening immune-related neuropathy<br>• Treatment should be as per institutional guidelines
**Myasthenia Gravis and Guillain-Barre**	• Permanently discontinue atezolizumab for myasthenia gravis or Guillain-Barre (all grades)<br>• Treatment should be as per institutional guidelines

6.3.1.10 Infusion-Related Reactions

No premedication is indicated for administration of cycle 1 of atezolizumab. Patients who experience an infusion-related-reaction (IRR) with cycle 1 of atezolizumab may receive premedication with antihistamines or antipyretics/analgesics (e.g. acetaminophen) for subsequent infusions. See table 9 for further advice.

Table 9 Management Guidelines for Infusion-Related Reactions

Infusion Reactions	Management
**Grade 1**	• Reduce infusion rate to half the rate being given at the time of onset.<br>• Once the event has resolved, the nurse should wait for 30 minutes while delivering the infusion at the reduced rate.<br>• If tolerated, the infusion rate may then be increased to original rate.
**Grade 2**	• Interrupt atezolizumab infusion<br>• Administer aggressive symptomatic treatment<br>• Restart only after the symptoms have adequately resolved to baseline grade<br>• The infusion rate at restart should be half of the infusion rate that was in progress at the time of the onset of the IRR<br>• At next cycle, administer oral premedication with antihistamine and anti-pyretic and monitor closely for infusion reaction
**Grade 3-4**	• Stop infusion<br>• Proper medical management which may include oral or IV antihistamine, anti-pyretic, glucocorticoids, epinephrine, bronchodilators, and oxygen<br>• Discontinue atezolizumab and contact PI if atezolizumab is discontinued.

6.3.1.11 Immune-related myositis

Myositis or inflammatory myopathies are a group of disorders sharing the common feature of inflammatory muscle injury. Initial diagnosis is based on clinical (muscle weakness, muscle pain, skin rash in dermatomyositis), biochemical (serum creatine-kinase increase), and imaging (electromyography/MRI) features, and is confirmed with a muscle-biopsy. See table 10 for management guidelines of immune-related myositis.

Table 10: management guidelines for Immune-related myositis

Immune-related myositis	Management
**Grade 1**	• Continue atezolizumab<br>• Refer patient to rheumatologists or neurologist<br>• Initiate treatment as per institutional guidelines
**Grade 2**	• Withhold atezolizumab for up to 12 weeks after event onset<br>• Refer patient to rheumatologists or neurologist<br>• Initiate treatment as per institutional guidelines<br>• Consider treatment with corticosteroid equivalent to 1-2 mg/kg/day IV methylprednisolone and convert to 1-2 mg/kg/day oral prednisone or equivalent upon improvement<br>• If corticosteroids are initiated and event does not improve within 48 hours after initiating corticosteroids, consider adding an immunosuppressive agent.<br>• If event resolves to Grade 1 or better, resume atezolizumab<br>• If event does not resolve to Grade 1 or better while withholding atezolizumab, permanently discontinue atezolizumab
**Grade 3**	• Withhold atezolizumab for up to 12 weeks after event onset and contact Medical Monitor.<br>• Refer patient to rheumatologist or neurologist.<br>• Initiate treatment as per institutional guidelines.<br>• Respiratory support may be required in more severe cases.<br>• Initiate treatment with corticosteroids equivalent to 1 mg/kg/day IV methylprednisolone or higher-dose bolus if patient is severely compromised (e.g. cardiac or respiratory symptoms, dysphagia, or weakness that severely limits mobility); convert to 1-2 mg/kg/day oral prednisolone or equivalent upon improvement.<br>• If event does not improve within 48 hours after initiating corticosteroids, consider adding an immunosuppressive agent.<br>• If event resolves to Grade 1 or better, resume atezolizumab.<br>• If event does not resolve to Grade 1 or better while withholding atezolizumab, permanently discontinue atezolizumab and contact Medical Monitor.<br>• For recurrent events, treat as a Grade 4 event.
**Grade 4**	• Permanently discontinue atezolizumab and contact Medical Monitor.<br>• Refer patient to rheumatologist or neurologist.<br>• Initiate treatment as per institutional guidelines. Respiratory support may be required in more severe cases.<br>• Initiate treatment with corticosteroids equivalent to 1-2 mg/kg/day IV methylprednisolone or higher-dose bolus if patient is severely comprom ised (e.g. cardiac or respiratory symptoms, dysphagia, or weakness that severely limiting mobility); convert to 1-2 mg/kg/day oral prednisone or equivalent upon improvement.<br>• If event does not improve within 48 hours after initiating corticosteroids, consider adding an immunosuppressive agent.<br>• If event resolves to Grade 1 or better, taper corticosteroids over > 1 month.

6.3.2 Carboplatin dose modification

Hematological toxicity

Treatment with carboplatin should be withheld if either of the following occurs within 24 hours prior to the administration:

- ANC is less than 1 x 10^9 /l (G-CSF is not allowed)
- PLT count is less than 100x10^9/l.

Carboplatin will be restarted if hematological recovery occurs. Cycles will not be delayed but will be canceled, e.g. carboplatin will be administered during the first 12 weeks of the trial and not after this period and a cycle will not be given at a later date. Complete blood cell count should be repeated weekly until hematological recovery has occurred (ANC ≥ 1 x10^9/l and PLT ≥ 100 x10^9/l). No dose modification is allowed.

Renal toxicity

The combination of carboplatin with atezolizumab is not directly expected to cause renal toxicity. Therefore, no specific dose modifications are recommended for renal toxicity. However, the administered dose of carboplatin must be recalculated, based on a recalculated or re-measured eGFR for

- Renal toxicity (CTC grade 2, serum creatinine > 1.5 x ULN)
- Changes in serum creatinine of ≥10%
- Each dose modification of carboplatin
- Cycle 2, if there has been significant doubt about the true eGFR at cycle 1 (e.g. due to significant ascites)

Hypersensitivity to carboplatin

If there is a hypersensitivity reaction to carboplatin this should be managed as per local institutional protocols.

Peripheral neuropathy

If grade 2 toxicity develops, carboplatin should be held until neuropathy recovers (atezolizumab should continue as scheduled). If grade 2 neuropathy persists for > 2 weeks or recurs after carboplatin withhold, the patient will discontinue carboplatin treatment, but may continue atezolizumab.

Gastrointestinal toxicity

Nausea and vomiting should be controlled with standard anti-emetics and will not result in dose modification.

Other toxicities

If the patient develops any other grade 3 or 4 toxicity thought to be related to carboplatin, carboplatin should be held until symptoms resolve to ≤ grade 1 or baseline (atezolizumab treatment should continue as scheduled).

6.4 Co-medication

All concomitant medication must be documented in the patients' file. No other anticancer agents or investigational drugs are allowed during the study or within 14 days of the inclusion into the trial, nor the participation into another study.

- Supportive treatment with anti-emetics is allowed.
- Palliative radiation to symptomatic lesions is allowed (outside the brain) after start of atezolizumab, as long as the response to atezolizumab can still be evaluated. It is not required to hold atezolizumab during palliative radiotherapy; chemotherapy should be interrupted per institutional standard of care
- Bisphosphonates are permitted, as long as these have been started at least 1 month ago.
- Use of G-CSF is not routinely allowed.
- Subjects are permitted to use topical, ocular, intra-articular, intranasal, and inhalational corticosteroids (with minimal systemic absorption). Physiologic replacement doses of systemic corticosteroids are permitted, even if > 10 mg/day prednisone equivalents. A brief course of corticosteroids for prophylaxis (eg, contrast dye allergy) or for treatment of non-autoimmune conditions (eg, delayed-type hypersensitivity reaction caused by contact allergen) is permitted.

6.5 Pregnancy and breastfeeding

Pregnancy testing

All women who are being considered for participation in the study, and who are not surgically sterilized or postmenopausal (no menses for more than two years) will be tested for pregnancy within 28 days before enrolment.

Contraception

Male and female patients will be informed that taking the study medication may involve unknown risks to the foetus if pregnancy were to occur during the study. In order to participate in the study they must adhere to the contraception requirements during the study from the time of screening until 30 days after the last dose of study medication. The following are considered adequate contraceptive methods in this study:

- Abstinence
- Placement of a copper intrauterine device (IUD)
- Male or female sterilisation.

Breastfeeding

Because of the potential for serious adverse reactions in the nursing infant, patients who are breast-feeding are not eligible for enrolment.

Pregnancy

If a patient inadvertently becomes pregnant while on treatment, the patient will immediately be removed from the study. The Principal Investigator will immediately be informed. The patient will be followed on a regular basis until the pregnancy has been completed of terminated. The outcome of the pregnancy will be reported as a SAE in case of death, spontaneous miscarriage, congenital anomaly or other disabling of life-threatening complications to the mother or newborn.

If a male patient's partner becomes pregnant on study the pregnancy must be reported to the sponsor. Every effort will be made to obtain permission to follow the outcome of the pregnancy and report the condition of the foetus or newborn to the sponsor.

7 Clinical evaluation, laboratory tests, follow-up

7.1 Before treatment

7.1.1 Baseline screening

- Signing Informed Consent
- Collect fresh biopsies of a metastatic lesion (non-bone) for FFPE and frozen tissue. The biopsy has to contain sufficient tumor content (≥100 tumor cells); subjects with samples that have insufficient tumor content will require re-biopsy prior to induction treatment. Collect 3x16 G biopsies (1 for FFPE and 2 for frozen).
- Confirmation of the lobular histology on a biopsy of a metastatic lesion – or if unavailable on the primary tumor- by local pathologist. Loss of E-cadherin expression or aberrant E-cadherin staining is required.
- Confirmation of estrogen receptor expression of at least 10% on a biopsy of a metastatic lesion
- Medical history, including menopausal status
- Current medication
- Documentation of persistent toxicities from earlier treatments
- Full physical examination, including weight and height, blood pressure, pulse and WHO performance status (Appendix B)
- Laboratory testing: hematology (Hb, leukocytes and differentiation, platelets), biochemistry (glucose, creat, urea, sodium, potassium, magnesium, chloride, calcium, phosphate, albumin, bilirubin, yGT, AF, AST, ALT, LDH, CRP, TSH, free T4, amylase, lipase, ACTH, cortisol, FSH, LH, E2), serology hepatitis B/C (HbsAg, anti-HBc, HCV Ab), CA15.3, CEA, HIV test, PT and APTT
- B-HCG pregnancy test in all women of childbearing potential (urine or blood)
- Electrocardiogram
- Tumor measurements according to RECIST 1.1 by CT scan thorax/abdomen (not older than 4 weeks at time of start carboplatin)

7.1.2 Before first carboplatin (induction day 1)

- Symptom directed physical examination
- Laboratory testing to be repeated if baseline> 3 days ago
- Blood samples for biobanking (translational research purpose).
- Collect stool sample for microbiome analysis (optional)

7.2 During treatment

7.2.1 Before second cycle of carboplatin (induction day 8)

- Symptom directed physical exam
- Adverse events of grade ≥ 2
- Laboratory testing: hematology (Hb, leukocytes and differentiation, platelets), biochemistry (creat, urea, sodium, potassium, calcium, albumin, bilirubin, yGT, AF, AST, ALT, LDH).

7.2.2 Before start atezolizumab cycle 1/carboplatin no 3 (cycle 1, day 1)

- Collect fresh biopsies of a metastatic lesion for FFPE and frozen tissue. Biopsies preferably taken from the lesion biopsied at baseline taking into account the risk of complications during the

procedure and sufficient volume of the lesion in case of objective response. Collect 3x16 G biopsies (1 for FFPE and 2 for frozen).

- Tumor measurements by CT scan thorax/abdomen
- Symptom directed physical exam
- Adverse events of grade ≥ 2
- Laboratory testing: hematology (Hb, leukocytes and differentiation, platelets), biochemistry (glucose, creat, urea, sodium, potassium, magnesium, chloride, calcium, phosphate, albumin, bilirubin, yGT, AF, AST, ALT, LDH, CRP, TSH, free T4, amylase, lipase, ACTH, cortisol, FSH, LH, E2), CA15.3, CEA
- Blood samples for biobanking (translational research purpose).
- Collect stool sample for microbiome analysis (optional)

7.2.3 During weekly carboplatin administration (day 8 and 15 of cycle 1,2 and 3)

- Symptom directed physical exam
- Adverse events of grade ≥ 2 and the Adverse Events of Special Interest for atezolizumab
- Laboratory testing: hematology (Hb, leukocytes and differentiation, platelets), biochemistry (creat, urea, sodium, potassium, calcium, albumin, bilirubin, yGT, AF, AST, ALT, LDH).

7.2.4 Before start atezolizumab cycle 2 /carboplatin 6 (day 1 of cycle 2)

- Symptom directed physical exam
- Adverse events of grade ≥ 2 and the Adverse Events of Special Interest for atezolizumab
- Laboratory testing: hematology (Hb, leukocytes and differentiation, platelets), biochemistry (glucose, creat, urea, sodium, potassium, magnesium, chloride, calcium, phosphate, albumin, bilirubin, yGT, AF, AST, ALT, LDH, CRP, TSH, free T4, amylase, lipase, ACTH, cortisol, FSH, LH, E2), CA15.3, CEA

7.2.5 Before start atezolizumab cycle 3/ carboplatin 9 (day 1 of cycle 3)

- Collect fresh biopsies of a metastatic lesion for FFPE and frozen tissue. Biopsies preferably taken from the lesion biopsied at baseline taking into account the risk of complications during the procedure and sufficient volume of the lesion in case of objective response. Collect 3x16 G biopsies (1 for FFPE and 2 for frozen).
- Tumor measurements by CT scan thorax/abdomen
- Symptom directed physical exam
- Adverse events of grade ≥ 2 and the Adverse Events of Special Interest for atezolizumab
- Laboratory testing: hematology (Hb, leukocytes and differentiation, platelets), biochemistry (glucose, creat, urea, sodium, potassium, magnesium, chloride, calcium, phosphate, albumin, bilirubin, yGT, AF, AST, ALT, LDH, CRP, TSH, free T4, amylase, lipase, ACTH, cortisol, FSH, LH, E2), CA15.3, CEA
- Blood samples for biobanking (translational research purpose).
- Collect stool sample for microbiome analysis (optional)

7.2.6 From atezolizumab cycle 4

During every cycle:

- Symptom directed physical exam
- Adverse events of grade ≥ 2 and the Adverse Events of Special Interest for atezolizumab

- Laboratory testing: hematology (Hb, leukocytes and differentiation, platelets), biochemistry (glucose, creat, urea, sodium, potassium, magnesium, chloride, calcium, phosphate, albumin, bilirubin, yGT, AF, AST, ALT, LDH, CRP, TSH, free T4, amylase, lipase, ACTH, cortisol, FSH, LH, E2), CA15.3, CEA

Every 6 weeks, every 9 weeks after 6 months:

- Tumor measurements by CT scan thorax/abdomen **(official response evaluation)**. Measurements should continue until progression, also in case patient is taken off study for another reason.

Optional (for other centers than the NKI): before start atezolizumab cycle 5 and cycle 7 (day 1 of cycle 5 and 7)

- Blood samples for biobanking (translational research purpose).

7.3 At progression

- For patients who progress after initial response as defined by RECIST: in case a metastatic lesion is accessible and the patient is willing to undergo a biopsy: collect fresh biopsies of a metastatic lesion for FFPE.
- For patients who progress after initial response as defined by RECIST: collect blood samples for biobanking (translational research purpose)
- Tumor measurements using CT. Patients may be treated beyond progression under protocol-defined circumstances (see Section 8.1).
- Laboratory testing: hematology (Hb, leukocytes and differentiation, platelets), biochemistry (glucose, creat, urea, sodium, potassium, magnesium, chloride, calcium, phosphate, albumin, bilirubin, yGT, AF, AST, ALT, LDH, CRP, TSH, free T4, amylase, lipase, ACTH, cortisol, FSH, LH, E2), CA15.3, CEA

Follow up after progression

- After progression the patient will go off study. Patient who were referred to the NKI or other study centers for the current study can have their follow up at the referring hospital as long as the NKI or the other study center will be informed regarding date and cause of death. Information on subsequent treatment will be collected.

7.4 Ongoing response or stable disease at 12 months (18 cycles)

Atezolizumab will be given until tumor progression or intolerable toxicity. After 12 months of atezolizumab treatment discontinuation is allowed in case of ongoing response or stable disease. At signs of progression after discontinuation of the treatment, atezolizumab can be re-started. The follow-up in this treatment-free period should be done as follow:

- Every four weeks: symptom directed physical exam, collect information on adverse events. Laboratory testing: hematology (Hb, leukocytes and differentiation, platelets), biochemistry (glucose, creat, urea, sodium, potassium, magnesium, chloride, calcium, phosphate, albumin, bilirubin, yGT, AF, AST, ALT, LDH, CRP, TSH, free T4, CEA, CA15.3.
- Every eight weeks: CT-scan (after 6 months: every 12 weeks).

7.5 Summary table

Visit description	Screening	Induc-tion		Cycle 1			Cycle 2			Cycle 3			Cycle 4	Cycle 5 onwards	At 12 months (with ongoing response or SD)	At progression	Survival
**Visit window (days)**		1	8	1	8	15	1	8	15	1	8	15	1	1			
**Carboplatin**		1	2	3	4	5	6	7	8	9	10	11	12				
**Atezolizumab**				1			2			3			4	5->			
**Informed consent**	x																
**Confirmation of lobular histology**	x																
**Biopsy**[1]	x[2]			x						x						x[3]	
**Blood for biobank lab**[4]	x	x[5]		x[5]						x[5]				X[4]		x[3]	
**CT chest/abdomen (incl tumor measurements)**	x			x						x				x[6]	X[7]	x	
**Medical history, current medication and baseline toxicity**	x																
**Physical examination**[11]	x	x	x	x	x	x	x	x	x	x	x	x	x	x	X		
**Hematology/Chemistry**[8]	x	x	x[9]	x	x 9	x[9]	x	x[9]	x 9	x	x[9]	x[9]	x	x	X	x	
**PT/INR and aPTT**	x																
**Markers/Endocrinology**[10]	x			x			x			x			x	x		x	
**Pregnancy test**[12]	x																
**Hepatitis/HIV testing**	x																
**ECG**	x																
**Adverse events**[11]			x	x	x	x	x	x	x	x	x	x	x	x			
**Survival and subsequent treatment**																	x
Optional: **stool for microbiome analysis**		x[3]		x[3]						x[3]							

1 3x 16G biopsies: 1 for FFPE, 2 for frozen. Aim at taking sequential biopsies from same lesion
2 Mandatory: sufficient number of tumor cells (≥100) has to be present before patient can enter the study
3 Optional
4 PBMCs, serum, ctDNA, cfDNA, tumor-educated platelets. At baseline only; material for normal germline DNA. Before cycle 5 and 7 (optional for other centers than the NKI).
5 At NKI: material for Breast Immuno Flow (real-time comprehensive analyses off immune cell subpopulations including myeloid cells, de Visser lab)
6 Every 6 weeks, after 6 months every 9 weeks
7 Every 8 weeks, after 6 months every 12 weeks
8 Hb, leukocytes incl differentiation, platelets, creat, glucose, urea, sodium, potassium, magnesium, calcium, chloride, bilirubin, gGT, AF, AST, ALT, LDH, CRP, phosphate, albumine
9 Hb, leukocytes incl differentiation, platelets, creat, urea, sodium, potassium, calcium, bilirubin, gGT, AF, AST, ALT, LDH
10 CA15.3, CEA. TSH, fT4, cortisol, ACTH, amylase, lipase, E2, FSH, LH
11 AEs will be graded according to CTCAE 4.03, SAE need to be reported up to 30 days post last atezolizumab study medication infusion
12 at baseline full examination, including weight and height, blood pressure, pulse and WHO performance status. During follow-up symptom directed
13 only in women of child birth potential

8 Response evaluation criteria

Response to treatment will be assessed locally and centrally. Local assessment will be used for treatment decisions during the course of the study and for assessment of the primary endpoint. After completion of the accrual, all scans made for evaluation of the disease of enrolled patients will be reviewed centrally.

8.1 Time points of response measurements

RECIST 1.1 measurements during the study will be compared with RECIST at screening. The first official response evaluation will take place after 4 cycles of atezolizumab. In case of initial progression, subjects will be permitted to continue atezolizumab in case of clear clinical benefit as defined in section 8.2 according to iRECIST. [17].

8. 2 Modified RECIST 1.1 for immune-based therapeutics (iRECIST)

In rare occasions during first evaluation (after 4 cycles) of response to atezolizumab imaging may show small new lesions in the presence of other responsive lesions. In these cases, response evaluation according to iRECIST requires confirmation of progression by a repeat, consecutive assessment no less than 4 weeks from the date first documented, while RECIST 1.1 will define this as PD. If no further progression is seen on the confirmation scan, treatment will be continued according to protocol and imaging will be performed according to the original schedule.

Accumulating evidence indicates a minority of subjects treated with immunotherapy may derive clinical benefit despite initial evidence of PD.[16] At ASCO 2014 it has been presented that conventional criteria such as RECIST [34] may underestimate the benefit of anti-PD1 in approximately 10% of treated melanoma patients. [44] In this view, subjects will be permitted to continue atezolizumab treatment beyond initial RECIST 1.1 defined PD as long as they meet the following criteria (according to iRECIST):

- Investigator-assessed clinical benefit
- The possible progression seen on imaging is not putting the patient at risk for tumor-related complications
- Subject is tolerating study drug

The assessment of clinical benefit should take into account whether the subject is clinically deteriorating and unlikely to receive further benefit from continued treatment. All decisions to continue treatment beyond initial progression must be discussed with the investigator and documented in the study records. Subjects should discontinue study therapy upon further evidence of further progression, defined as an additional 10% or greater increase in tumor burden volume from time of initial progression
(including all target lesions and new measurable lesions). New lesions are considered measurable at the time of initial progression if the longest diameter is at least 10 mm (except for pathological lymph nodes, which must have a short axis of at least 15 mm). Any new lesion considered non-measurable at the time of initial progression may become measurable and therefore included in the tumor burden measurement if the longest diameter increases to at least 10 mm (except for pathological lymph nodes, which must have an increase in short axis to at least 15 mm). For statistical analyses that include the investigator-assessed progression date, subjects who continue treatment beyond initial investigator-assessed, RECIST 1.1-defined progression will be considered to have investigator-assessed progressive disease at the time of the initial progression event (according to iRECIST).

8.3. Evaluation of efficacy by RECIST

For patients with metastatic disease deemed non-measurable by RECIST criteria, determination of tumor response including disease progression will be made by the investigator using clinical judgement and relevant radiology, according to standard practice.

Response criteria are essentially based on a set of measurable lesions identified at baseline as target lesions, and – together with other lesions that are denoted as non-target lesions – followed until disease progression.

The following paragraphs are a quick reference to the RECIST criteria (version 1.1). The complete criteria are included in the published RECIST document [34] also available at http://www.eortc.be/RECIST.

8.3.1 Measurability of tumor lesions at baseline

Definitions

- **Measurable disease** - the presence of at least one measurable lesion. If the measurable disease is restricted to a solitary lesion, its neoplastic nature should be confirmed by cytology/histology.
- **Measurable lesions** - *tumor lesions* that can be accurately measured in at least one dimension (longest diameter to be recorded) as ≥ 20 mm with chest x-ray, and as ≥ 10 mm with CT scan or clinical examination [using calipers]. Bone lesions are considered measurable only if assessed by CT scan and have an identifiable soft tissue component that meets these requirements (soft tissue component ≥ 10 mm by CT scan). *Malignant lymph nodes* must be ≥ 15 mm in the short axis to be considered measurable; only the short axis will be measured and followed. All tumor measurements must be recorded in millimeters (or decimal fractions of centimeters) by use of a ruler or calipers. Tumor lesions situated in a previously irradiated area, or in an area subjected to other loco-regional therapy, are usually not considered measurable unless there has been demonstrated progression in the lesion.
- Non-measurable lesions - All other lesions (or sites of disease), including small lesions are considered non-measurable disease. Bone lesions without a measurable soft tissue component, leptomeningeal disease, ascites, pleural/pericardial effusions, lymphangitis cutis/pulmonis, inflammatory breast disease, lymphangitic involvement of lung or skin and abdominal masses followed by clinical examination are all non-measurable. Nodes that have a short axis <10 mm at baseline are considered non-pathological and should not be recorded or followed.
- **Target Lesions.** When more than one measurable tumor lesion or malignant lymph node is present at baseline all lesions up to *a maximum of 5 lesions total* (and a maximum of *2 lesions per organ*) representative of all involved organs should be identified as target lesions and will be recorded and measured at baseline. Target lesions should be selected on the basis of their size (lesions with the longest diameter), be representative of all involved organs, but in addition should be those that lend themselves to *reproducible repeated measurements*. Note that pathological nodes must meet the criterion of a short axis of ≥ 15 mm by CT scan and only the *short* axis of these nodes will contribute to the baseline sum. At baseline, the sum of the target lesions (longest diameter of tumor lesions plus short axis of lymph nodes: overall maximum of 5 is to be calculated and recorded.

- **Non-target Lesions**. All non-measurable lesions (or sites of disease) including pathological nodes (those with short axis ≥ 10 mm but < 15 mm), plus any measurable lesions over and above those listed as target lesions are considered *non-target lesions*. Measurements are not required but these lesions should be noted at baseline and should be followed as "present" or "absent".
- All baseline evaluations should be performed as closely as possible to the beginning of treatment and never more than 4 weeks before the beginning of the treatment.

Methods of measurements

The same method of assessment and the same technique should be used to characterize each identified and reported lesion at baseline and during follow-up. Assessments should be identified on a calendar schedule and should not be affected by delays in therapy, which may be treatment arm dependent. While on study, all target lesions recorded at baseline should have their actual measurements recorded on the CRF at each subsequent evaluation, even when very small (e.g. 2 mm). If it is the opinion of the radiologist that the lesion has likely disappeared, the measurement should be recorded as 0 mm. If the lesion is believed to be present and is faintly seen but too small to measure, a default value of 5 mm should be assigned. For lesions which fragment/split add together the longest diameters of the fragmented portions; for lesions which coalesce, measure the maximal longest diameter for the "merged lesion".

- **Clinical Lesions**. Clinical lesions will only be considered measurable when they are superficial and ≥ 10 mm as assessed using calipers (e.g. skin nodules). For the case of skin lesions, documentation by colour photography including a ruler to estimate the size of the lesion is recommended. If feasible, imaging is preferred.
- **Chest X-ray.** Chest CT is preferred over chest X-ray, particularly when progression is an important endpoint, since CT is more sensitive than X-ray, particularly in identifying new lesions. However, lesions ≥ 20 mm on chest X-ray may be considered measurable if they are clearly defined and surrounded by aerated lung.
- **CT, MRI.** CT is the best currently available and reproducible method to measure lesions selected for response assessment. This guideline has defined measurability of lesions on CT scan based on the assumption that CT slice thickness is 5 mm or less. When CT scans have slice thickness greater than 5 mm, the minimum size for a measurable lesion should be twice the slice thickness. MRI is also acceptable in certain situations (e.g. for body scans). While PET scans are not considered adequate to measure lesions, PET-CT scans may be used providing that the measures are obtained from the CT scan and the CT scan is of identical diagnostic quality to a diagnostic CT (with IV and oral contrast).
- **Ultrasound.** Ultrasound is not useful in assessment of lesion size and should not be used as a method of measurement. If new lesions are identified by ultrasound in the course of the study, confirmation by CT should be obtained.
- **Endoscopy, Laparoscopy**. The utilization of these techniques for objective tumor evaluation is not advised. However, they can be useful to confirm complete pathological response when biopsies are obtained or to determine relapse in trials where recurrence following complete response or surgical resection is an endpoint.
- **Tumor Markers**. Tumor markers alone cannot be used to assess objective tumor response. If markers are initially above the upper normal limit, however, they must normalize for a patient to be considered in complete response.
- **Cytology, Histology**. These techniques can be used to differentiate between PR and CR in rare cases if required by protocol (for example, residual lesions in tumor types such as germ cell tumors, where known residual benign tumors can remain). When effusions are known

to be a potential adverse effect of treatment (e.g. with certain taxane compounds or angiogenesis inhibitors), the cytological confirmation of the neoplastic origin of any effusion that appears or worsens during treatment when the measurable tumor has met criteria for response or stable disease is advised to differentiate between response or stable disease and progressive disease.

8.3.2 Tumor response evaluation

All patients will have their BEST RESPONSE from the start of atezolizumab until the end of treatment classified as outlined below:

Complete or partial responses may be claimed only if the criteria for each are met at a subsequent time point at least 6 weeks later (table 2).

Complete Response (CR): disappearance of all *target* and *non-target* lesions and normalization of tumor markers. Pathological lymph nodes must have short axis measures < 10 mm (Note: continue to record the measurement even if < 10 mm and considered CR). Tumor markers must have normalized. Residual lesions (other than nodes < 10 mm) thought to be non-malignant should be further investigated (by cytology or PET scans) before CR can be accepted.

Partial Response (PR): at least a 30% decrease in the sum of measures (longest diameter for tumor lesions and short axis measure for nodes) of target lesions, taking as reference the baseline sum of diameters. Non target lesions must be non-PD.

Stable Disease (SD): Neither sufficient shrinkage to qualify for PR nor sufficient increase to qualify for PD taking as reference the smallest sum of diameters on study.

Progressive Disease (PD): at least a 20% increase in the sum of diameters of measured lesions taking as references the smallest sum of diameters recorded on study (including baseline) AND an absolute increase of ≥ 5 mm. Appearance of new lesions will also constitute PD (including lesions in previously unassessed areas). In exceptional circumstances, unequivocal progression of non-target disease may be accepted as evidence of disease progression, where the overall tumor burden has increased sufficiently to merit discontinuation of treatment, for example where the tumor burden appears to have increased by at least 73% in volume (which is the increase in volume when all dimensions of a single lesion increase by 20%). Modest increases in the size of one or more non-target lesions are NOT considered unequivocal progression. If the evidence of PD is equivocal (target or non-target), treatment may continue until the next assessment, but on further documentation, the earlier date must be used.

Table 2. Integration of target, non-target and new lesions into response assessment

Target Lesions	Non-Target Lesions	New Lesions	Overall Response	Best Response for this category also requires
Patients with Target lesions ± non target lesions				
CR	CR	No	CR	Normalization of tumor markers, tumor nodes < 10 mm
CR	Non-CR/Non-PD	No	PR	
CR	Not all evaluated	No	PR	
PR	Non-PD/ not all evaluated	No	PR	

SD	Non-PD/ not all evaluated	No	SD	documented at least once ≥ 8
Not all evaluated	Non-PD	No	NE	
PD	Any	Any	PD	
Any	PD	Any	PD	
Any	Any	Yes	PD	
Patients with Non target lesions ONLY				
No Target	CR	No	CR	Normalization of tumor markers, all tumor nodes < 10 mm
No Target	Non-CR/non-PD	No	Non-CR/ non-PD	
No Target	Not all evaluated	No	NE	
No Target	Unequivocal PD	Any	PD	
No Target	Any	Yes	PD	
Note: Patients with a global deterioration of health status requiring discontinuation of treatment without objective evidence of disease progression [or evidence of unequivocal disease progression] at that time should be reported as *"symptomatic deterioration"*. This is a reason for stopping therapy, but is NOT objective PD. Every effort should be made to document the objective progression even after discontinuation of treatment.				

Complete or partial responses may be claimed only if the criteria for each are met at a subsequent time point **at least 6 weeks later**. The best overall response can be interpreted as below:

Table 3. Best overall response

**Response: First time point**	**Subsequent time point**	**BEST overall response**	**Also requires**
CR	CR	CR	Normalization of tumor markers, tumor nodes < 10 mm
CR	PR	SD, PD or PR (see comment*)	
CR	SD	SD provided minimum criteria for SD duration met, otherwise, PD	
CR	PD	SD provided minimum criteria for SD duration met, otherwise, PD	
CR	NE	SD provided minimum criteria for SD duration met, otherwise NE	

PR	CR	PR	
PR	PR	PR	
PR	SD	SD	
PR	PD	SD provided minimum criteria for SD duration met, otherwise, PD	
PR	NE	SD provided minimum criteria for SD duration met, otherwise NE	
NE	NE	NE	
* may consider PR providing initial "CR" likely PR on subsequent review – then original CR should be corrected. Recurrence of lesion after true CR is PD.			

9 Statistical considerations

9.1 Statistical design

This is an investigator-initiated single arm phase II study to investigate the activity of atezolizumab in combination with carboplatin in metastatic ILC. In order to minimize the expected number of patients treated in the event that the regimen proves to be very disappointing or very successful, a two-stage design will be used for patient accrual.[22]

9.2 Sample size

For metastatic ILC patients who are refractory to endocrine treatment, no first or second line 'standard' therapies have been defined. Frequently used anticancer agents are capecitabine, or a taxane. The median PFS reached with these therapies typically lies between 2-4 months, but limited data are available. [25, 26] For this study, it is determined that a regimen that is likely to yield a proportion of patients that is free of progression at 24 weeks of more than 25% should be further explored in a randomized study.

Simon's two-stage (minimax) design will be used.[22] The null hypothesis that the true proportion of patients that is free of progression at 24 weeks is <= 10% will be tested against a one-sided alternative of at least 25%. In the first stage, 22 patients will be accrued. If there are 2 or fewer patients free of progression at 24 weeks in these 22 patients, the study will be stopped or another immunomodulatory drug will be added (agent to be determined). Otherwise, 18 additional patients will be accrued for a total of 40. The null hypothesis will be rejected if 8 or more patients out of those 40 are free of progression at 24 weeks. This design yields a type I error rate 0.04 and power of 80% when the true proportion of patients free of progression at 24 weeks is 25%.

In conclusion, in the run-in phase I part six patients will be included (3+3). In the first stage 22 (including the first n=6 from the run-in phase I part) patients will be included. If 3 or more patients are free of progression at 6 months (see statistics section below), an additional 18 subjects will be entered. Total= 22+18=40. For evaluation of efficacy according to the Simon's two-stage design we will use all patients who received at least 1 cycle of atezolizumab (per protocol population).

9.3 Accrual and duration of study

At least 40 patients will be included. The trial will open at the Netherlands Cancer Institute plus 3 academic hospitals in the Netherlands. We estimate that the Netherlands Cancer Institute will accrual 20 patients in two years' time. And for the other centers we expect to include 10 patients/center in two years' time. The estimated duration of the study is two years.

9.4 Statistical analysis populations

The following analysis populations will be used for the analysis of the trial:

- **Per protocol population:** All patients who have received at least one dose of atezolizumab.
- **Safety population:** All patients who have started on carboplatin.

9.5. Statistical methods

Efficacy analyses will be done using the **Per protocol population**. Safety analyses will be performed on the **Safety population**.

- Frequency tables will be tabulated (by treatment group or otherwise) for all categorical variables by the levels of the variables. Continuous variables (for example age, dose etc) are presented using the

median and range (minimum, maximum) or mean (variance) depending whether the distribution appears symmetrical.

- Baseline demographic characteristics of the **Per protocol population** will be summarized according to common reporting standards (following CONSORT-guidelines 2010, where applicable). Proportions will be presented together with 85% as well as 95% confidence intervals (CIs).
- Description of number of cycles of therapy and dose modifications for both the induction treatment as well as the treatment with atezolizumab (**Per protocol population**)
- Activity of atezolizumab plus carboplatin will be studied in the **Per protocol population** as described above by calculating the proportion of patients free of progression at 24 weeks (PFS according to RECIST (primary endpoint)). Spiderplots and swimmerplots will be used to illustrate the magnitude and duration of response.
- Activity of atezolizumab will be studied in the **Per protocol population** as described above by calculating ORR and clinical benefit (secondary endpoints) as defined by RECIST. Proportions will be presented together with 85% as well as 95% confidence intervals (CIs).
- Safety analyses will be performed on the **Safety population**. The worst toxicity grade over all cycles according to the CTCAE criteria version 4.03 will be displayed.
- For exploratory analyses, means will be compared using either student t-test if validity conditions are fulfilled or using non parametric Wilcoxon-Mann-Whitney tests. Proportions will be compared using either Chi2 statistics or Fisher's exact test depending on validity conditions.

9.6 Stopping rules

See dose-limiting toxicity at section 4.

10 Translational research

To facilitate development of innovative immunotherapy approaches there is a need to develop and validate tools to identify patients who can benefit from immunotherapy. Despite substantial effort, it is not yet known which parameters of antitumor immunity to measure and which assays are optimal for those measurements. Consequently, at the time of writing of the protocol, it is unknown which assays, validation techniques and cut-offs will be used for the final analyses.

Secondary research objective:

- Proportion of patients free of progression (RECIST 1.1[16]) at 6 months in the IR-profile subgroup vs the non-IR-subgroup as defined by gene expression profiling. See section on gene expression profiling below.

Translational research objectives (Exploratory objectives)

- Analyze the increase in immunogenicity after immune response induction treatment using carboplatin using pre-treatment biopsies/blood samples and biopsies/blood samples taken after 2 cycles of carboplatin before the start of atezolizumab. Potential markers to assess immunogenicity: TILs, PD-L1, CD8, FOXP3, CD68 (IHC), changes in gene expression with an emphasize on IFNγ-related gene signatures [18, 19, 21], serum cytokine levels, proportion of effector T cells and myeloid subpopulations in peripheral blood [20] (in collaboration with Schumacher and the Visser labs). Given that certain potentially highly important myeloid immune cell subset might not be viable after biobanking, we will perform flow cytometry within a couple of hours after blood draw (de Visser lab. 'Breast Immuno Flow').
- Comprehensive analyses of responding and non-responding cases using potential predictive markers such as but not limited to TILs, PD-L1 (according to the algorithm of Roche diagnostics), CD8, CD68, number of neoantigens, IFNγ-related gene signatures and serum LDH
- Exploring the role of circulating tumor DNA (ctDNA) cell free (cfDNA) during the discovery of potential predictive biomarkers (Erasmus MC). See section on ctDNA and cfDNA below.
- Exploring the role of gut microbiota composition and response to immunotherapy. See section on gut microbiome below.
- Biobanking of tumor tissues (frozen and FFPE), serum and whole blood. All collected at baseline, after induction/before start atezolizumab cycle 1 and before cycle 3, 5 and 7 for the purpose of retrospective analyses of candidate biomarkers or experiments to gain insight into response to immunotherapy in ILCs. See section on biobanking below.

Gene expression profiling

Recently, at our institute, a large scale genomic analysis of ILC identified an **immune related (IR) subtype** within ILCs characterized by mRNA up-regulation of CD4, CD8, PD-L1, PD1 and CTLA4 and greater sensitivity to DNA-damaging agents. [9] In line with this, researchers from The Cancer Genome Atlas (TCGA) have shown that immune-related genes (interleukins, chemokines, MHC complex, IDO1 and IFNy) are highly expressed in a subset of ILC tumors. [10] This suggests that this IR-subtype that it is more likely that in these tumors a pre-existing anti-cancer T cell response is present and that PD1-blockade might be more effective in these cases. To test this hypothesis we conducted this exploratory phase II GELATO study. Within this study, gene expression profiling using frozen biopsies taken at baseline will be conducted using Agilent arrays at the laboratory of Agendia BV. To classify a tumor into IR-related or non-IR-related, the genelist generated by the RATHER consortium will be used. [9]

ctDNA and cfDNA

Mutations and rearrangements provide promising markers for patient-specific disease monitoring in cell free DNA (cfDNA) present in the circulation. This cell free DNA contains tumor DNA (ctDNA) which represents a very attractive and sensitive option to establish mutational changes occurring in tumor cells in a minimal invasive manner. For instance in patients with metastatic breast cancer mutations in the estrogen receptor (ESR1) are rarely detected in primary tumors but are frequently reported in plasma from patients with metastatic disease with acquired resistance to aromatase inhibitors. In the Erasmus MC, ample experience exists with regard to sensitive mutation profiling of ctDNA using digital PCR (see figure below) or targeted sequencing. Next to ctDNA, cfDNA can also contain DNA from various immune-related cells such as T-cells and genetic changes in these cells could also be monitored. Genetic aberration in ctDNA which may provide early markers for immune response and may improve insight in combination strategies, may be monitored during disease progression and may thus guide treatment decisions in future studies

Primary endpoint of this side study: assessment of patient-specific mutations in E-cadherin, the driver in ILC, as an early response marker to the combination treatment.

Secondary endpoint(s) of the side study: i) assessment of mutations in E-cadherin as a marker to monitor disease progression, ii) assessment of the changing landscape of somatic mutations in ctDNA (by targeted or genome-wide NGS analysis) during combination treatment, iii) exploratory analysis of TCR rearrangements in cfDNA at progression on the combination treatment.

Gut microbiome

Gut microbiota play a role in immune system development and can affect the occurrence of autoimmunity. Recently two independent mouse studies provide strong evidence for the role of stool microbiota (i.e. intestinal microbes) in response and resistance to checkpoint blockade. [45, 46] For now, additional studies on patient populations are warranted to understand how the microbiome of our cancer patients is involved in response to T cell based therapies. Unraveling the gut microbiome might lead to predictive biomarkers for response and/or toxicity and can form the basis for, ultimately, manipulation of the microbiome to favor response in immunotherapy-treated cancer patients. [47] Preliminary data presented at the recent ASCO-SITC meeting suggest that rRNA sequencing of the stool of melanoma patients treated with anti-PD1 is correlated with response to anti-PD1: patients with a more diverse makeup of the gut microbiome were more likely to respond to the treatment. [48]

In collaboration with the group of professor Max Nieuwdorp (AMC, Amsterdam) who is an expert on gut microbiome, metabolism and diabetes, we will analyze the gut microbiome using stool samples (collected at baseline, before start atezolizumab and after 2 cycles of atezolizumab). The Nieuwdorp lab uses the same pipeline as the Backhed lab that is renowned for their work on sequencing of gut microbiota. [49] Fecal DNA will be extracted via mechanical cell disruption by repeated bead beating for which it has been shown that it results in optimal lysis of bacteria. [50] 16S rRNA gene fragments will be sequenced using Illumina's MiSeq platform. [51] Given the relative small number of patients and the abundant information that can be extracted from the microbiome in the stool, this side study has to be considered exploratory.

Systemic immune suppression

Emerging data in the field of clinical melanoma research show that circulating immunosuppressive cells/factors, including neutrophils, are associated with poor outcome after immunotherapy. This is supported by our recent data that systemic activation of the γδ T cell/IL17/neutrophil cascade interferes with successful anti-tumor immunity in our breast cancer mouse model. [20] Moreover, via inhibition of this cascade we were able to downregulate the T-cell suppressive phenotype of neutrophils. Hence, these data provide not only a possible new target for immunotherapeutic intervention, but can also lead to a biomarker that can aid in selecting ILC patients for immunotherapy. For the patients treated at the NKI, we will process fresh blood and perform 15-parameter (intracellular) flow cytometry to assess the composition and phenotype of innate and adaptive immune cells with

a focus on neutrophil subtypes, including iNOS production, and γδ T cells. Fresh blood (1 tube) will be processed at baseline, before start atezolizumab and after 2 cycles of atezolizumab.

Biobanking

To enable 1) experiments that will contribute to the understanding of the anti-cancer effector T cell response in ILC as well as relevant immune suppressive mechanisms, and 2) the analysis of a putative biomarkers using different sources the following samples will be biobanked:

<u>1. Tumorbiopsies (3x16G) at baseline, before start atezolizumab(=after 2 cycles of carboplatin) and after 2 cycles of atezolizumab</u>

1.1 FFPE (formalin fixed paraffin embedded) tissue (1 biopsy), to analyze:

- percentage of TILs in the tumor[52, 53], percentage of TILs in the stroma [52, 53],
- multiplex immunofluorescence: percentage and location membranous PD-L1 expression [54], number and location of FOXP3+ Tregs [55, 56], the ratio of CD8/CD4+ T cells [52, 53], number and location of macrophages and changes in myeloid derived suppressor cells (MDSCs). Pre- and post-treatment samples will be compared. Absolute and relative changes will be described.

1.2 Fresh Frozen Tissue (2 biopsies), to analyze:

- DNA for exome-sequencing for the detection of the mutational load, neo-antigens [57] and specific mutations associated with response to atezolizumab in ILCs. It also allows the evaluation of changes in the intratumoral TCR repertoire.
- RNA for gene expression: Compare patterns of gene expression signatures in pre- and post-treatment samples. Candidate gene expression signatures to evaluate are the interferon-gamma/CD8 gene set as presented by Herbst and further developed by Fehrenbacher and colleagues. This signature is clearly associated with response to atezolizumab in NSCLC [18, 19] Other gene signatures of interest: IgG/lymphocyte-specific kinase metagene [58] as well as the immune-relevant metagenes. [59, 60] We hypothesize that immune-related gene expression signatures will change significantly after a) induction treatment with carboplatin, as well as after b) start of atezolizumab. Exploratory unsupervised analyses will be performed. Evaluation of immune cytolytic activity 'CYT' [61] will yield insight in the magnitude of immunoediting in tumors.

<u>2. Peripheral blood at baseline, before cycle 1, 3, 5, and 7 (before cycles 5 and 7 optional for other centers than the NKI) and at progression (optional), stored as</u>

- PBMCs (peripheral blood mononuclear cells) for changes in T cell subsets, neo-antigen specific T cells, TCR repertoire (10 tubes at baseline, 5 tubes at follow up)
- At baseline only: whole blood for isolation of germline DNA (can be used as a reference for tumor exome sequencing) (only 1 tube at baseline)
- Serum for changes in cytokines, chemokines and growth factors (1 tube)
- Plasma for changes in circulating tumor DNA and platelets for mRNA analyses of tumor-educated platelets (1 tube)

11 Safety reporting

11.1 Section 10 WMO event

In accordance to section 10, subsection 4, of the WMO, the investigator will inform the patients and the reviewing accredited METC if anything occurs, on the basis of which it appears that the disadvantages of participation may be significantly greater than was foreseen in the research proposal. The study will be suspended pending further review by the accredited METC, except insofar as suspension would jeopardize the patients' health. The investigator will take care all patients are kept informed.

11.2 Adverse and serious adverse events

Adverse events are defined as any undesirable experience occurring to a patient during a clinical trial, whether or not considered related to the investigational drug (i.e. can also be related to the core biopsies). All adverse events reported spontaneously by the patient or observed by the investigator or his staff will be recorded.
A serious adverse event is any untoward medical occurrence or effect that at any dose

- results in death;
- is life threatening (at the time of the event);
- requires hospitalization or prolongation of existing inpatients' hospitalization;
- results in persistent or significant disability or incapacity;
- is a congenital anomaly or birth defect;
- is a new event of the trial likely to affect the safety of the patients, such as an unexpected outcome
- of an adverse reaction.

Life threatening: the term 'life threatening' in the definition of 'serious' refers to an adverse event in which the subject was at risk of death at the time of the event. It does not refer to an adverse event which hypothetically might have caused death if it were more severe.

Hospitalization: any adverse event leading to hospitalization or prolongation of hospitalization will be considered as 'serious', UNLESS at least one of the following exceptions are met:

- the admission is pre-planned (e.g. elective or scheduled surgery arranged prior to the start of the study, documented in the patient's file);
- prolonged hospitalization for technical, practical or social reasons, in the absence of an adverse event.

All SAEs will be reported to the accredited METC that approved the protocol, according to the requirements of that METC.

Adverse Events of special interest will be treated according to the recommendations for management of a specific Adverse Event as described in section 6 of this protocol.

<u>Potential Drug Induced Liver Injury (DILI)</u>

Wherever possible, timely confirmation of initial liver-related laboratory abnormalities should occur prior to the reporting of a potential DILI event. All occurrences of potential DILIs, meeting the defined criteria, must be reported as SAEs. Potential drug induced liver injury is defined as:

1) ALT or AST elevation > 3 times upper limit of normal (ULN)
   AND
2) Total bilirubin > 2 times ULN, without initial findings of cholestasis (elevated serum alkaline phosphatase)
   AND

3) No other immediately apparent possible causes of AST/ALT elevation and hyperbilirubinemia, including, but not limited to, viral hepatitis, pre-existing chronic or acute liver disease, or the administration of other drug(s) known to be hepatotoxic.

11.3 Suspected unexpected serious adverse reactions (SUSAR)

SUSARs are unexpected adverse reactions with a suspected relationship to the investigational medicinal product. Unexpected adverse reactions are adverse reactions, of which the nature, or severity, is not consistent with the applicable product information (e.g. Summary of Product Characteristics (SPC)).

11.4 Recording of AEs

At each contact with the patient, the study personnel must seek information on (serious) adverse events by specific questioning and, as appropriate, by examination. Information on all (serious) adverse events should be recorded promptly in the patient's medical records. This information must be as complete as possible and preferably include start and stop date of the event, CTCAE 4.03 grading (see below) and the relation to study medication. At a later moment this information will be transferred from the medical records to the Case Report Forms. All adverse events starting after the administration of the first study medication and until the patient's final study visit must be recorded. The clinical course of each event must be followed until resolution or stabilization. Any serious adverse event which occurs within 30 days after the study period and is considered to be possibly related to study treatment or study participation should be recorded as well.

11.5 Recording of SAEs

Besides recording in the patient's medical record, for each serious adverse event, the Serious Adverse Event Form must be completed. On the SAE form all clearly related signs, symptoms and abnormal diagnostic procedures must be recorded as a single diagnosis. The component parts of the diagnosis may be listed for verification.

The following definitions will be used to assess causality:

Not related: The clinical adverse event is definitely unrelated to the study drug (e.g., does not follow a reasonable temporal sequence from study drug administration, present prior to receiving study medication, etc.).

Unlikely: The study drug is not likely to have had reasonable association with the observed experience; however, relationship cannot be definitely excluded.

Possible: The connection with study drug administration appears unlikely, but cannot be excluded with certainty (e.g., follows a reasonable temporal sequence from drug administration, may be related to known characteristics of the patients' clinical state or other modes of therapy administered to the patient, etc.).

Probable: The clinical adverse event appears related to the study drug with a high degree of certainty (e.g., follows a reasonable temporal sequence from drug administration and abates upon discontinuation of the drug, cannot be reasonably explained by known characteristics of the patient's clinical state or other modes of therapy administered to the patient, etc.).

Definite: the event follows a reasonable temporal sequence from the time of drug administration, and follows a known response pattern to the study drug, cannot be reasonably explained by other factors such as the patient's condition, therapeutic interventions or concomitant drugs; AND occurs immediately following study drug administration, improves on stopping the drug, or reappears on re-exposure

The intensity of an adverse event will be graded according to the NCI Common Toxicity Criteria (NCICTCAE, Version 4.03, final 7 April 2009; see http://evs.nci.nih.gov/ftp1/CTCAE/About.html or Appendix A). Adverse events that can not be graded using the NCI Common Toxicity Criteria will be graded as mild (asymptomatic), moderate (symptomatic but not interfering significantly with function) or severe (causing significant interference with function).

11.6 Reporting of SAEs

All serious adverse events (including pregnancy) starting after administration of the first study medication (induction treatment) and within 30 days after the last protocol treatment administration, whether considered by the investigator to be related to study treatment or not, must be medically well documented and reported to the AVL Safety Desk within 24 hours or, at the latest, on the following working day. The report must be made by email: drugsafety@nki.nl or fax: 0031 (0)20-5122679.

Reporting must be done using the Serious Adverse Event Form of the study. The forms will be filed at the NKI-AVL Safety Desk. The Safety Officer must notify the study coordinators within 1 working day of any serious adverse event (as defined above) experienced by a patient.

All serious adverse events must be followed up until resolution or stabilization, and this information must be reported to the NKI-AVL Safety Desk as soon as it becomes available, using a follow-up Serious Adverse Event Form. This form will be signed by the investigator and filed together with the initial report.

In addition, serious adverse events must be reported by the NKI-AVL Safety Desk to regulatory authorities according to the definitions and timelines specified in the local laws and regulations. All SAE's will be reported once yearly, as described in the section " Annual safety report". SAE's for this multicenter oncological study will not be reported through the web portal ToetsingOnline to the METC.

Serious adverse events occurring more than 30 days after the last study medication/treatment will NOT be reported unless the investigator feels that the event may have been caused by the study treatment or a protocol procedure. Study-specific clinical outcomes of death because of disease progression are exempt from serious adverse event reporting, unless the investigator deems them related to use of the study drug.

When required by contractual obligations, the NKI will report SAEs to atezolizumab to Roche.

11.7 Reporting of SUSARs

The study coordinator reviews any related SAE for expectedness. A SAE that meets the SUSAR criteria will be classified as SUSAR:

1. the event must be serious;

there must be a certain degree of probability that the event is a harmful and an undesirable reaction to the medicinal product under investigation, regardless of the administered dose;

2. the adverse reaction must be unexpected, that is to say, the nature and

severity of the adverse reaction are not in agreement with the product information as recorded in:

- Summary of Product Characteristics (SPC) for an authorised medicinal product;
- Investigator's Brochure for an unauthorised medicinal product.

The NKI is the sponsor of this study. The NKI-AVL Safety Desk will represent the NKI for the sponsor task of reporting safety data according to the WMO requirements. The NKI Data Center personnel will notify the study coordinator of any serious adverse event reported. The study coordinator must notify the AVL Safety Desk of the existence of this SUSAR within 24 hours using the SUSAR qualification form.

The AVL Safety Desk will report expedited the SUSAR through the web portal ToetsingOnline to the METC. The expedited reporting will occur not later than 15 days after the AVL Safety Desk has first knowledge of the adverse reactions. For fatal or life threatening cases the term will be maximal 7 days for a preliminary report with another 8 days

for completion of the report.
The expedited reporting of SUSARs through the web portal ToetsingOnline is sufficient as notification to the competent authority.

11.8 Annual safety report

In addition to the expedited reporting of SUSARs, the sponsor will submit, once a year throughout the clinical trial, a safety report to the accredited METC, competent authority, Medicine Evaluation Board and competent authorities of the concerned Member States.
This safety report consists of:

- a list of all suspected (unexpected or expected) serious adverse reactions, along with an aggregated summary table of all reported serious adverse reactions, ordered by organ system, per study;
- a report concerning the safety of the subjects, consisting of a complete safety analysis and an evaluation of the balance between the efficacy and the harmfulness of the medicine under investigation.

12 Administrative aspects and monitoring

12.1 Subject identification

Following enrolment, using the ALEA® registration package, a patient will receive a sequential identification number within the study. This number will be used for identification of the patients and will be the key on all electronic case record forms (eCRFs). Data and patient material will be handled confidentially and if possible anonymously. To be able to trace data or material to an individual subject, each center will keep a subject identification number list.
The following persons will have access to the source data: members of the local study team, monitors and auditors from or on behalf of the sponsor, representatives from the Inspectie voor de Volksgezondheid and inspectors from foreign health authorities. The handling of personal data complies with the Dutch Personal Data Protection Act (in Dutch: De Wet Bescherming Persoonsgegevens, WBP).
The list of staff members authorized to complete case report forms must be documented on the Delegation Log of the study and a copy should be sent to the Central Data Centre by the responsible investigator before the start of the study.
The investigator will retain all pertinent information for a period of at least 15 years from study completion. The investigator will be responsible for retaining sufficient information about each patient (e.g. name, address, phone number, and identity in the study) so that regulatory agencies or participating investigators may access this information should the need to do so arise. These records should be retained in a confidential manner for as long as legally mandated according to local requirements.

12.2 Storage of patient material

Patient material will be stored at the NKI. The tissue will be stored at the core facility molecular pathology and biobanking (CFMPB). Material that is not used for current translational research will be stored for future research.

12.3 Registration of patients

Patient registration will only be accepted from authorized investigators or through their authorized data manager or authorized staff member. A patient can be registered only after verification of eligibility. During the registration procedure eligibility criteria and patients' informed consent are checked. Registration will be done through the ALEA® registration package.

12.4 Data management

All data that are relevant for the study will be collected on eCRFs developed in ALEA® by the data center of the NKI-AVL. The system can be accessed via internet to include data directly in the Data Centre Servers. The NKI-AVL Datacenter will supply accounts to the local data manager to enter data into the system. Checks will be incorporated into the eCRF system to prompt queries at the moment that data is entered facilitating the work of the local data manager who could quickly correct errors. Additional checks will be programmed using statistical programs with the goal of obtaining a clean file.
The time between the patient's visit and completion of eCRF pages should be kept to a reasonable minimum allowing answering the questions posed by the study. In all cases it remains the responsibility of the investigator to check and validate the data after verifying that they are completed and filled out correctly. If information is not known, this must be clearly indicated.

12.5 Monitoring and Quality Assurance

The study will be monitored according to ICH GCP. This study will be considered as a **medium** risk study. Site monitoring will be performed by an independent Clinical Research Monitor or the person to whom the monitoring tasks have been delegated.
Amongst others the following will be reviewed:

- Compliance with the protocol, ICH-GCP and all applicable regulatory requirements.

- Informed Consent
- Source Data Verification
- Investigator Study File
- (Serious) Adverse Events / SUSAR
- Drug accountability, if applicable

A monitoring plan specific to the study and describing the nature and frequency of the monitoring will be written by the appointed monitor and approved by the Principal Investigator and the Head of the Data Centre.

Data from all patients will also be centrally checked at the Data Centre. Through central monitoring of the data collected, the Data Centre will be able to detect outliers or apparently spurious data. When persistent irregularities or protocol violations are detected, the Data Centre will inform the local investigator (and Principal Investigators) and queries will be sent to the local Data Manager

12.6 Protocol Approval and Amendment

12.6.1 Protocol approval

The investigator will submit the final protocol, patient information sheet, informed consent form and other relevant documents (e.g. diary) for independent review to the accredited METC and for a safety evaluation to the competent authority (CCMO). For this study the Medisch Ethische Toetsings Comissie (METC AVL) of the NKI will be the accredited METC. Subject recruitment will not start until satisfactory evidence of ethical approval is given to the responsible investigator and an email report with a statement of no objection has been received from the CCMO.

12.6.2 Amendments

Amendments are changes made to the protocol after a favorable opinion by the accredited METC and the CCMO has been given.

A ‘substantial amendment’ is defined as an amendment to the terms of the METC application, or to the protocol or any other supporting documentation, that is likely to affect to a significant degree:

- the safety or physical or mental integrity of the subjects of the trial;
- the scientific value of the trial;
- the conduct or management of the trial; or
- the quality or safety of any intervention used in the trial

All substantial amendments will be submitted to the METC that gave a favorable opinion to an earlier version of the protocol and the competent authority (CCMO).

Non-substantial amendments will not be submitted to the accredited METC and CCMO, but will be recorded and filed by the investigator.

Modifications of the protocol will not be implemented without prior approval by the accredited METC and an email with a statement of no objection from the CCMO of a substantial amendment. However, the investigator may implement a deviation from, or change of the protocol to eliminate immediate hazard(s) to the trial patients without prior METC approval. If appropriate the deviation or change will be included in an amendment and submitted to the accredited METC and CCMO for approval.

13 Ethical considerations

13.1 Patient protection

The responsible investigator will ensure that this study is conducted in agreement with either the Declaration of Helsinki (ref:http://www.wma.net/en/30publications/10policies/b3/index.html) or the laws and regulations of the country, whichever provides the greatest protection of the patient.

The protocol has been written, and the study will be conducted according to the ICH Harmonized Tripartite Guideline for Good Clinical Practice (ref:http://www.ifpma.org/pdfifpma/e6.pdf). The protocol will be approved by the local, regional or national ethics committees.

13.2 Informed consent

All patients will be informed of the aims of the study, the possible adverse events, and the procedures and possible hazards to which he/she will be exposed. They will be informed as to the strict confidentiality of their patient data, but that their medical records may be reviewed for trial purposes by authorized individuals other than their treating physician.

13.3 Withdrawal criteria Patients are free to withdraw at any time without giving reasons and without prejudice to their subsequent care.

13.4 Insurance of liabilities

The principal investigator has a liability insurance, which is in accordance with article 7, subsection 6 of the WMO.

The principal investigator also has insurance, which is in accordance with the legal requirements in the Netherlands (Article 7 WMO and the Measure regarding Compulsory Insurance for Clinical Research in Humans of 23th June 2003). This insurance provides cover for damage to research subjects through injury or death caused by the study:

1. € 650.000,- (i.e. five hundred and fifty thousand Euro) for death or injury for each subject who participates in the research;
2. € 5.000.000,- (i.e. five million Euro) for death or injury for all subjects who participate in the research;
3. € 7.500.000,- (i.e. seven million five hundred thousand Euro) for the total damage incurred by the organization for all damage disclosed by scientific research for the Sponsor as ‘verrichter’ in the meaning of said Act in each year of insurance coverage.

The insurance applies to the damage that becomes apparent during the study or within 4 years after the end of the study.

Appendix A: NCI CTCAE 4.03

The grading of adverse events and/or adverse drug reactions will be reported according to the NCI Common Terminology Criteria for Adverse Events, CTCAE version 4.03, published June 14, 2010 (NIH Publication No. 09-5410). The complete document (194 pages) can be reviewed and downloaded from the following internet site:

http://evs.nci.nih.gov/ftp1/CTCAE/About.html

Appendix B: WHO Performance Status scale

Performance Status Criteria

**WHO Performance Status Scale**	
**Grade**	Descriptions
0	Normal activity. Fully active, able to carry on all pre-disease performance without restriction.
1	Symptoms, but ambulatory. Restricted in physically strenuous activity, but ambulatory and able to carry out work of a light or sedentary nature (e.g., light housework, office work)
2	In bed <50% of the time. Ambulatory and capable of all self-care, but unable to carry out any work activities. Up and about more than 50% of waking hours.
3	In bed >50% of the time. Capable of only limited self-care, confined in bed or chair more than 50% of waking hours.
4	100% bedridden. Completely disabled. Cannot carry on any self-care. Totally confined to bed or chair
5	Deceased.

References

1. Brahmer, J.R., et al., *Safety and Activity of Anti–PD-L1 Antibody in Patients with Advanced Cancer.* New England Journal of Medicine, 2012. **366**(26): p. 2455-2465.
2. Powles, T., et al., *MPDL3280A (anti-PD-L1) treatment leads to clinical activity in metastatic bladder cancer.* Nature, 2014. **515**(7528): p. 558-562.
3. Topalian, S.L., et al., *Survival, Durable Tumor Remission, and Long-Term Safety in Patients With Advanced Melanoma Receiving Nivolumab.* Journal of Clinical Oncology, 2014. **32**(10): p. 1020-1030.
4. Emens, L., *P01-6. Inhibition of PDL1 by MDPL3280A leads to clinical responses in patients with metastatic triple negative breast cancer*, in *San Antonio Breast Cancer Symposium*. 2014.
5. Nanda, R., *S1-09. A phase Ib study of pembrolizumab in patients with advanced triple negative breast cancer*, in *San Antonio Breast Cancer Symposium*. 2014.
6. Dirix, L. *[S1-04] Avelumab (MSB0010718C), an anti-PD-L1 antibody, in patients with locally advanced or metastatic breast cancer: A phase Ib JAVELIN solid tumor trial*. in *San Antonio Breast Cancer Symposium*. 2015.
7. Rugo H, D.J., Im SA et al. . *Preliminary efficacy and safety of pembrolizumab (MK-3475) in patients with PD-L1-positive, ER-positive (ER+)/HER-2 negative breast cancer enrolled in Keynote 028.* . in *San Antonio Breast Cancer Symposium*. 2015.
8. Desmedt, C., et al., *Genomic Characterization of Primary Invasive Lobular Breast Cancer.* Journal of Clinical Oncology, 2016. **34**(16): p. 1872-1881.
9. Michaut, M., et al., *Integration of genomic, transcriptomic and proteomic data identifies two biologically distinct subtypes of invasive lobular breast cancer.* Scientific Reports, 2016. **6**: p. 18517.
10. Ciriello, G., et al., *Comprehensive Molecular Portraits of Invasive Lobular Breast Cancer.* Cell. **163**(2): p. 506-519.
11. Galluzzi, L., et al., *The secret ally: immunostimulation by anticancer drugs.* Nat Rev Drug Discov, 2012. **11**(3): p. 215-233.
12. Kersten, K., C. Salvagno, and K.E. de Visser, *Exploiting the immunomodulatory properties of chemotherapeutic drugs to improve the success of cancer immunotherapy.* Frontiers in Immunology, 2015. **6**.
13. Lesterhuis, W.J., et al., *Platinum-based drugs disrupt STAT6-mediated suppression of immune responses against cancer in humans and mice.* The Journal of Clinical Investigation, 2011. **121**(8): p. 3100-3108.
14. van der Sluis, T.C., et al., *Vaccine-Induced Tumor Necrosis Factor–Producing T Cells Synergize with Cisplatin to Promote Tumor Cell Death.* Clinical Cancer Research, 2015. **21**(4): p. 781-794.
15. Doornebal, C.W., et al., *A Preclinical Mouse Model of Invasive Lobular Breast Cancer Metastasis.* Cancer Research, 2013. **73**(1): p. 353-363.
16. Wolchok, J.D., et al., *Guidelines for the Evaluation of Immune Therapy Activity in Solid Tumors: Immune-Related Response Criteria.* Clinical Cancer Research, 2009. **15**(23): p. 7412-7420.
17. Seymour, L., et al., *iRECIST: guidelines for response criteria for use in trials testing immunotherapeutics.* The Lancet Oncology. **18**(3): p. e143-e152.
18. Fehrenbacher, L., et al., *Atezolizumab versus docetaxel for patients with previously treated non-small-cell lung cancer (POPLAR): a multicentre, open-label, phase 2 randomised controlled trial.* The Lancet. **387**(10030): p. 1837-1846.
19. Herbst, R.S., et al., *Predictive correlates of response to the anti-PD-L1 antibody MPDL3280A in cancer patients.* Nature, 2014. **515**(7528): p. 563-567.
20. Coffelt, S.B., et al., *IL-17-producing [ggr][dgr] T cells and neutrophils conspire to promote breast cancer metastasis.* Nature, 2015. **522**(7556): p. 345-348.
21. Ji, R.-R., et al., *An immune-active tumor microenvironment favors clinical response to ipilimumab.* Cancer Immunology, Immunotherapy, 2012. **61**(7): p. 1019-1031.
22. Simon, R., *Optimal two-stage designs for phase II clinical trials.* Controlled Clinical Trials, 1989. **10**(1): p. 1-10.

23. von Minckwitz, G., et al., *Neoadjuvant carboplatin in patients with triple-negative and HER2-positive early breast cancer (GeparSixto; GBG 66): a randomised phase 2 trial.* The Lancet Oncology. **15**(7): p. 747-756.
24. Rizvi, N.A., et al., *Nivolumab in Combination With Platinum-Based Doublet Chemotherapy for First-Line Treatment of Advanced Non–Small-Cell Lung Cancer.* Journal of Clinical Oncology, 2016. **34**(25): p. 2969-2979.
25. Blum, J.L., et al., *Pooled analysis of individual patient data from capecitabine monotherapy clinical trials in locally advanced or metastatic breast cancer.* Breast Cancer Research and Treatment, 2012. **136**(3): p. 777-788.
26. Cortes, J., et al., *Eribulin monotherapy versus treatment of physician's choice in patients with metastatic breast cancer (EMBRACE): a phase 3 open-label randomised study.* The Lancet. **377**(9769): p. 914-923.
27. Lakhani, S.R., *WHO Classification of Tumors of the Breast*. 4th ed. 2012: IARS.
28. Iorfida, M., et al., *Invasive lobular breast cancer: subtypes and outcome.* Breast Cancer Research and Treatment, 2012. **133**(2): p. 713-723.
29. Pestalozzi, B.C., et al., *Distinct Clinical and Prognostic Features of Infiltrating Lobular Carcinoma of the Breast: Combined Results of 15 International Breast Cancer Study Group Clinical Trials.* Journal of Clinical Oncology, 2008. **26**(18): p. 3006-3014.
30. Adams, S. *[P2-11-06] Safety and clinical activity of atezolizumab (anti-PDL1) in combination with nab-paclitaxel in patients with metastatic triple-negative breast cancer*. in *San Antonio Breast Cancer Symposium*. 2015.
31. Emens, L., *Inhibition of PD-L1 by MPDL13280A leads to clinical activity in patients with metastatic triple-negative breast cancer (TNBC)*, in *AACR*. 2015.
32. Nanda, R., et al., *Pembrolizumab in Patients With Advanced Triple-Negative Breast Cancer: Phase Ib KEYNOTE-012 Study.* Journal of Clinical Oncology, 2016.
33. Arpino, G., et al., *Infiltrating lobular carcinoma of the breast: tumor characteristics and clinical outcome.* Breast Cancer Research, 2004. **6**(3): p. R149.
34. Eisenhauer, E.A., et al., *New response evaluation criteria in solid tumours: Revised RECIST guideline (version 1.1).* European Journal of Cancer. **45**(2): p. 228-247.
35. McDermott, D.F., et al., *Atezolizumab, an Anti-Programmed Death-Ligand 1 Antibody, in Metastatic Renal Cell Carcinoma: Long-Term Safety, Clinical Activity, and Immune Correlates From a Phase Ia Study.* J Clin Oncol, 2016. **34**(8): p. 833-42.
36. Herbst, R.S., et al., *Predictive correlates of response to the anti-PD-L1 antibody MPDL3280A in cancer patients.* Nature, 2014. **515**(7528): p. 563-7.
37. Sylviia Adams, J.D., Erika Hamilton, Paula Pohlmann, Sara Tolaney, Luciana Molinero, Xian He, Daniel Waterkamp, Roel Funke, John Powderly *Safety and clinical activity of atezolizumab (anti-PDL1) in combination with nab-paclitaxel in patients with metastatic triple-negative breast cancer* 2015.
38. L., K., *The resurgence of platinum based cancer chemotherapy. .* Nat Rev Cancer 2007. **7**: p. 573-584.
39. Hall MD, O.M., Shen DW et al. , *The role of cellular accumulation in determing sensitivity to platinum based chemotherapy* Annu Rev Pharmacol Toxicol 2008. **48**: p. 495-535.
40. EA, P., *Carboplatin in combination therapy for metastatic breast cancer* Oncologist 2004. **9**: p. 518-527.
41. Kaneno, R., et al., *Chemomodulation of human dendritic cell function by antineoplastic agents in low noncytotoxic concentrations.* Journal of Translational Medicine, 2009. **7**(1): p. 1-10.
42. Kerbel, R.S. and B.A. Kamen, *The anti-angiogenic basis of metronomic chemotherapy.* Nat Rev Cancer, 2004. **4**(6): p. 423-436.
43. Shurin, G.V., et al., *Chemotherapeutic Agents in Noncytotoxic Concentrations Increase Antigen Presentation by Dendritic Cells via an IL-12-Dependent Mechanism.* The Journal of Immunology, 2009. **183**(1): p. 137-144.
44. Hodi, F.S. *Evaluation of immune-related response criteria (irRC) in patients (pts) with advanced melanoma (MEL) treated with the anti-PD-1 monoclonal antibody MK-3475.* in *J Clin Oncol 32:5s, 2014 (suppl; abstr 3006^)*. 2014.

45. Sivan, A., et al., *Commensal Bifidobacterium promotes antitumor immunity and facilitates anti–PD-L1 efficacy.* Science, 2015. **350**(6264): p. 1084-1089.
46. Vétizou, M., et al., *Anticancer immunotherapy by CTLA-4 blockade relies on the gut microbiota.* Science, 2015. **350**(6264): p. 1079-1084.
47. Pitt, J.M., et al., *Fine-Tuning Cancer Immunotherapy: Optimizing the Gut Microbiome.* Cancer Research, 2016. **76**(16): p. 4602-4607.
48. V., G. *Association of diversity and composition of the gut microbiome with differential responses to PD-1 based therapy in patients with metastatic melanoma*. in *ASCO-SITC Clinical Immuno-Oncology Symposium*. 2017.
49. Ziętak, M., et al., *Altered Microbiota Contributes to Reduced Diet-Induced Obesity upon Cold Exposure.* Cell Metabolism, 2016. **23**(6): p. 1216-1223.
50. Salonen, A., et al., *Comparative analysis of fecal DNA extraction methods with phylogenetic microarray: Effective recovery of bacterial and archaeal DNA using mechanical cell lysis.* Journal of Microbiological Methods, 2010. **81**(2): p. 127-134.
51. Kozich, J.J., et al., *Development of a Dual-Index Sequencing Strategy and Curation Pipeline for Analyzing Amplicon Sequence Data on the MiSeq Illumina Sequencing Platform.* Applied and Environmental Microbiology, 2013. **79**(17): p. 5112-5120.
52. Loi, S., et al., *Prognostic and Predictive Value of Tumor-Infiltrating Lymphocytes in a Phase III Randomized Adjuvant Breast Cancer Trial in Node-Positive Breast Cancer Comparing the Addition of Docetaxel to Doxorubicin With Doxorubicin-Based Chemotherapy: BIG 02-98.* Journal of Clinical Oncology, 2013. **31**(7): p. 860-867.
53. Nielsen, D., Salgado, , *Guidelines of quantifying tumor ilfiltrating lymphocyes*. 2014: Ann Oncol.
54. Taube, J.M., et al., *Association of PD-1, PD-1 ligands, and other features of the tumor immune microenvironment with response to anti-PD-1 therapy.* Clinical Cancer Research, 2014.
55. Karasar, P. and G. Esendagli, *T helper responses are maintained by basal-like breast cancer cells and confer to immune modulation via upregulation of PD-1 ligands.* Breast Cancer Research and Treatment, 2014. **145**(3): p. 605-614.
56. Liu, F., et al., *CD8+ cytotoxic T cell and FOXP3+ regulatory T cell infiltration in relation to breast cancer survival and molecular subtypes.* Breast Cancer Research and Treatment, 2011. **130**(2): p. 645-655.
57. Schumacher, T.N. and R.D. Schreiber, *Neoantigens in cancer immunotherapy.* Science, 2015. **348**(6230): p. 69-74.
58. Zitvogel, L., O. Kepp, and G. Kroemer, *Immune parameters affecting the efficacy of chemotherapeutic regimens.* Nat Rev Clin Oncol, 2011. **8**(3): p. 151-160.
59. Rody, A., et al., *T-cell metagene predicts a favorable prognosis in estrogen receptor-negative and HER2-positive breast cancers.* Breast Cancer Research, 2009. **11**(2): p. R15.
60. Teschendorff, A., et al., *An immune response gene expression module identifies a good prognosis subtype in estrogen receptor negative breast cancer.* Genome Biology, 2007. **8**(8): p. R157.
61. Rooney, Michael S., et al., *Molecular and Genetic Properties of Tumors Associated with Local Immune Cytolytic Activity.* Cell, 2015. **160**(1–2): p. 48-61.
