# Supplementary material for: PD-L1 blockade in combination with carboplatin as immune induction in metastatic lobular breast cancer: the GELATO trial
Source: Nat Cancer. 2023 Apr 10;4(4):535–49. doi: 10.1038/s43018-023-00542-x (PMC10132987; doi:10.1038/s43018-023-00542-x)
Supplement: Supplementary file 2 — Reporting Summary [file 43018_2023_542_MOESM2_ESM.pdf]

nature portfolio

Corresponding author(s): M. Kok

Last updated by author(s): 31-01-2023

Reporting Summary

Nature Portfolio wishes to improve the reproducibility of the work that we publish. This form provides structure for consistency and transparency in reporting. For further information on Nature Portfolio policies, see our Editorial Policies and the Editorial Policy Checklist.

Statistics

For all statistical analyses, confirm that the following items are present in the figure legend, table legend, main text, or Methods section.

n/a	Confirmed	
☐	☒	The exact sample size (*n*) for each experimental group/condition, given as a discrete number and unit of measurement
☐	☒	A statement on whether measurements were taken from distinct samples or whether the same sample was measured repeatedly
☐	☒	The statistical test(s) used AND whether they are one- or two-sided<br>*Only common tests should be described solely by name; describe more complex techniques in the Methods section.*
☐	☒	A description of all covariates tested
☐	☒	A description of any assumptions or corrections, such as tests of normality and adjustment for multiple comparisons
☐	☒	A full description of the statistical parameters including central tendency (e.g. means) or other basic estimates (e.g. regression coefficient) AND variation (e.g. standard deviation) or associated estimates of uncertainty (e.g. confidence intervals)
☐	☒	For null hypothesis testing, the test statistic (e.g. *F*, *t*, *r*) with confidence intervals, effect sizes, degrees of freedom and *P* value noted<br>*Give P values as exact values whenever suitable.*
☒	☐	For Bayesian analysis, information on the choice of priors and Markov chain Monte Carlo settings
☒	☐	For hierarchical and complex designs, identification of the appropriate level for tests and full reporting of outcomes
☒	☐	Estimates of effect sizes (e.g. Cohen's *d*, Pearson's *r*), indicating how they were calculated

Our web collection on statistics for biologists contains articles on many of the points above.

Software and code

Policy information about availability of computer code

Data collection	Clinical data was collected in electronic case report forms, additional data was collected by the primary authors in spreadsheets. Sequencing data was generated from primary tumors and biopsies of metastatic lesions as described in the Methods.
Data analysis	DNA Sequencing data was aligned to GRCh38 reference genome with bwa aligner 0.7.17 64 using the bwa-mem algorithm. Samtools fixmate 1.13 65 was used to correct mate information, and duplicate reads were marked with Picard MarkDuplicates. Next, base quality scores were recalibrated with GATK BaseRecalibrator 66, and Mutect2 2.2 67 was used to perform variant calling. The data that passed all Mutect2 filters was subsequently filtered with fings 1.7.1 68 and vcf2maf 1.6.21 69 was used to run VEP annotation of the variants and to produce a maf file. Variants with VAF>0.2 were included in final analysis. TMB was calculated with maftools 2.10.5 70 tmb function. VAF plots, mutational signature plots and oncoplot were created with maftools 2.10.5. Data was analyzed with Python 3.7.6 and R 4.1.1. Pandas 1.3.3 [21, 22] was used for data handling.<br>RNA sequencing data has been aligned to GRCh38 with STAR 2.7.1a, with twopassMode option set to “Basic” 71. Gene counts were obtained with STAR quantMode option set to “GeneCounts”. Data quality was assessed with FastQC 0.11.5 72, FastQ Screen 0.14.0 73, Picard CollectRnaSeqMetrics tool 74 and RSeQC read_distribution.py and read_duplication.py tools 4.0.0 75 and found to be suitable for the downstream analysis. Data was subsequently normalised to TPM. For cell deconvolution, CIBERSORTx was run in absolute mode with LM22 Source GEP, performing the batch correction 76. Differential expression analysis was performed with DESeq2 1.34.0 77. PAM50 classification was performed with genefu R package 2.26.0 78. Gseapy 0.9.18 ssgsea tool 79 with sample_norm_method option set to “rank” was used for gene set signature scoring. Data was analyzed with Python 3.7.6 and R 4.1.1. Pandas 1.3.3 80,81 and NumPy 1.18.1 82 were used for data handling. Seaborn 0.10.0 83, Matplotlib 3.1.3 84 and statannotations 0.4.3 85 have been used for plotting.<br>All other analysis were done with GraphPad Prism v9.0, IBM SPSS statistics 24 or SAS v9.4.

For manuscripts utilizing custom algorithms or software that are central to the research but not yet described in published literature, software must be made available to editors and reviewers. We strongly encourage code deposition in a community repository (e.g. GitHub). See the Nature Portfolio guidelines for submitting code & software for further information.

Data

Policy information about availability of data

All manuscripts must include a data availability statement. This statement should provide the following information, where applicable:

- Accession codes, unique identifiers, or web links for publicly available datasets
- A description of any restrictions on data availability
- For clinical datasets or third party data, please ensure that the statement adheres to our policy

DNA and RNA sequencing data are stored in the European Genome-Phenome Archive (EGA) under the accession code EGAS00001006902. Sequencing data and source data supporting the findings of this study are not publicly available due to the clinical trial agreements and will be made available from the corresponding author upon reasonable request. Data requests will be reviewed by the corresponding author and Institutional Review Board of the NKI and after approval applying researchers have to sign a data transfer agreement with the NKI.

Human research participants

Policy information about studies involving human research participants and Sex and Gender in Research.

Reporting on sex and gender	Both male and female participants could be included in the trial, but only biologically female patients participated in the trial.
Population characteristics	All patients (n = 23) had confirmed metastatic lobular breast cancer (morphology and negative/aberrant e-cadherin staining). The median age of the patients was 60 years old, with 78% of patients receiving chemotherapy in either the primary tumor setting and/or palliative setting. 78% of patients had visceral metastasis and 52% had liver metastasis.
Recruitment	Patients were recruited in four centers in the Netherlands. All were high-volume breast cancer centers, and three were academic centers. Additionally, patients could only be included when they had a lesion available for serial biopsies, excluding ILC patients with bone-only disease. This selection criterium could have led to recruitment of ILC patients with a poor prognosis.
Ethics oversight	Medical-ethical committee of the Netherlands Cancer Institute approved the study protocol, informed consent form and amendments with local oversight in each centre.

Note that full information on the approval of the study protocol must also be provided in the manuscript.

Field-specific reporting

Please select the one below that is the best fit for your research. If you are not sure, read the appropriate sections before making your selection.

☒ Life sciences ☐ Behavioural & social sciences ☐ Ecological, evolutionary & environmental sciences

For a reference copy of the document with all sections, see nature.com/documents/nr-reporting-summary-flat.pdf

Life sciences study design

All studies must disclose on these points even when the disclosure is negative.

Sample size	A Simon's two-stage design was used to determine the sample size. The median PFS of palliative chemotherapy regimens in patients with endocrine-treatment refractory breast cancer typically lies between 2-4 months 60,61. If 25% of patients were free of progression at 6 months in the GELATO-trial, this would warrant further investigation of the treatment regimen. The null hypothesis that the true proportion of patients progression-free at six months is 10% or lower will be tested against a one-sided alternative of at least 25%. In the first stage of the trial, 22 patients had to be accrued. If there were two or fewer patients progression-free at six months the study would be stopped, otherwise an additional 18 patients could be included. This design yields a type one error rate of 0.04 and power of 0.80 when the true proportion of patients progression-free at six months is 25%. The last two patients were registered simultaneously and therefore both included in the trial, leading to a total inclusion of 23 patients.
Data exclusions	37 patients were screened for participation of which 11 were not registered due to: no biopsy site available, no clear ILC or rapid disease progression. Three additional patients did not receive atezolizumab due to rapid clinical progression or worsening of pre-existent cardiomyopathy (see Extended Data Figure 1) and were also excluded from the analysis.
Replication	Since this study is a single-arm trial, the clinical findings cannot be directly replicated.
Randomization	GELATO is a single-arm trial, with no randomization or stratification applied.
Blinding	The GELATO-trial is an investigator-initiated clinical trial with the primary investigators frequently being the treating physicians. The investigators were therefore not blinded for outcome.

March 2021

Reporting for specific materials, systems and methods

We require information from authors about some types of materials, experimental systems and methods used in many studies. Here, indicate whether each material, system or method listed is relevant to your study. If you are not sure if a list item applies to your research, read the appropriate section before selecting a response.

Materials & experimental systems

n/a	Involved in the study
☐	☒ Antibodies
☒	☐ Eukaryotic cell lines
☒	☐ Palaeontology and archaeology
☒	☐ Animals and other organisms
☐	☒ Clinical data
☒	☐ Dual use research of concern

Methods

n/a	Involved in the study
☒	☐ ChIP-seq
☐	☒ Flow cytometry
☒	☐ MRI-based neuroimaging

Antibodies

Antibodies used

Flow cytometry:
CD3 BUV496 UCHT1 1:100 BD Bioscience 612940
CD4 BV421 RPA-T4 1:100 BD Bioscience 562424
CD8 BUV805 SK1 1:200 BD Bioscience 612754
Pan TCR PE 11F2 1:100 BD Bioscience 555717
FoxP3 PE Cy5.5 FJK-16s 1:50 eBioscience/Thermofisher 35-5773-82
CCR7 APC R700 150503 1:50 BD Bioscience 565868
CD45RA BUV737 HI100 1:400 BD Bioscience 612846
CD25 AF647 BC96 1:100 BioLegend 302618
PD-1 APC Cy7 EH12.2H7 1:100 BioLegend 329922
CTLA-4 PE CF594 BNI3 1:200 BD Bioscience 562742
v1 FITC TS8.2 1:100 Thermofisher TCR2730
v2 BUV395 B6 1:100 BD Bioscience 748582
CD19 PE Cy5 HIB19 1:200 BD Bioscience 555414
CD3 PE Cy5 UCHT1 1:200 BD Bioscience 555334
CD56 PE Cy5 B159 1:100 BD Bioscience 555517
CD161 PE Cy5 DX12 1:100 BD Bioscience 551138
HLA-DR BUV661 G46-6 1:100 BD Bioscience 612980
CD14 BUV737 M5E2 1:100 BD Bioscience 612763
CD16 BUV496 3G8 1:100 BD Bioscience 612944
CD11c BV785 3.9 1:100 BioLegend 301644
CD1c PE Cy7 L161 1:100 BioLegend 331516
CD141 BV711 1A4 1:100 BD Bioscience 563155
CD123 PE 6H6 1:200 BioLegend 396604
CD66b AF647 G10F5 1:200 BD Bioscience 561645
CD33 PerCP Cy5.5 WM53 1:100 BioLegend 303414
CD303 APC vio770 REA693 1:100 Miltenyi Biotech 130-114-178
CD41a BUV395 HIP8 1:400 BD Bioscience 740295
FcRI PE Dazzle 594 AER-37(CRA-1) 1:200 BioLegend 334634
CD34 FITC 581 1:100 BD Bioscience 555821
CD19 BUV395 SJ25C1 1:50 BD Bioscience 563549
IgD APC IA6-2 1:100 BD Bioscience 561303
CD20 BUV805 2H7 1:200 BD Bioscience 612905
CD27 PE M-T271 1:200 BD Bioscience 555441
CD10 AF700 HI10a 1:200 BD Bioscience 563509
CD24 BB515 ML5 1:200 BD Bioscience 564521
IgM APC Cy7 MHM-88 1:100 BioLegend 314520
CD38 BUV737 HIT2 1:400 BD Bioscience 741837
CD5 PE Dazzle 594 L17F12 1:400 BioLegend 364012
CD1d BV786 42.1 1:200 BD Bioscience 743608
CD138 BV711 MI15 1:200 BioLegend 563184

Immunohistochemistry:
CD8 (clone C8/144B, Agilent / DAKO); PD-L1 (clone SP142, Roche / Ventana)

Validation

All antibodies used are commercially available and validated by manufacturer. Antibodies for flow cytometry were further validated for target species (human) using FMO or isotype controls where necessary.

Clinical data

Policy information about clinical studies

All manuscripts should comply with the ICMJE guidelines for publication of clinical research and a completed CONSORT checklist must be included with all submissions.

Clinical trial registration	NCT03147040
Study protocol	The full trial protocol will be included as Supplementary Note.
Data collection	Patients were included between November 2017 and January 2021. Data was collected in electronic case report forms in each participating center and extracted directly from the patient records by local data managers.
Outcomes	The primary endpoint of the trial was progression-free survival (PFS) rate at six months (24 weeks), assessed from date of registration to date of progression according to RECISTv1.1 or death from any cause. Secondary endpoints were progression-free survival (PFS) rate at six months in patients with immune-related ILC (assessed by gene expression), PFS rate at 12 months, PFS according to iRECIST, overall survival (OS), objective response rate (ORR) and safety. Translational endpoints were the assessment of immunogenic effects of carboplatin on the TME and in the circulation using immunohistochemistry (IHC), next-generation sequencing and flow cytometry; the additive effect of anti-PD-L1 on these changes; and exploration of predictive biomarkers.

Flow Cytometry

Plots

Confirm that:

☒ The axis labels state the marker and fluorochrome used (e.g. CD4-FITC).

☒ The axis scales are clearly visible. Include numbers along axes only for bottom left plot of group (a 'group' is an analysis of identical markers).

☒ All plots are contour plots with outliers or pseudocolor plots.

☒ A numerical value for number of cells or percentage (with statistics) is provided.

Methodology

Sample preparation	Red blood cells were lysed (lysis buffer: dH2O, NH4Cl, NaHCCO3, EDTA) and cells were resuspended in PBS containing 0.5% BSA and 2mM EDTA. For surface antigen staining, cells were first incubated with human FcR Blocking Reagent (1:100 Miltenyi) for 15 min at 4°C and then incubated with fluorochrome-conjugated antibodies for 30 min at 4°C, in the dark. For intracellular antigen staining, cells were fixed with Fixation/Permeabilization solution 1X (Foxp3/Transcription Factor Staining Buffer Set, eBioscience) for 30 min at 4°C and stained with fluorochrome-conjugated antibodies in Permeabilization buffer 1X (eBioscience) for 30 min at room temperature. Viability was assessed by staining with either 7AAD staining solution (1:20; eBioscience) or Zombie Red Fixable Viability Kit (1:800 BioLegend).
Instrument	Data acquisition was performed on BD LSRII flow cytometer using Diva software (BD Biosciences).
Software	Data analysis was performed using FlowJo software version 10.6.2.
Cell population abundance	All cells were selected on viability and to obtain absolute white blood cell counts per mL of human blood, the total post-lysis cell count was obtained using the NucleoCounter NC-200 (Chemometec) Automated cell counter. All assessed cell populations (Supplementary Table 5) were abundant in the peripheral blood of the patients and only major cell subsets were assessed.
Gating strategy	The full gating strategy is listed as Extended Data Figure 8.

☒ Tick this box to confirm that a figure exemplifying the gating strategy is provided in the Supplementary Information.
