# Supplementary material for: PD-L1 blockade in combination with carboplatin as immune induction in metastatic lobular breast cancer: the GELATO trial
Source: Nat Cancer. 2023 Apr 10;4(4):535–49. doi: 10.1038/s43018-023-00542-x (PMC10132987; doi:10.1038/s43018-023-00542-x)
Supplement: Supplementary file 3 — Supplementary Tables 1–6. [file 43018_2023_542_MOESM3_ESM.pdf]

Supplementary Table 1: Worst grade of any treatment-related adverse event. Immune-related events are a selection of all reported atezolizumab-related events. Only grade 2 events or higher or grade 1 immune-related events were reported. 1 patient died during treatment of disease progression.

Worse grade of any adverse event, no. of patients (%)	No AE reported	Grade 1-2	Grade 3	Grade 4
**Carboplatin-related**	6 (26)	7 (30)	9 (39)	1 (4)
**Atezolizumab-related**	11 (48)	5 (22)	5 (22)	2 (9)
**Immune-related event of interest**	13 (57)	4 (17)	5 (22)	1 (4)

Supplementary Table 2: all reported carboplatin-related toxicity. Only grade 2 events or higher were reported. Alanine aminotransferase (ALAT).

Carboplatin related adverse event, no. of patients (%)	Any grade	Grade 3	Grade 4
**Neutrophil count decreased**	11 (48)	3 (13)	1 (4)
**Anemia**	5 (22)	2 (9)	0 (0)
**Platelet count decreased**	3 (13)	2 (9)	0 (0)
**Nausea**	3 (13)	0 (0)	0 (0)
**Fatigue**	2 (9)	0 (0)	0 (0)
**Abdominal pain**	1 (4)	1 (4)	0 (0)
**ALAT increased**	1 (4)	1 (4)	0 (0)
**Dehydration**	1 (4)	1 (4)	0 (0)
**Flu-like symptoms**	1 (4)	0 (0)	0 (0)
**Gastro-esophageal reflux**	1 (4)	0 (0)	0 (0)
**Hyperkaliemia**	1 (4)	0 (0)	0 (0)
**Hyponatremia**	1 (4)	1 (4)	0 (0)
**Infusion-related reaction**	1 (4)	0 (0)	0 (0)
**Increased lipase**	1 (4)	0 (0)	0 (0)
**Obstipation**	1 (4)	0 (0)	0 (0)
**Peripheral neuropathy**	1 (4)	0 (0)	0 (0)

Supplementary Table 3: immune-related adverse events of special interest. Alanine aminotransferase (ALAT); aspartate aminotransferase (ASAT). *1 patient developed a grade 3 hypophysitis two months after stopping atezolizumab. ^Asymptomatic without signs of (immune-related) pancreatitis

Immune related adverse event, no. of patients (%)	Any grade	Grade 3	Grade 4
**ASAT increased**	4 (17)	2 (9)	1 (4)
**ALAT increased**	2 (9)	2 (9)	0 (0)
**Flu-like symptoms**	2 (9)	0 (0)	0 (0)
**Hypophysitis***	2 (9)	1 (4)	0 (0)
**Lipase increased**^	2 (9)	1 (4)	0 (0)
**Colitis**	1 (4)	1 (4)	0 (0)
**Dry mouth**	1 (4)	0 (0)	0 (0)
**Dry skin**	1 (4)	0 (0)	0 (0)
**Myalgia**	1 (4)	0 (0)	0 (0)
**Sarcoid-like reaction**	1 (4)	1 (4)	0 (0)

Supplementary Table 4: characteristics of patients with collected primary tumors. [1]For 1 patient with a secondary primary tumor, only the second tumor was collected (first tumor was not ILC). For 1 patient with an isolated contralateral recurrence, the first primary tumor and contralateral recurrence were collected. [2]4/5 patients with a triple-negative metastasis had a primary ER+ tumor. 1 of these patients had a triple-negative contralateral recurrence. Two out of 17 patients with de novo metastatic disease had ER+ disease. [3]including mixed classical and pleiomorphic. [4]Local recurrence including recurrence in skin. 1 patient had a local ILC recurrence and contralateral breast cancer of no special type simultaneously. [5]Breast lesions are compared with synchronous distant metastasis.

**N = 17 available patients**		**No. (%)**
**Age at diagnosis, years**	Median (range)	51 (33-65)
**Histological subtype (assessed on primary tumor[1,2])**	ER+HER2-<br>ER+HER2+	16 (94)<br>1 (6)
**Molecular subtype (assessed on primary tumor, n=10)**	Luminal A<br>Luminal B<br>HER2-enriched	4 (40)<br>5 (50)<br>1 (10)
**Histological grade primary tumor**	Grade 1<br>Grade 2<br>Grade 3<br>Unknown	2 (12)<br>9 (53)<br>3 (18)<br>3 (18)
**Tumor stage at diagnosis**	T1<br>T2<br>T3	4 (24)<br>8 (47)<br>5 (29)
**Nodal stage at diagnosis**	N0<br>N1<br>N2<br>N3	5 (29)<br>5 (29)<br>2 (12)<br>5 (29)
**Neo-adjuvant chemotherapy**		6 (35)
**Response to neo-adjuvant chemotherapy**	pCR<br>pPR (1-50% tumorrest)<br>pNR (>50% tumorrest or no response)	0 (0)<br>1 (17)<br>5 (83)
**Disease course**	Primary-metastasis<br>Primary-local recurrence-metastasis[4]<br>De novo M1[5]<br>Primary-2nd primary-metastasis[1]<br>Primary-contralateral recurrence-metastasis[1]	10 (59)<br>3 (18)<br>2 (12)<br>1 (6)<br>1 (6)
**Biopsy site GELATO-trial**	Liver<br>Lymph node<br>Peritoneum<br>Cervix<br>Skin<br>Thoracal wall	7 (41)<br>4 (24)<br>3 (18)<br>1 (6)<br>1 (6)<br>1 (6)

Supplementary Table 5: list of measured circulating immune cell populations as displayed in Figure 4A-B and Extended Data Figure 5.

**Circulating immune cell populations**
Eosinophils
Basophils
Neutrophils
$CD141^{high}$ dendritic cells (DCs)
$CD1c^{+}$ DCs
$CD1c^{-}$ DCs
Plasmacytoid DCs
$CD14^{+}CD16^{-}$ monocytes
$CD14^{dim}$ monocytes
Total T cells
Conventional $CD4^{+}$ T cells (within total T cells)
$CD8^{+}$ T cells (within total T cells)
Regulatory T cells (within total T cells)
Double positive T cells (within total T cells)
vδ1 γδ T cells (within total T cells)
vδ2 γδ T cells (within total T cells)
Total B cells
Double negative B cells (within total B cells)
Non-switched memory B cells (within total B cells)
IgM-only memory B cells (within total B cells)
Switched memory B cells (within total B cells)
Plasmacells/blasts (within total B cells)
Naïve B cells (within total B cells)

Supplementary Table 6: list of antibodies used for flow cytometry

Antigen	Fluorochrome	Clone	Dilution	Company	Catalogue number
CD3	BUV496	UCHT1	1:100	BD Bioscience	612940
CD4	BV421	RPA-T4	1:100	BD Bioscience	562424
CD8	BUV805	SK1	1:200	BD Bioscience	612754
Pan γδ TCR	PE	11F2	1:100	BD Bioscience	555717
FoxP3	PE Cy5.5	FJK-16s	1:50	eBioscience/Thermofisher	35-5773-82
CCR7	APC R700	150503	1:50	BD Bioscience	565868
CD45RA	BUV737	HI100	1:400	BD Bioscience	612846
CD25	AF647	BC96	1:100	BioLegend	302618
PD-1	APC Cy7	EH12.2H7	1:100	BioLegend	329922
CTLA-4	PE CF594	BNI3	1:200	BD Bioscience	562742
vδ1	FITC	TS8.2	1:100	Thermofisher	TCR2730
vδ2	BUV395	B6	1:100	BD Bioscience	748582
CD19	PE Cy5	HIB19	1:200	BD Bioscience	555414
CD3	PE Cy5	UCHT1	1:200	BD Bioscience	555334
CD56	PE Cy5	B159	1:100	BD Bioscience	555517
CD161	PE Cy5	DX12	1:100	BD Bioscience	551138
HLA-DR	BUV661	G46-6	1:100	BD Bioscience	612980
CD14	BUV737	M5E2	1:100	BD Bioscience	612763
CD16	BUV496	3G8	1:100	BD Bioscience	612944
CD11c	BV785	3.9	1:100	BioLegend	301644
CD1c	PE Cy7	L161	1:100	BioLegend	331516
CD141	BV711	1A4	1:100	BD Bioscience	563155
CD123	PE	6H6	1:200	BioLegend	396604
CD66b	AF647	G10F5	1:200	BD Bioscience	561645
CD33	PerCP Cy5.5	WM53	1:100	BioLegend	303414
CD303	APC vio770	REA693	1:100	Miltenyi Biotech	130-114-178
CD41a	BUV395	HIP8	1:400	BD Bioscience	740295
FcεRIα	PE Dazzle 594	AER-37(CRA-1)	1:200	BioLegend	334634
CD34	FITC	581	1:100	BD Bioscience	555821
CD19	BUV395	SJ25C1	1:50	BD Bioscience	563549
IgD	APC	IA6-2	1:100	BD Bioscience	561303
CD20	BUV805	2H7	1:200	BD Bioscience	612905
CD27	PE	M-T271	1:200	BD Bioscience	555441
CD10	AF700	HI10a	1:200	BD Bioscience	563509
CD24	BB515	ML5	1:200	BD Bioscience	564521
IgM	APC Cy7	MHM-88	1:100	BioLegend	314520
CD38	BUV737	HIT2	1:400	BD Bioscience	741837
CD5	PE Dazzle 594	L17F12	1:400	BioLegend	364012
CD1d	BV786	42.1	1:200	BD Bioscience	743608
CD138	BV711	MI15	1:200	BioLegend	563184
